# High-Throughput Miniaturized Biotransformation Testing Using Activated Sludge Enables Rapid Chemical Persistence Assessment

**DOI:** 10.1021/acs.estlett.5c00859

**Published:** 2025-10-27

**Authors:** Sarah B. Partanen, Nicolas Mueller, Kathrin Fenner

**Affiliations:** † Swiss Federal Institute of Aquatic Science and Technology, 28499 Eawag, 8600 Dubendorf, Switzerland; ‡ Department of Chemistry, University of Zurich, 8057 Zurich, Switzerland

**Keywords:** SSbD (Safe and Sustainable by Design), biotransformation, persistence assessment, well plates, environmental fate, chemical screening, biodegradation

## Abstract

The unprecedented scale and pace of chemical development challenges human and ecosystem health unless new chemicals are developed using safe-by-design approaches. Therefore, tools for efficient environmental persistence assessment—among other critical assessment capabilities—are urgently needed, as outlined in the European Commission’s Safe and Sustainable by Design (SSbD) framework and the European Chemical Agency (ECHA)’s 2025 report on key regulatory challenges. Current persistence tests require large sample amounts and extended timelines making them unsuitable for early stage chemical development. We developed and validated a miniaturized, higher-throughput biotransformation assay using municipal activated sludge as the source of microbial inoculum. For 33 pesticides and pharmaceuticals, biotransformation rate constants showed strong correlation with large volume controls (R^2^ > 0.84) and consistent relative biotransformation rankings across time and different sources of activated sludge (Spearman correlations > 0.8). Our 24-well plate test requires 2 mL per test (vs hundreds of mL in standard tests) and provides biotransformation data within 48 h (vs weeks or months) due to the dense biomass and high bioavailability of substrates in our targeted substance space (i.e., log *K*_oc_ ≲ 4). This miniaturized test lends itself to further automation and enables persistence assessment during chemical design, directly supporting SSbD principles.

## Graphical abstract

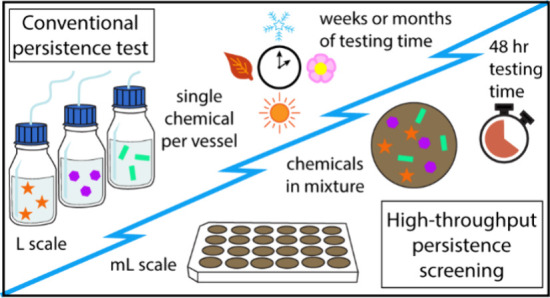

## Introduction

Globally, chemical production and use have reached unprecedented levels, with hundreds of thousands of products currently on the market and new ones being developed daily.^1^ Some suggest that the world has surpassed the safe operating space relating to these “novel entities”, threatening human and environmental health.^2^ However, the extent of the problem is not well characterized, because comprehensive risk assessments do not exist for the majority of these chemicals,^3^ resulting in a major knowledge gap regarding their impacts. Further, the sheer magnitude of chemicals in existence and under development mean that performing risk assessments one by one is not feasible.^4^

In Europe, the Safe and Sustainable by Design (SSbD) framework calls for the development of innovative tools to assess chemical hazard and risk more efficiently, especially during new chemical design.^5^ A growing number of regulators and researchers echo the need for such tools,^6,7^ notably the 2025 European Chemical Agency (ECHA) report on key regulatory challenges.^6^ Among the regulatory priorities, persistence assessment is particularly critical in addressing chemical pollution, as highly persistent chemicals lead to increasing and long-lasting contamination, which could be associated with serious adverse effects.^8^

Biodegradation typically represents the most important pathway for chemical attenuation in the environment, making biodegradation testing central to persistence assessment.^9^ However, current tests are poorly suited to the SSbD framework and early stage chemical development, requiring large reactor volumes (up to 5000 mL), high chemical concentrations (10–400 mg_C_/L in screening tests), single-compound testing, and extended timelines (weeks to months).^10–16^ Two approaches have attempted to address these limitations, each with partial success. Miniaturized assays following dissolved oxygen (DO) consumption^17,18^ or colorimetric end points applicable to specific compound classes^19^ achieve smaller volumes and higher throughput but retain the fundamental limitations of ready biodegradability tests: non-environmentally relevant concentrations, single-compound testing, and extended test durations. Alternatively, rapid screening methods^20^ provide faster results but have primarily focused on testing removal of industrial chemicals during wastewater treatment. Neither approach provides the combination of miniaturization, throughput, and kinetic information needed for early stage chemical development.

For the chemical industry to adopt persistence testing earlier during development, new high-throughput methods must provide biotransformation rate constants that relate to regulatory persistence end points, are obtained at environmentally relevant concentrations, and accommodate diverse chemical structures. Structurally complex specialty chemicals including pesticides and pharmaceuticals are typically not readily biodegradable, requiring kinetic information from simulation studies (OECD 307, 308)^12–14^ for persistence assessment. Since we have previously demonstrated that biotransformation rate constants from large volume activated sludge (AS) experiments predict primary biotransformation half-lives from simulation studies,^21,22^ and complementing previous developments of miniaturized ready biodegradability assays, we developed a miniaturized assay measuring primary biotransformation to support persistence screening for specialty chemicals in an SSbD context.

Our approach addresses the practical constraints of early stage development including limited sample quantities and the need for rapid decision making. The assay uses municipal activated sludge as microbial inoculum and employs a 24-well plate format requiring only 2 mL of sample volume and sub-μg chemical quantities. The assay provides results within 48 h while enabling simultaneous testing of 30+ compound mixtures. Our objectives are to optimize the miniaturized test format, demonstrate its robustness and correlation with large volume controls, and to identify opportunities for analytical workflow automation.

## Materials and Methods

### Compound Selection

Test compounds consisted of 33 pesticides and pharmaceuticals (Supporting Information (SI) Table S1.1) chosen for their published transformation rate constants in larger AS systems (50–100 mL) and availability of half-lives from standard soil and sediment tests for some compounds.^21,23^ The compounds cover a broad range of physicochemical properties (log *K*_oc_ = 1.34–4.98, log D at pH 7.4 = −2.26–4.48), and are nonvolatile and water-soluble at the test concentration of 6 μg/L.

### Microbial Inoculum Selection

Municipal AS was selected for method development and validation. When paired with environmentally relevant substrate concentrations (5–10 μg/L), AS biotransformation assays produce measurable first-order rate constants (*k*_deg_) within 48 h with minimal lag-phase.^22–28^ This approach provides environmental persistence proxies through read-across relationships between AS kinetics and soil/water-sediment half-lives.^21,22^

### Site Selection

AS from six Swiss wastewater treatment plants (WWTPs) was used in biotransformation experiments (SI Table S1.2), with Neugut WWTP (Dübendorf) providing AS for all experiments. One experiment compared all six sources to assess intersource variability. Selection criteria (>10,000 population equivalent, nitrifying/denitrifying process, < 10% non-municipal influent) are detailed in the SI.

### Activated Sludge Biotransformation Experiments

Biotransformation experiments (June 2023–September 2024) are detailed in SI Section S1.3 and Table S1.3. Test compound mixtures in ethanol (SI Table S1.1; mixture testing in well plates validated in SI Figure S1.1) were spiked to multiwell plates (24-, 48-, 96-, and 96-deep-well with 2 mL, 1 mL, 300 μL, and 1 mL of AS respectively) or 50 mL controls in Schott bottles, representing the typical volume scale of 10s-100s of mL used in AS studies.^16,22,23,28^ After solvent evaporation and redissolution of compounds in AS supernatant, AS was added to achieve initial compound concentrations of 6.0 μg/L in each vessel or well. Seven samples were collected over 48 h for kinetic analysis. Replicate vessels (Table S1.3) were incubated using either an orbital (Labwit, 150 rpm, 20 °C), or plate shaker (BioShake iQ, variable rpm, room temperature). Sorption controls (autoclaved AS) distinguished physical sorption from biotransformation; abiotic controls (autoclaved AS supernatant) confirmed negligible abiotic degradation.

### Test Compound Analysis

After processing, time point samples were analyzed using a Thermo Scientific UltiMate 3000 UHPLC coupled to a Thermo Scientific Q Exactive Hybrid Quadrupole-Orbitrap mass spectrometer (Thermo Fisher Scientific). A justification of the analytical approach, sample processing details and the full instrument method can be found in SI Sections S1.3–S1.4. In brief, full-scan mass spectra in positive mode were obtained over the course of a 6 min gradient at a resolution of 70,000.

Raw LC-HRMS data were processed using the open-source software Skyline (https://skyline.ms) and further analyzed in RStudio. The derivation of *k*_deg_ values and area-time series plots for the test compounds at multiple scales (24-, 48-, and 96-well plate, and large volume control) are provided in SI Section S1.4 and Figure S2.1.

## Results and Discussion

### Experimental Optimization

Using AS as a test system, we systematically compared well plate scales (24-, 48-, 96-, and 96-deep-well) and identified 24-well plates as the optimal scale for the miniaturized biotransformation assay (Figure S2.3). The 24-well plate produced representative and reproducible *k*_deg_ values that correlated well to a large volume control, verified across multiple independent experiments (R^2^ ≥ 0.84, Figure 1.A).

**Figure 1:**
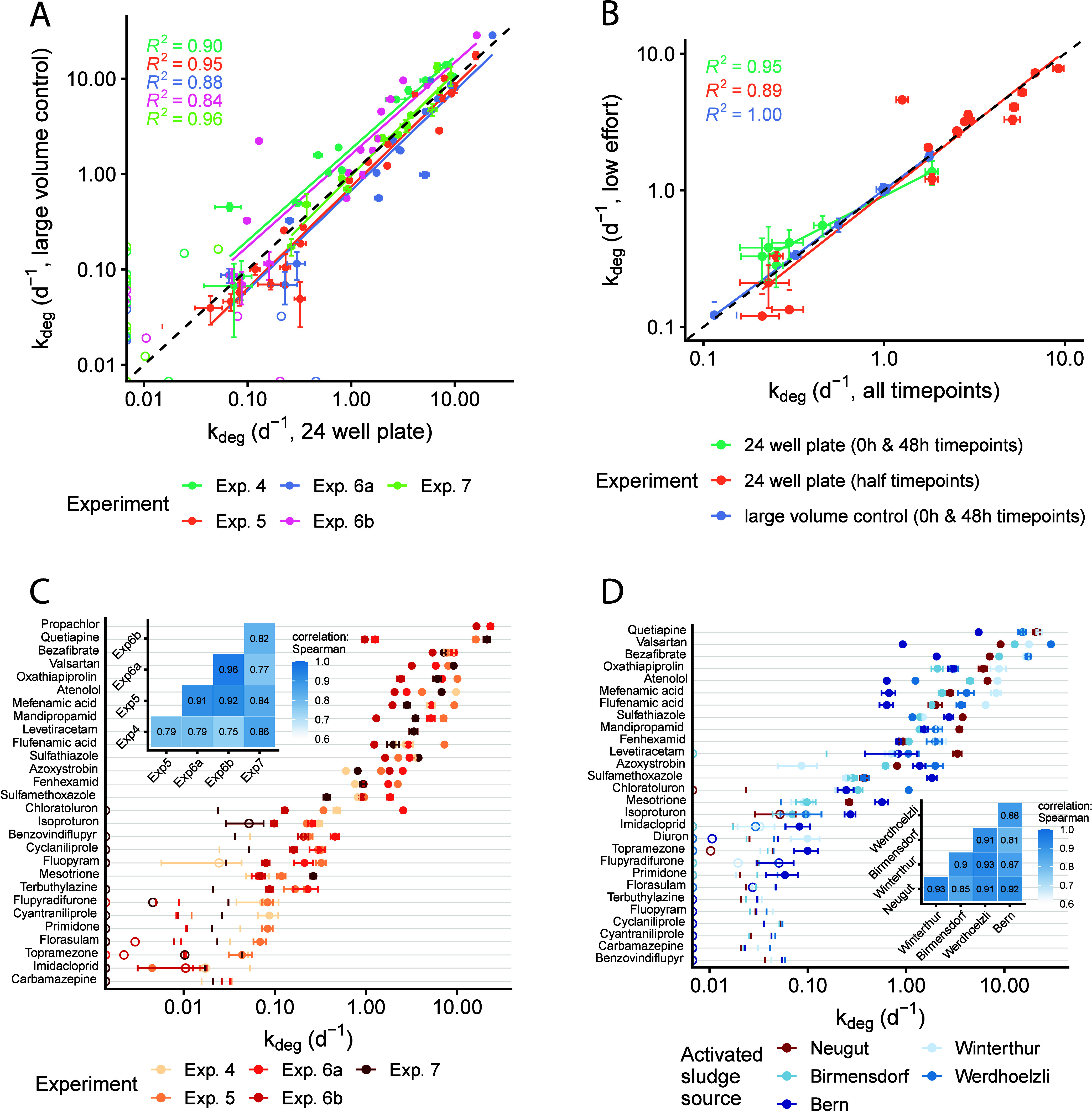
(A) Correlation of *k*_deg_ values obtained from 24-well plates with those obtained from a large volume control across four independent experiments using freshly collected Neugut AS, demonstrating consistency of the core method despite minor variations in experimental detail (e.g., Experiment 6 included both plate shaker (6a) and orbital shaker (6b)). The black dashed line is the 1:1 line, and the colored lines are the lines of best fit. (B) Correlation of *k*_deg_ values determined using a full time series (*x* axis) with those determined using techniques to reduce experimental and analytical effort (*y* axis). Data from experiment 6 (24-well plates mixed in a plate shaker). The black dashed line is the 1:1 line, and the colored lines are the lines of best fit. (C) Variability in biotransformation rate constants across the same four independent experiments shown in panel A. Inset plot: Spearman rank correlations between the different experiments. (D) Variability in biotransformation rate constants across five different sources of AS. Data from experiment 7. Inset plot: Spearman rank correlations between the different AS sources. For all panels, see SI Table S1.3 for further experimental details. Open circles represent *k*_deg_ values smaller than *k*_deg,min_ (see SI section S1.4 for definition) and were not included in any regressions. Error bars represent the standard error of the slope parameter (*k*_deg_) from the global linear regression fitted to replicate time series data. Negative *k*_deg_ values were set to zero, shown as open circles at the left edge of the plots. Tick marks to the right of the *k*_deg_ values set to zero represent the positive extent of the standard error.

We hypothesized three main challenges during development of the miniaturized test: First, potential microbial lottery effects where the smaller absolute quantity of biomass in each well could lead to the inclusion or exclusion of specific degraders by chance alone during sampling.^28,29^ Second, mixing limitations in well plates, which could lead to reduced bioavailability as the AS stratifies in the wells.^30^ Finally, compound loss due to sorption to well plate material leading to challenges in data interpretation.^31^ Each scale showed different behavior with respect to these challenges.

We expected that any microbial lottery effect would be most pronounced in the 96-well plate, as this was the smallest scale tested, but we saw no significant difference in *k*_deg_ value variability in the 96-well plate compared to either the 24-well plate or the large volume control (SI Figure S2.4). The microbial lottery could also be pronounced for those compounds which are suspected to be catabolically degraded by specific microorganisms,^32^ but across the three scales we saw no significant difference in *k*_deg_ value variability for groups of compounds with suspected differences in biotransformation mechanism (SI Figure S2.5 and accompanying text). Thus, we conclude that for a dense and complex community of microorganisms like AS, the microbial lottery does not play a significant role in biotransformation behavior in well plate systems.

Achieving complete mixing in well plates became challenging as well volume decreased, leading to increased variability in replicate *k*_deg_ values due to diffusion limitations in stratified systems. A plate shaker with a 2 mm orbital radius was required to avoid settling of the AS flocs in 96- and 48-well plates and resulted in lower variability in *k*_deg_ values across replicates compared to the same experiments run on an orbital shaker. The geometry of the 96 deep-well plate prevented complete mixing even on a plate shaker, therefore this experimental scale was not investigated further. In the 24-well plate, the 2 mL well volume allowed complete mixing to be achieved on both shaker types. SI Figure 2.6 shows the impact of the plate shaker on inter-replicate *k*_deg_ value variability across scales.

Strong initial sorption was observed at all experimental scales for many semipolar and apolar compounds. Initial sorption within the first hour reduced starting concentrations for several compounds (see sorption controls, SI Figures S2.1 and S2.2). However, in 24-well plates and large volume controls, sorption equilibrium was reached by the time of initial sampling, allowing all time points to be used in kinetic analysis and enabling reliable biotransformation kinetics to be calculated. In contrast, in 96-well plates some compounds showed continued sorption throughout the 48-h experiment (e.g., SI Figure S2.1, azoxystrobin), presumably due to additional diffusion into the well plate material, preventing determination of degradation kinetics for up to half of the test set. To address these sorption limitations, we explored direct aqueous spiking as an alternative compound introduction method. This approach was found to reduce initial sorption to well plates, yet led to increased solvent concentrations. Results showed that additions of up to 0.06% ethanol did not significantly impact *k*_deg_ values (SI Figures S2.7–S2.10 and accompanying text), offering a potential improvement to the workflow.

The 96-well plates, while providing the largest opportunity for increased testing throughput, had some fundamental challenges when used for biotransformation testing. While there was no evidence of microbial lottery effects at this (or any) scale, and bioavailability concerns were minimized when a plate shaker was used to achieve complete mixing, sorption artifacts at this scale were too pronounced to make it a viable option. Further, sacrificial sampling of wells at each subsampling time was required in the 96-well plate whereas an entire time series could be taken from one 2 mL well in a 24-well plate. The 24-well plate is easy to mix in either a plate or orbital shaker and shows similar extents of sorption as the large volume control under the conditions tested here. While sorption behavior may vary with different experimental conditions or compound properties, the 24-well plate did not exhibit the time-dependent sorption artifacts observed in the 96-well plates for the compounds successfully analyzed in this work. Therefore, the 24-well plate scale provides the optimal balance between increased throughput and reproducible and reliable *k*_deg_ values.

### Data Robustness and Transferability

The absolute variability in *k*_deg_ values for a given compound can be up to an order of magnitude, whether measured across multiple experiments with the same AS source (Figure 1C) or across multiple AS sources at a single point in time (Figure 1D). This finding reflects the intrinsic variability in environmental parameters associated with biotransformation experiments, including the density and composition of the microbial inoculum at the time of sampling, the operating conditions of the wastewater treatment plant, and seasonal changes such as temperature.

Although the absolute *k*_deg_ values may vary, the relative biodegradability of the compounds tested is very robust, both over time and across different AS sources, with Spearman rank correlation coefficients generally above 0.8 (Figure 1C and D, inset plots). The relative biotransformation behavior of our test compounds has two important implications for the wide adoption of the proposed workflow. First, the robust rankings we saw were achieved using the WWTP selection criteria we defined in this work, demonstrating their utility in bounding the type and quality of AS used in the assay. Second, the robust rankings indicate that a standardized set of benchmark compounds could enable interlaboratory comparisons and quality control.^33^ Further work will focus on which compounds are most suitable for this type of benchmark compound set.

### Analytical Workflow Efficiency

Along with an increase in experimental throughput comes an increase in samples and data to analyze. For a persistence screening test to be truly compatible with SSbD principles, techniques for reducing the time and personnel effort required for analysis must be explored.

For sample analysis, reducing the length of the LC-HRMS method is crucial for increasing throughput. The method used in this work takes 6 min per sample (see SI section S1.4 for details), enabling measurement of 100 experimental samples within 15 h.

Another opportunity to reduce analytical effort is to measure fewer subsamples over the course of the experiment. We explored two ways of using fewer subsamples to determine *k*_deg_ values; first, from a regular time series of seven subsamples, we calculated *k*_deg_ values using SI eq S3, that is using only initial and final peak areas. Second, we conducted an experiment with four subsamples over the course of the experiment rather than the normal seven. In both cases, *k*_deg_ values correlated well to those determined using a full time series (Figure 1B). However, some data is lost in using only initial and final peak areas, as kinetic information cannot be obtained for compounds that are fully degraded by or before 48 h. Further, advance knowledge of the degradation behavior of the compounds is required to verify the assumption of first-order kinetics with minimal lag-phase. Therefore, taking four subsamples represents a good balance between efficiency and data quality.

We used the open-source program Skyline to explore data processing efficiencies. *k*_deg_ values determined directly from LC-HRMS peak areas rather than from calculated compound concentrations were not significantly different across many samples of AS, both over time and for multiple WWTPs (SI Figure S2.11). This equivalence is further demonstrated by concentration–time series plots for nine compounds spanning a range of log *K*oc values (1.87–4.14) that had available isotope-labeled standards (SI Figure S2.2). However, isotope-labeled standards are typically unavailable in a chemical development context, making peak area analysis a practical approach. We acknowledge that data interpretation becomes more complex for strongly sorbing compounds when using peak areas alone, as a lack of loss over time could reflect either recalcitrance to enzymatic degradation or strong sorption and thus reduced bioavailability. Conversely, disappearance due to strong sorption or fast biotransformation becomes difficult to differentiate. While this represents a current limitation for substances with log *K*oc values ≳ 4, work is ongoing in our laboratories to expand the applicable chemical space of the workflow toward strongly sorbing substances by using dilute sludge inocula and full sample extraction.

Next, we explored reduction or elimination of manual peak integration of the raw chromatographic data obtained from the LC-HRMS. Comparing *k*_deg_ values obtained from peak areas that were manually adjusted to those from the default peak area integrations in Skyline, we found good agreement and no significant increase in mean relative error of replicate measurements (SI Figures S2.12 and S2.13). However, integration failures resulted in either missing or incorrectly calculated *k*_deg_ values for six compounds (Figure S2.14 and accompanying text). We are developing automated flagging criteria based on replicate peak variability (CV thresholds) and unexpected kinetic trajectories to identify compounds requiring manual review, minimizing both data loss and incorrect calculations.

Compared to established OECD methods where one compound is tested at a time,^12–14^ well-plate-based systems generate substantially higher data volumes that require efficient analytical workflows. The techniques outlined here are essential complements to the increased experimental throughput achieved through miniaturization. While these strategies may result in some data loss for a minority of compounds (e.g., strongly sorbing compounds, or incorrect automatic peak integration), the rapid availability of biotransformation information across large compound sets is advantageous overall for chemical development and persistence screening applications in an SSbD context.

### Implementation and Future Work

The 24-well plate biotransformation assay developed here provides an approach for high-throughput persistence screening that is in line with SSbD principles for early stage chemical assessment. Beyond this primary application, such tools also address regulatory needs, as this type of approach is expressly referenced in ECHA’s 2025 “Key Areas of Regulatory Challenge” publication as a way to support hazard and risk assessment under different regulations including REACH.^6^ Specifically, our assay represents a “middle ground” of persistence testing between current stringent screening methods and costly higher-tier simulation studies by reducing testing time from weeks to days while providing kinetic information about the compounds in question. While this work focuses on applications for chemical development, the assay can also be used to address the existing backlog of chemicals for which no degradation information exists.^34^ In addition, it can be used to generate large, high quality data sets for use in developing structure-based models to predict persistence, which are currently hindered by a lack of available data. Finally, while we focus on primary biotransformation consistent with regulatory simulation studies, the workflow’s well plate format and analytical capabilities position it for future adaptation to transformation product analysis through combinatorial pooling and MS2 spectra acquisition.

However, several areas require further development to achieve full standardization and broader applicability. First, we are currently applying our method to a set of approximately 200 compounds to validate its performance across a broader chemical space. This ongoing work will offer a larger data set of *k*_deg_ values and provide an opportunity to further verify some of the techniques for reducing analytical effort discussed here. Next, we acknowledge that compound measurability depends on complex physicochemical relationships beyond simple log *K*oc thresholds (SI Table S2.1 and accompanying discussion). While most specialty chemicals fall within our measurable scope, we are developing alternative approaches (full extraction, dilute inocula) for compounds outside this domain. Finally, to support standardization and interlaboratory comparison, a set of benchmark compounds needs to be defined that would allow biotransformation behavior of unknown compounds to be compared across different experiments and AS sources.

We are working with industry partners to demonstrate this complete workflow, validating its practical utility in chemical development settings. By shifting persistence assessment from a postdevelopment consideration to an integral part of the design phase, this work directly supports the fundamental SSbD principle of safer chemicals designed from the outset.

## Supplementary Material



## Notes

Information on test compounds and wastewater treatment plants, experimental details of biotransformation experiments and test compound analysis, area–time series for all test compounds, statistical information on variability in biotransformation rate constants, discussion of test compound introduction methodology, and discussion of how physicochemical properties relate to compound detectability (PDF)

## References

[1] Wang Z., Walker G. W., Muir D. C. G., Nagatani-Yoshida K. (2020). Toward a Global
Understanding of Chemical Pollution: A First Comprehensive Analysis
of National and Regional Chemical Inventories. Environ. Sci. Technol..

[2] Persson L., Carney Almroth B. M., Collins C. D., Cornell S., de Wit C. A., Diamond M. L., Fantke P., Hassellov M., MacLeod M., Ryberg M. W., Søgaard Jørgensen P., Villarrubia-Gomez P., Wang Z., Hauschild M. Z. (2022). Outside
the Safe Operating Space of the Planetary Boundary for Novel Entities. Environ. Sci. Technol..

[3] European environment — state and outlook 2020. European Environment Agency. https://www.eea.europa.eu/publications/soer-2020 (accessed 2024–08–03).

[4] Fenner K., Scheringer M. (2021). The Need for Chemical Simplification As a Logical Consequence
of Ever-Increasing Chemical Pollution. Environ.
Sci. Technol..

[5] Joint Research Centre (European Commission); Caldeira, C. ; Farcal, L. R. ; Garmendia Aguirre, I. ; Mancini, L. ; Tosches, D. ; Amelio, A. ; Rasmussen, K. ; Rauscher, H. ; Riego Sintes, J. ; Sala, S. Safe and Sustainable by Design Chemicals and Materials: Framework for the Definition of Criteria and Evaluation Procedure for Chemicals and Materials; Publications Office of the European Union: LU, 2022.

[6] De Coen, W. ECHA - Key Areas of Regulatory Challenge; ECHA-25-R-04-EN; European Chemicals Agency: Helsinki, Finland, 2025. https://echa.europa.eu/documents/10162/17228/key_areas_regulatory_challenge_2025_en.pdf/da33bf25-2b75-1fe9-c308-53043f9b9a28?t=1749466525527 (accessed 2025–07–19).

[7] van
Dijk J., Sharma A., Nowack B., Wang Z., Scheringer M. (2025). From Ambition
to Action: Navigating Obstacles and Opportunities of “Safe
and Sustainable by Design.”. Environ.
Sci. Technol..

[8] Cousins I. T., Ng C. A., Wang Z., Scheringer M. (2019). Why Is High
Persistence Alone a Major Cause of Concern?. Environ. Sci. Process. Impacts.

[9] Fenner K., Canonica S., Wackett L. P., Elsner M. (2013). Evaluating Pesticide
Degradation in the Environment: Blind Spots and Emerging Opportunities. Science.

[10] Kowalczyk A., Martin T. J., Price O. R., Snape J. R., van Egmond R. A., Finnegan C. J., Schäfer H., Davenport R. J., Bending G. D. (2015). Refinement of Biodegradation Tests
Methodologies and
the Proposed Utility of New Microbial Ecology Techniques. Ecotoxicol. Environ. Saf..

[11] Coll C., Fenner K., Screpanti C. (2023). Early Assessment
of Biodegradability
of Small Molecules to Support the Chemical Design in Agro & Pharma
R&D. CHIMIA.

[12] Test No. 301: Ready Biodegradability; Organisation for Economic Co-operation and Development: Paris, 1992.

[13] Test No. 307: Aerobic and Anaerobic Transformation in Soil; Organisation for Economic Co-operation and Development: Paris, 2002.

[14] Test No. 308: Aerobic and Anaerobic Transformation in Aquatic Sediment Systems; Organisation for Economic Co-operation and Development: Paris, 2002.

[15] Møller M. T., Birch H., Sjøholm K. K., Skjolding L. M., Xie H., Papazian S., Mayer P. (2024). Determining Marine Biodegradation
Kinetics of Chemicals Discharged from Offshore Oil Platforms—Whole
Mixture Testing at High Dilutions Increases Environmental Relevance. Environ. Sci. Technol..

[16] Li Z., McLachlan M. S. (2019). Biodegradation
of Chemicals in Unspiked Surface Waters
Downstream of Wastewater Treatment Plants. Environ.
Sci. Technol..

[17] Cregut M., Jouanneau S., Brillet F., Durand M.-J., Sweetlove C., Chenèble J.-C., L’Haridon J., Thouand G. (2014). High Throughput and
Miniaturised Systems for Biodegradability Assessments. Environ. Sci. Pollut. Res. Int..

[18] Francois B., Armand M., Marie-Jose D., Thouand G. (2016). From Laboratory to
Environmental Conditions: A New Approach for Chemical’s Biodegradability
Assessment. Environ. Sci. Pollut. Res..

[19] Martin T. J., Goodhead A. K., Acharya K., Head I. M., Snape J. R., Davenport R. J. (2017). High Throughput Biodegradation-Screening Test To Prioritize
and Evaluate Chemical Biodegradability. Environ.
Sci. Technol..

[20] Schäfer, R. ; Dönni, A. ; Thomann, M. ; Gulde, R. ; Eugster, F. ; Joss, A. ; Piazzoli, A. Entwicklung des AIA-Tests. Aqua & Gas, 2023. https://www.aquaetgas.ch/wasser/abwasser/20230329_ag4_entwicklung-des-aia-tests/ (accessed 2025–08–14).

[21] Fenner K., Screpanti C., Renold P., Rouchdi M., Vogler B., Rich S. (2020). Comparison of Small Molecule Biotransformation Half-Lives between
Activated Sludge and Soil: Opportunities for Read-Across?. Environ. Sci. Technol..

[22] Coll C., Screpanti C., Hafner J., Zhang K., Fenner K. (2025). Read-Across
of Biotransformation Potential between Activated Sludge and the Terrestrial
Environment: Toward Making It Practical and Plausible. Environ. Sci. Technol..

[23] Kalt M., Udressy C. I., Yu Y., Colliquet A., Fenner K. (2025). Preserving the Biotransformation
Potential of Activated
Sludge in Time: Toward Reproducible Incubation Experiments for Persistence
Assessment. Environ. Sci. Technol..

[24] Achermann S., Falås P., Joss A., Mansfeldt C. B., Men Y., Vogler B., Fenner K. (2018). Trends in Micropollutant Biotransformation
along a Solids Retention Time Gradient. Environ.
Sci. Technol..

[25] Joss A., Zabczynski S., Göbel A., Hoffmann B., Löffler D., McArdell C. S., Ternes T. A., Thomsen A., Siegrist H. (2006). Biological
Degradation of Pharmaceuticals in Municipal Wastewater Treatment:
Proposing a Classification Scheme. Water Res..

[26] Wick A., Fink G., Joss A., Siegrist H., Ternes T. A. (2009). Fate of
Beta Blockers and Psycho-Active Drugs in Conventional Wastewater Treatment. Water Res..

[27] Goodhead A. K., Head I. M., Snape J. R., Davenport R. J. (2014). Standard
Inocula Preparations Reduce the Bacterial Diversity and Reliability
of Regulatory Biodegradation Tests. Environ.
Sci. Pollut. Res..

[28] Martin T. J., Snape J. R., Bartram A., Robson A., Acharya K., Davenport R. J. (2017). Environmentally
Relevant Inoculum Concentrations Improve
the Reliability of Persistent Assessments in Biodegradation Screening
Tests. Environ. Sci. Technol..

[29] Davenport R., Curtis-Jackson P., Dalkmann P., Davies J., Fenner K., Hand L., McDonough K., Ott A., Ortega-Calvo J. J., Parsons J. R., Schäffer A., Sweetlove C., Trapp S., Wang N., Redman A. (2022). Scientific Concepts
and Methods for Moving Persistence Assessments into the 21st Century. Integr. Environ. Assess. Manag..

[30] Honti M., Fenner K. (2015). Deriving Persistence
Indicators from Regulatory Water-Sediment
Studies – Opportunities and Limitations in OECD 308 Data. Environ. Sci. Technol..

[31] Gulde R., Helbling D. E., Scheidegger A., Fenner K. (2014). pH-Dependent Biotransformation
of Ionizable Organic Micropollutants in Activated Sludge. Environ. Sci. Technol..

[32] Seller-Brison C., Brison A., Yu Y., Robinson S. L., Fenner K. (2024). Adaptation
towards Catabolic Biodegradation of Trace Organic Contaminants in
Activated Sludge. Water Res..

[33] McLachlan M. S., Zou H., Gouin T. (2017). Using Benchmarking
To Strengthen the Assessment of
Persistence. Environ. Sci. Technol..

[34] European Union Strategic Approach to Pharmaceuticals in the Environment; European Commission, 2019. https://eur-lex.europa.eu/legal-content/EN/TXT/PDF/?uri=CELEX:52019DC0128 (accessed 2025–09–25).

