# Supplementary material for: High-Throughput Miniaturized Biotransformation Testing Using Activated Sludge Enables Rapid Chemical Persistence Assessment
Source: Environ Sci Technol Lett. 2025 Oct 27;12(11):1561–6. doi: 10.1021/acs.estlett.5c00859 (PMC12613810; doi:10.1021/acs.estlett.5c00859)
Supplement: Supplementary file 1 [file ez5c00859_si_001.pdf]

# Supporting Information
# for
# High-throughput miniaturized biotransformation testing using activated sludge enables rapid chemical persistence assessment

Sarah B. Partanen*, Nicolas Mueller and Kathrin Fenner

**Author Information**

***Corresponding Author***

Sarah B. Partanen – Eawag, Swiss Federal Institute of Aquatic Science and Technology, 8600 Dubendorf, Switzerland; Department of Chemistry, University of Zurich, 8057 Zurich, Switzerland

Email: *sarah.partanen@eawag.ch*

***Authors***

Nicolas Mueller - Eawag, Swiss Federal Institute of Aquatic Science and Technology, 8600 Dubendorf, Switzerland

Kathrin Fenner – Eawag, Swiss Federal Institute of Aquatic Science and Technology, 8600 Dubendorf, Switzerland; Department of Chemistry, University of Zurich, 8057 Zurich, Switzerland

Number of pages: 46

Number of figures: 15

Number of tables: 5

# S1. Materials and Methods

## S1.1. Compound selection

**Table S1.1**. Pesticide and pharmaceutical compounds spiked into the activated sludge biotransformation experiments, their associated internal standard, and pertinent physico-chemical data. log *K*oc, log D, Henry's Law constants, and water solubility data were generally taken from the CompTox Chemicals Dashboard,[3] prioritizing experimental data when available, except for log *K*oc data for pesticides, which were taken from the University of Hertfordshire Pesticide Properties DataBase where available.[2]

| Compound name | Chemical class | CAS No. | Molecular Weight (g $mol^{-1}$) | log *K*oc | log D @ pH 7.4 | Henry's Law constant (Pa $m^3$ $mol^{-1}$) | Water solubility (g $L^{-1}$) | Associated internal standard |
|---|---|---|---|---|---|---|---|---|
| Albuterol*† | pharmaceutical | 18559-94-9 | 239.311 | 2.03 | -1.33 | $8.24 \times 10^{-6}$ | $1.44 \times 10^{+1}$ | NA |
| Atenolol | pharmaceutical | 29122-68-7 | 266.341 | 2.48 | -1.26 | $3.13 \times 10^{-5}$ | $1.41 \times 10^{+1}$ | Atenolol-d7 |
| Azoxystrobin | pesticide | 131860-33-8 | 403.388 | 2.77 | 2.50 | $7.40 \times 10^{-9}$ | $6.70 \times 10^{-3}$ | Azoxystrobin-d4 |
| Benzovindiflupyr | pesticide | 1072957-71-1 | 398.22 | 3.16‡ | 3.96 | $7.34 \times 10^{-6}$ | $9.44 \times 10^{-3}$ | Ritonavir-d6 |
| Bezafibrate† | pharmaceutical | 41859-67-0 | 361.820 | 4.14 | 0.734 | $2.27 \times 10^{-6}$ | $3.14 \times 10^{-2}$ | Bezafibrate-d4 |
| Bromoxynil* | pesticide | 1689-84-5 | 276.915 | 2.48 | 0.800 | $2.32 \times 10^{-2}$ | $1.20 \times 10^{-1}$ | NA |
| Carbamazepine | pharmaceutical | 298-46-4 | 236.274 | 2.74 | 1.2 | $2.27 \times 10^{-5}$ | $1.16 \times 10^{-1}$ | Carbamazepine-d8 |
| Clofibric acid*† | pharmaceutical metabolite | 882-09-7 | 214.650 | 1.94 | -0.837 | $1.68 \times 10^{-4}$ | $8.39 \times 10^{-1}$ | NA |
| Chlortoluron† | pesticide | 15545-48-9 | 212.676 | 2.02‡ | 2.44 | $1.64 \times 10^{-4}$ | $7.15 \times 10^{-2}$ | Chlortoluron-d6 |
| Cyantraniliprole | pesticide | 736994-63-1 | 473.72 | 2.38 | 2.35 | $7.01 \times 10^{-6}$ | $1.08 \times 10^{-3}$ | Atrazine-d5 |
| Cyclaniliprole | pesticide | 1031756-98-5 | 602.11 | 4.98‡ | 4.16 | $5.57 \times 10^{-6}$ | $7.22 \times 10^{-4}$ | Irgarol-d9 |
| Dicamba* | pesticide | 1918-00-9 | 221.03 | 1.50‡ | -1.16 | $2.54 \times 10^{-4}$ | $6.72 \times 10^{+1}$ | NA |
| Diuron* | pesticide | 330-54-1 | 233.094 | 2.83 | 2.71 | $1.53 \times 10^{-4}$ | $4.03 \times 10^{-2}$ | Diuron-d6 |
| Fenhexamid | pesticide | 126833-17-8 | 302.2 | 2.68 | 3.79 | $1.60 \times 10^{-5}$ | $2.00 \times 10^{-2}$ | Fenhexamid-d3 |
| Fenoxycarb | pesticide | 72490-01-8 | 301.342 | 3.00‡ | 4.09 | $4.94 \times 10^{-5}$ | $6.30 \times 10^{-3}$ | Terbuthylazine-d5 |
| Fipronil | pesticide | 120068-37-3 | 437.148 | 3.72‡ | 3.69 | $9.24 \times 10^{-4}$ | $2.85 \times 10^{-3}$ | Epoxiconazole-d4 |
| Florasulam | pesticide | 145701-23-1 | 359.28 | 1.34 | -0.952 | $1.68 \times 10^{-5}$ | $6.40 \times 10^{0}$ | Metoprolol-d7 |
| Fluconazole* | pharmaceutical | 86386-73-4 | 306.277 | 1.71 | 0.599 | $7.69 \times 10^{-4}$ | $4.84 \times 10^{0}$ | NA |
| Flufenamic acid† | pharmaceutical | 530-78-9 | 281.230 | 3.25 | 1.48 | $1.47 \times 10^{-4}$ | $5.68 \times 10^{-3}$ | Haloxyfop-d4 |
| Fluopyram | pesticide | 658066-35-4 | 396.72 | 3.56‡ | 4.15 | $1.72 \times 10^{-3}$ | $1.51 \times 10^{-2}$ | Fluopyram-d4 |
| Flupyradifurone | pesticide | 951659-40-8 | 288.678 | 1.99 | 1.46 | $6.85 \times 10^{-5}$ | $6.09 \times 10^{0}$ | Sulcotrione-d3 |

| | | | | | | | | |
|---|---|---|---|---|---|---|---|---|
| Flutianil | pesticide | 958647-10-4 | 426.45 | 3.28‡ | 4.48 | $1.84 \times 10^{-4}$ | $1.29 \times 10^{-4}$ | Clopidogrel-(+/-)-d4 |
| Imidacloprid | pesticide | 138261-41-3 | 255.661 | 1.87‡ | 0.473 | $1.94 \times 10^{-5}$ | $6.11 \times 10^{-1}$ | Imidacloprid-d4 |
| Isoproturon | pesticide | 34123-59-6 | 206.289 | 2.00‡ | 2.57 | $1.50 \times 10^{-4}$ | $6.50 \times 10^{-2}$ | Isoproturon-d6 |
| Ketoprofen*† | pharmaceutical | 22071-15-4 | 254.285 | 2.84 | $-3.8 \times 10^{-2}$ | $1.37 \times 10^{-4}$ | $9.87 \times 10^{-2}$ | NA |
| Kresoxim-methyl* | pesticide | 143390-89-0 | 313.353 | 2.97‡ | 3.43 | $1.98 \times 10^{-3}$ | $2.01 \times 10^{-3}$ | NA |
| Levetiracetam† | pharmaceutical | 102767-28-2 | 170.212 | 1.48 | -0.779 | $2.37 \times 10^{-4}$ | $3.68 \times 10^{+1}$ | Levetiracetam-d3 |
| Mandipropamid | pesticide | 374726-62-2 | 411.878 | 4.16‡ | 3.48 | $6.12 \times 10^{-7}$ | $4.20 \times 10^{-3}$ | Fluopyram-d4 |
| Mecoprop*† | pesticide | 93-65-2 | 214.650 | 1.67 | -0.260 | $1.68 \times 10^{-4}$ | $6.27 \times 10^{+1}$ | NA |
| Mefenamic acid† | pharmaceutical | 61-68-7 | 241.285 | 2.85 | 2.54 | $1.88 \times 10^{-5}$ | $2.53 \times 10^{-2}$ | Haloxyfop-d4 |
| Mesotrione | pesticide | 104206-82-8 | 339.32 | 2.09 | 0.522 | $1.03 \times 10^{-4}$ | $7.46 \times 10^{-1}$ | Mesotrione-d3 |
| Metaflumizone* | pesticide | 139968-49-3 | 506.408 | 4.49 | 5.85 | $5.08 \times 10^{-3}$ | $1.59 \times 10^{-5}$ | NA |
| Morphine*† | pharmaceutical | 57-27-2 | 285.343 | 5.32 | $-1.8 \times 10^{-2}$ | $7.69 \times 10^{-6}$ | $1.49 \times 10^{-1}$ | NA |
| Oxathiapiprolin | pesticide | 1003318-67-9 | 539.53 | 3.99 | 4.37 | $5.44 \times 10^{-3}$ | $1.73 \times 10^{-4}$ | Epoxiconazole-d4 |
| Pargyline*† | pharmaceutical | 306-07-0 | 159.232 | 2.10 | 2.39 | $4.03 \times 10^{+1}$ | $5.05 \times 10^{0}$ | NA |
| Pravastatin*† | pharmaceutical | 81093-37-0 | 424.534 | 3.19 | 0.697 | $1.18 \times 10^{-6}$ | $8.32 \times 10^{-2}$ | NA |
| Primidone† | pharmaceutical | 125-33-7 | 218.256 | 1.90 | 0.734 | $4.33 \times 10^{-5}$ | $5.00 \times 10^{-1}$ | Primidone-d5 |
| Propachlor† | pesticide | 1918-16-7 | 211.690 | 1.90 | 2.23 | $1.25 \times 10^{-2}$ | $6.77 \times 10^{-1}$ | Flufenacet-d4 |
| Proquinazid | pesticide | 189278-12-4 | 372.206 | 2.81‡ | 3.16 | $1.64 \times 10^{-3}$ | $6.85 \times 10^{-2}$ | Triclocarban-13C6 |
| Pseudoephedrine*† | pharmaceutical | 90-82-4 | 165.236 | 2.11 | -0.721 | $1.16 \times 10^{-2}$ | $4.58 \times 10^{+1}$ | NA |
| Quetiapine† | pharmaceutical | 111974-69-7 | 383.510 | 3.05 | 2.29 | $1.72 \times 10^{-5}$ | $5.10 \times 10^{-2}$ | Sulfadimethoxin-d4 |
| Sulfamethoxazole | pharmaceutical | 723-46-6 | 253.280 | 1.96 | -0.494 | $1.76 \times 10^{-5}$ | $5.37 \times 10^{-1}$ | Sulfamethoxazole-d4 |
| Sulfathiazole† | pharmaceutical | 72-14-0 | 255.310 | 2.43 | -0.362 | $4.12 \times 10^{-6}$ | $7.28 \times 10^{-1}$ | Sulfathiazol-d4 |
| Terbuthylazine | pesticide | 5915-41-3 | 229.71 | 2.32‡ | 3.10 | $1.25 \times 10^{-3}$ | $8.09 \times 10^{-3}$ | Terbuthylazine-d5 |
| Topramezone | pesticide | 210631-68-8 | 363.39 | 2.23 | 0.701 | $6.39 \times 10^{-5}$ | $9.99 \times 10^{+1}$ | Primidone-d5 |
| Valsartan† | pharmaceutical | 137862-53-4 | 435.528 | 2.77 | -2.26 | $7.86 \times 10^{-5}$ | $2.03 \times 10^{-2}$ | Valsartan-13C5-15N |

*Compounds that are analyzed in negative mode only, or compounds that were not detected with the instrument method employed

†Compounds added to experiments after Exp3 (See Table S1.3)

‡log *K*oc values taken from the CompTox Chemicals Dashboard

## S1.2. Wastewater treatment plant site selection

**Table S1.2.** Summary of WWTPs and their physico-chemical parameters measured from activated sludge samples taken on September 16th, 2024.

| ***WWTP name*** | | | | | ***Neugut*** | ***Egg-Oetwil*** | ***Winterthur*** | ***Birmensdorf*** | ***Werdhoelzli*** | ***Bern**** |
|---|---|---|---|---|---|---|---|---|---|---|
| | | | | | | | | | | |
| ***Parameter*** | *Detail* | *Units* | *Detection limit* | | | | | | | |
| **Connected inhabitants** | | | | | 36,232 | 11,707 | 112,327 | 21,437 | 394,812 | 115,200 |
| **Daily flow** | | $m^3$/day | | | 22,000 | 4,650 | 57,600 | 11,500 | 207,100 | 190,446 |
| **Conductivity** | | µS/cm 20°C | | | 1024 | 1075 | 1199 | 894 | 620 | |
| **pH** | | - | | | 7.91 | 7.88 | 7.96 | 7.85 | 7.84 | |
| **Alkalinity** | | mmol/L | 0.2 | | 5.21 | 5.35 | 3.85 | 3.91 | 3.29 | |
| **Total hardness** | | mmol/L | 0.2 | | 3.16 | 3.36 | 3.30 | 2.63 | 2.14 | |
| **Sodium** | $Na^+$ | mg/L | 1.0 | | 100.1 | 108.9 | 124.3 | 90.4 | 48.5 | |
| **Magnesium** | $Mg^{2+}$ | mg/L | 1.0 | | 17.3 | 19.4 | 20.7 | 14.1 | 12.3 | |
| **Calcium** | $Ca^{2+}$ | mg/L | 5.0 | | 92.3 | 97.2 | 92.3 | 77.4 | 61.6 | |
| **Phosphorus** | $K^+$ | mg/L | 0.5 | | 12.3 | 21.3 | 14.7 | 12.2 | 14.0 | |
| **Fluoride** | $F^-$ | mg/L | 0.05 | | 0.08 | 0.22 | 0.06 | 0.10 | 0.07 | |
| **Chloride** | $Cl^-$ | mg/L | 0.5 | | 151.2 | 134.9 | 205.3 | 138.7 | 56.1 | |
| **Bromide** | $Br^-$ | mg/L | 0.05 | | <0.05 | 0.51 | 1.02 | <0.05 | <0.05 | |
| **Nitrate** | $NO_3^-$-N | mg/L | 0.1 | | 12.3 | 6.1 | 22.4 | 11.0 | 10.5 | |
| **Sulfate** | $SO_4^{2-}$ | mg/L | 1 | | 37 | 123 | 59 | 50 | 55 | |
| **Ammonium** | $NH_4^+$-N | µg/L | 5.0 | | 9.4 | 165.1 | 163.1 | 145.3 | 380.0 | |

| | | | | | | | | | | |
|---|---|---|---|---|---|---|---|---|---|---|
| **Nitrite** | $NO_2^-$-N | µg/L | 1.0 | | 3.0 | 47.0 | 38.0 | 77.0 | 49.0 | |
| **Phosphate (o-P)** | $PO_4^{3-}$-P | µg/L | 1.0 | | 422.5 | 298.0 | 252.0 | 250.5 | 355.5 | |
| **Dissolved Phosphorus (D-P)** | P | mg/L | 0.003 | | 0.453 | 0.319 | 0.281 | 0.264 | 0.381 | |
| **Dissolved organic carbon (DOC)** | C | mg/L | 0.5 | | 4.95 | 4.59 | 4.90 | 4.05 | 5.10 | |
| **Total organic carbon (TOC)** | C | mg/L | 0.5 | | 53.3 | 112.0 | 53.5 | 99.7 | 162.0 | 717.0 |
| **Total suspended solids (TSS)** | | g/L | | | 2.6 | 4.8 | 3.4 | 3.9 | 3.7 | 16.2 |

*Sludge from Bern WWTP was produced using a biofilter technology called Biostyr rather than through a conventional AS process. The sludge collected was therefore quite different from that of the other WWTPs including being very difficult to filter. As such, physico-chemical data from the Bern sludge was not collected.

## S1.3. Activated sludge biotransformation experiments

***AS collection***

Activated sludge was sampled from the aeration basin of the municipal wastewater treatment plants whenever possible. A grab sample of between 500 mL and 2 L was taken the day before each experiment and transported back to the lab. Transit time was usually less than 15 minutes, except for the multi-AS experiment, where samples were chilled for up to 4 hours before arriving at the lab.

***AS pre-treatment***

Upon arrival at the lab, the AS was pre-treated to homogenize the flocs. The homogenization process consisted of centrifuging 50 mL aliquots of AS for 10 minutes at 4000 rpm and 20°C, then re-suspending the flocs by shaking vigorously. Homogenized AS was mixed on an orbital shaker at 150 rpm and 20°C overnight until the experiment was initiated (< 24 hr).

***AS controls***

Sorption and abiotic controls were run alongside many biotransformation experiments (see Table S1.3 for details). Sorption controls consisted of homogenized AS autoclaved for 20 minutes at 121 °C and 2 bar. It served to examine the sorption of the compounds to the inactivated biomass and floc matrix. Abiotic controls consisted of autoclaved AS supernatant and were used to gauge whether abiotic removal processes were occurring. Since the abiotic degradation behaviour of the test compounds had previously been examined, abiotic controls were not included in every experiment.

Large-volume controls consisting of 50mL AS in 100 mL Schott bottles were also included in each experiment to examine the impact of scale on biotransformation rate constant.

***Experiment initiation***

A mixture of all benchmark compounds in ethanol was spiked to each experimental well or 50 mL reactor to reach an initial test compound concentration of 6 µg/L each. The ethanol was evaporated off for 15 minutes to limit the addition of dissolved organic carbon to the test system, and the compounds were re-dissolved in a small amount of AS supernatant for 30 minutes before addition of homogenized AS to initiate the experiment. Well plates were covered with BreatheEasy film and large volume controls

were capped with lids containing two holes to facilitate gas exchange while limiting evaporation.

Well plates were incubated for 48 hours either in an orbital shaker (150 rpm and 20°C) or a plate shaker (variable rpm required for floc suspension, ambient temperature (20-25°C)). 50 mL controls were incubated in the orbital shaker.

***Sampling***

At 7 time points during each experiment aliquots were taken for chemical analysis. 200 µL of sample were taken from each well or 50 mL control and centrifuged (4000 rpm, 4°C) to separate the flocs from the supernatant. 100 µL of AS supernatant were pipetted into a glass-lined 96 well plate for analysis and spiked with 10 µL of internal standard solution (see Table S1.1) for a final internal standard concentration of 1 µg/L. As the analysis 96-well plates were filled over the course of the 48-hour experiment, in between sampling, the plates were covered with non-permeable film to prevent evaporation.

**Table S1.3.** Summary of experimental conditions for all experiments used in the development of the miniaturized biotransformation test. Data from experiments 4-7 are shown in Figure 1 in the main text. Experiments 1-3 were preliminary method development trials and are not included in the main figure analysis.

| | Date of sludge sampling | Spiked compounds | Source of activated sludge | Duration | Scales | Mixing | Included controls | Replicates (n) | Comments |
|---|---|---|---|---|---|---|---|---|---|
| **Exp1** | 20.06.2023 | Table S1.1, none labeled † | Neugut WWTP, Dübendorf | 48 hours; sorption control 24 hours | -2 mL AS in 5 mL vial<br>-96 well plate | Orbital shaker | -Large volume<br>-Sorption | Large vol.: 2<br>5 mL vial: 4<br>96 wp: 3 | -Compounds tested in sub-mixes of 10, as well as a mixture of all compounds<br>-Sludge not homogenized<br>-Plastic 96 well plate |
| **Exp2** | 25.07.2023 | Table S1.1, none labeled † | Neugut WWTP, Dübendorf | 48 hours; sorption control 24 hours | -96 well plate | Orbital shaker | -Large volume<br>-Sorption | Large vol.: 2<br>96 wp: 3 | -Compounds tested in sub-mixes of 10, as well as a mixture of all compounds<br>-Different sludge homogenization methods tested<br>-Glass 96 well plates used from now on |
| **Exp3** | 19.09.2023 | Table S1.1, none labeled † | Neugut WWTP, Dübendorf | 48 hours; sorption and abiotic controls 24 hours | -1 mL AS in 2 mL vial<br>-96 well plate | Orbital shaker | -Large volume<br>-Sorption<br>-Abiotic | Large vol.: 2<br>2 mL vial: 3<br>96 wp: 3 | -Mixture of all compounds used from now on<br>-Sludge homogenized by centrifuging and shaking from now on |
| **Exp4** | 09.01.2024 | Table S1.1 | Neugut WWTP, Dübendorf | 48 hours; sorption control 24 hours | -24 well plate<br>-48 well plate<br>-96 well plate<br>-96 deep well plate | -Orbital shaker<br>-Plate shaker | -Large volume<br>-Sorption | Large vol.: 2<br>24 wp: 6<br>48 wp: 4<br>96 wp: 8<br>96 deep wp: 8 | -Instrument autosamples from glass-lined 96 well plate instead of HPLC vials from now on |

| | | | | | | | | | |
|---|---|---|---|---|---|---|---|---|---|
| **Exp5** | 12.03.2024,<br>19.03.2024 | Table S1.1 | Neugut WWTP, Dübendorf | 48 hours; sorption and abiotic controls 24 hours | -24 well plate<br>-48 well plate | -Orbital shaker (only large volume)<br>-Plate shaker | -Large volume<br>-Sorption<br>-Abiotic | Large vol.: 2<br>24 wp: 12<br>48 wp: 8 | |
| **Exp6** | 16.04.2024,<br>23.04.2024 | Table S1.1 | Neugut WWTP, Dübendorf | 48 hours; sorption and abiotic controls 24 hours | -24 well plate | -Orbital shaker<br>-Plate shaker | -Large volume<br>-Sorption<br>-Abiotic | Large vol.: 2<br>24 wp: 6 | -Includes a biotransformation experiment where only 4 timepoints were taken |
| **Exp7** | 16.09.2024 | Table S1.1 | See Table S1.2 | 48 hours; sorption control 24 hours | -24 well plate | Orbital shaker | -Large volume<br>-Sorption | Large vol.: 2<br>24 wp: 3 | |

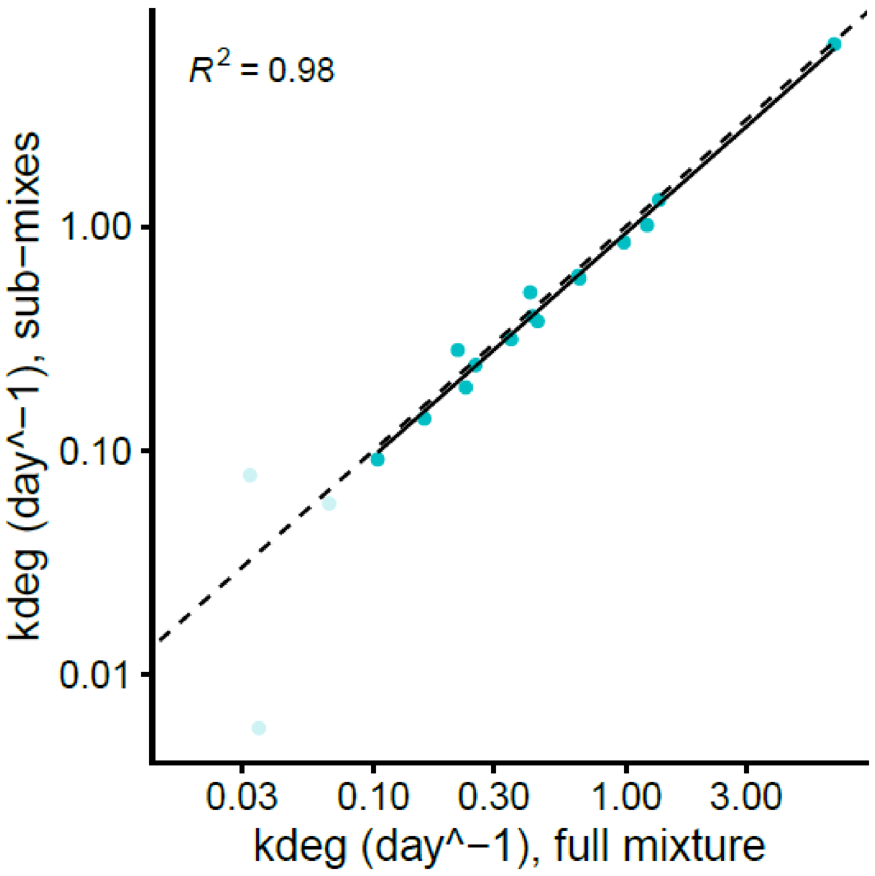


**Figure S1.1.** Correlation between $k_{deg}$ values in a 96 well plate where all test compounds were combined (x-axis) with those where sub-mixes of 10 compounds were tested. The dashed line is the 1:1 line, and the solid line is the line of best fit. Pale points represent $k_{deg}$ values smaller than $k_{deg,min}$ (see section S1.4 for definition).

## S1.4. Test compound analysis

***Justification of analytical approach***

Liquid chromatography coupled to high-resolution mass spectrometry (LC-HRMS) was selected to enable direct quantification of parent compound disappearance at environmentally relevant concentrations (µg/L), providing essential kinetic information for persistence assessment outside the pass/fail outcomes associated with ready biodegradability tests. While LC-HRMS requires specialized instrumentation, companies engaged in chemical R&D typically possess such analytical capabilities for compound characterization, and contract laboratories routinely offer these services for those without in-house facilities.

***Instrument method***

Test compounds were analyzed using a Thermo Scientific UltiMate 3000 UHPLC coupled to a Thermo Scientific Q Exactive Hybrid Quadrupole-Orbitrap mass spectrometer

(Thermo Fisher Scientific). 30 μL of sample was injected into the system, where the mobile phase consisted of ultrapure water and methanol, both supplemented with 0.1% formic acid. The eluate was injected to a XBridge BEH C18 column equipped with a pre-column (column: 2.1 x 50 mm, 3.5 μm particle size; pre-column: 2.1 x 5 mm, 3.5 μm particle size, both supplied by Waters) under gradient conditions (6 minutes total, see Table S1.4) at a flow rate of 0.2 mL/min. Electrospray ionization (ESI) was used as the ionization method, with a spray voltage of 3.00 kV, a capillary temperature of 350°C, sheath gas flow rate of 5, aux gas flow rate of 3 (flow rates in arbitrary units), and S-lens RF level of 50.0. Full scan mass spectra were obtained in positive mode in the range of 100 – 1,000 m/z at a resolution of 70,000. Data dependent MS2 spectra were recorded at a resolution of 17,500 using a Top-5 approach.

**Table S1.4.** Gradient conditions of the mobile phase for liquid chromatography portion of test compound analysis (constant flow rate of 0.2 mL/min).

| Time (min) | 0 | 0.5 | 4 | 5 | 5 | 6 |
|---|---|---|---|---|---|---|
| Water % | 95 | 95 | 5 | 5 | 95 | 95 |
| Methanol % | 5 | 5 | 95 | 95 | 5 | 5 |

Note that our instrument method continues to evolve. The current version of our method is 8 minutes long, but full mass spectra are acquired in positive/negative switching mode allowing for the quantification of a wider range of test compounds.

***Half-life derivation***

The use of low initial concentrations of compounds (6 μg/L) in the biotransformation experiments means that we expect to observe first-order kinetics in the system.[4] Biotransformation rate constants can hence be derived from the rate law for a first-order reaction, shown in equation 1.

$$\frac{dA}{dt} = -k_{deg}A \qquad \text{S1}$$

where A is the peak area or concentration of compound A (μg/L), and $k_{deg}$ is the biotransformation rate constant ($day^{-1}$).

Biotransformation half-lives can be calculated from $k_{deg}$ values using equation 2.

$$t_{1/2} = \frac{\ln(2)}{k_{deg}} \qquad \text{S2}$$

where $t_{1/2}$ is the biotransformation half-life (day).

$k_{deg}$ values were obtained from global linear regressions of replicate time series data, where the slope parameter is $k_{deg}$. Error bars in Figure 1 in the main text therefore represent the standard error of the slope parameter of the regression across replicate experiments.

***Method for reducing analytical effort***

The first-order rate law can be written in a linearized form of the integrated rate law (equation S3):

$$ln\left(\frac{A}{A_0}\right) = -\mathrm{k}_{deg}\mathrm{t} \qquad \text{S3}$$

where $A_0$ is the initial peak area or concentration of compound A (μg/L).

Normally rate constants are derived by plotting $ln\left(\frac{A}{A_0}\right)$ vs. time and taking the slope of the line as $k_{deg}$. However, initial and final peak areas or concentrations of A and the duration of the experiment can also be used directly in equation 3 to derive $k_{deg}$ values. This means that rather than an entire time series, potentially only initial and final peak areas or concentrations (i.e., removal over the experimental time period) must be measured to get an estimate of the biotransformation behaviour of a compound.

***Definition of measurable $k_{deg}$ values***

We defined the smallest $k_{deg}$ value measurable by our experimental procedure as less than 10% compound degradation over the course of the 48-hour experiment. Therefore, using equation S3:

$$k_{deg,min} = \frac{-ln(1-0.1)}{2}$$

where $k_{deg,min}$ is the smallest measurable $k_{deg}$ value (days).

## S2. Results and Discussion

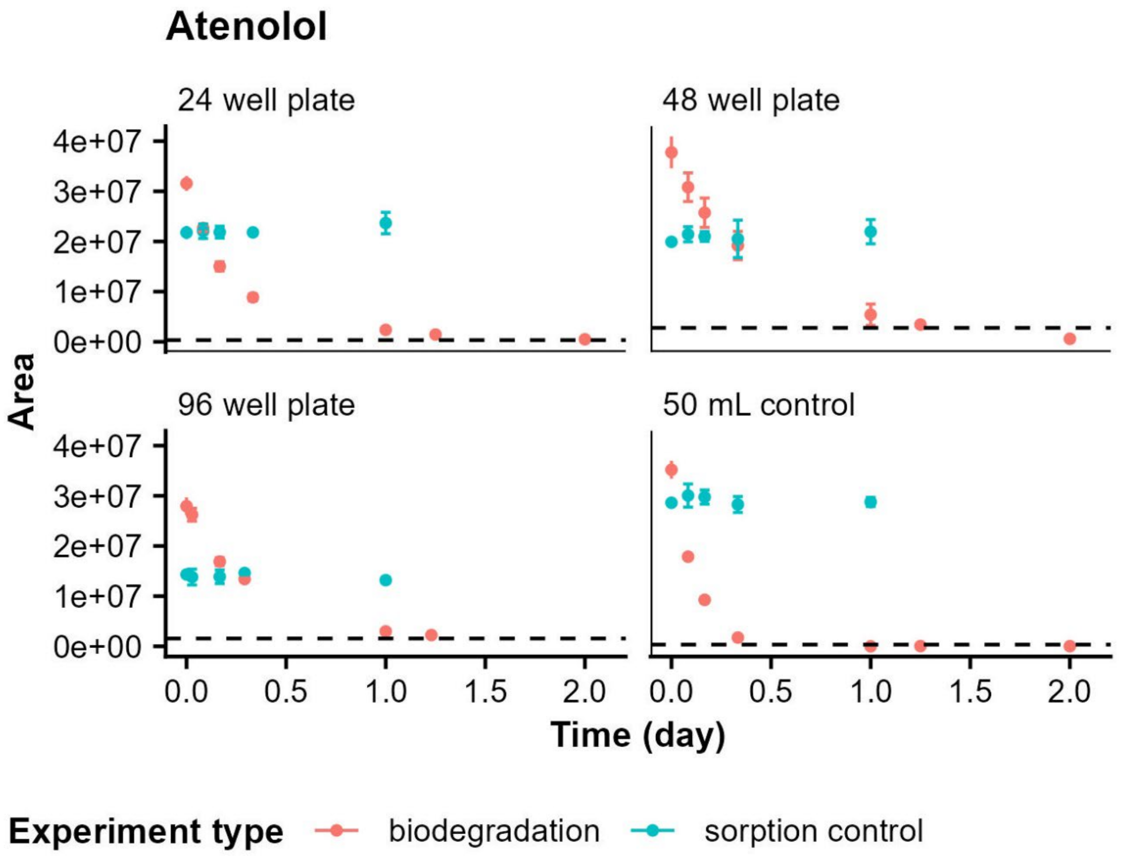


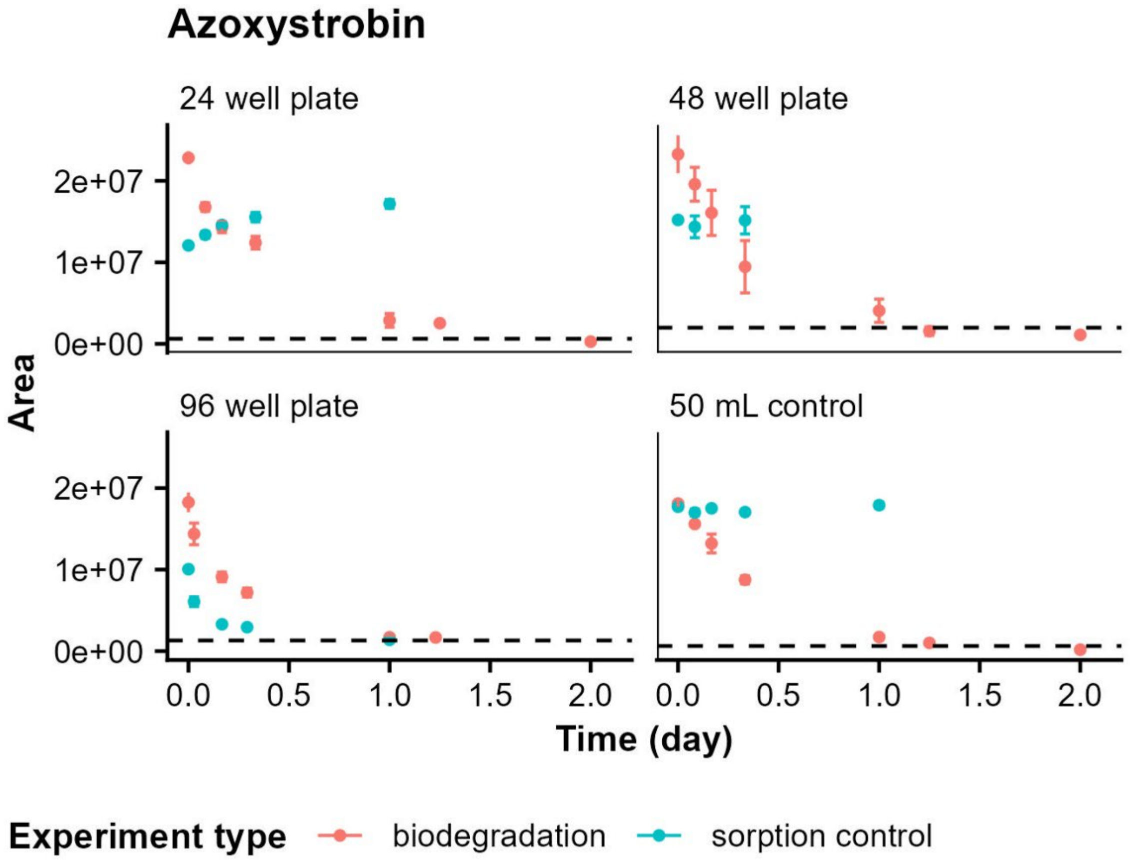

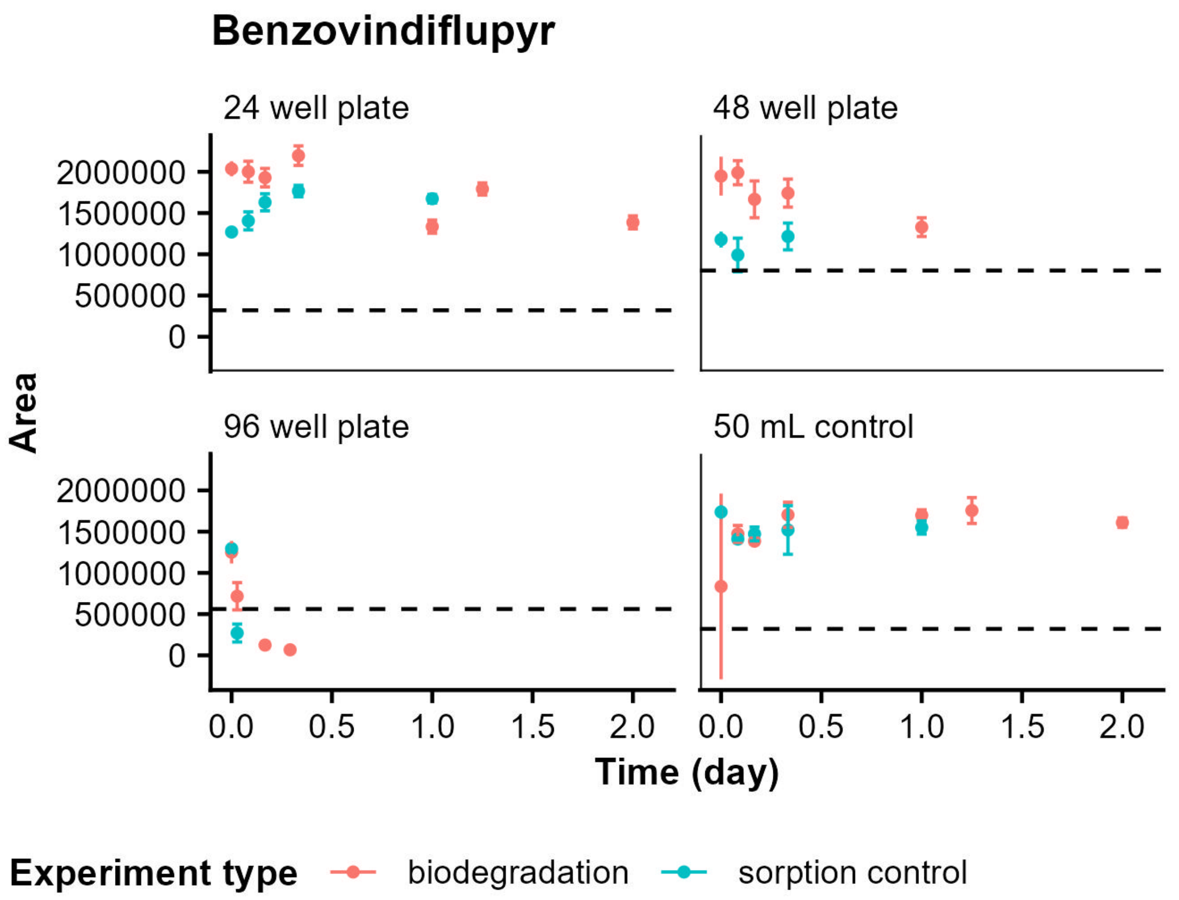
Benzovindiflupyr
24 well plate
48 well plate
96 well plate
50 mL control
2000000
1500000
1000000
500000
0
Area
0.0
0.5
1.0
1.5
2.0
Time (day)
Experiment type
biodegradation
sorption control

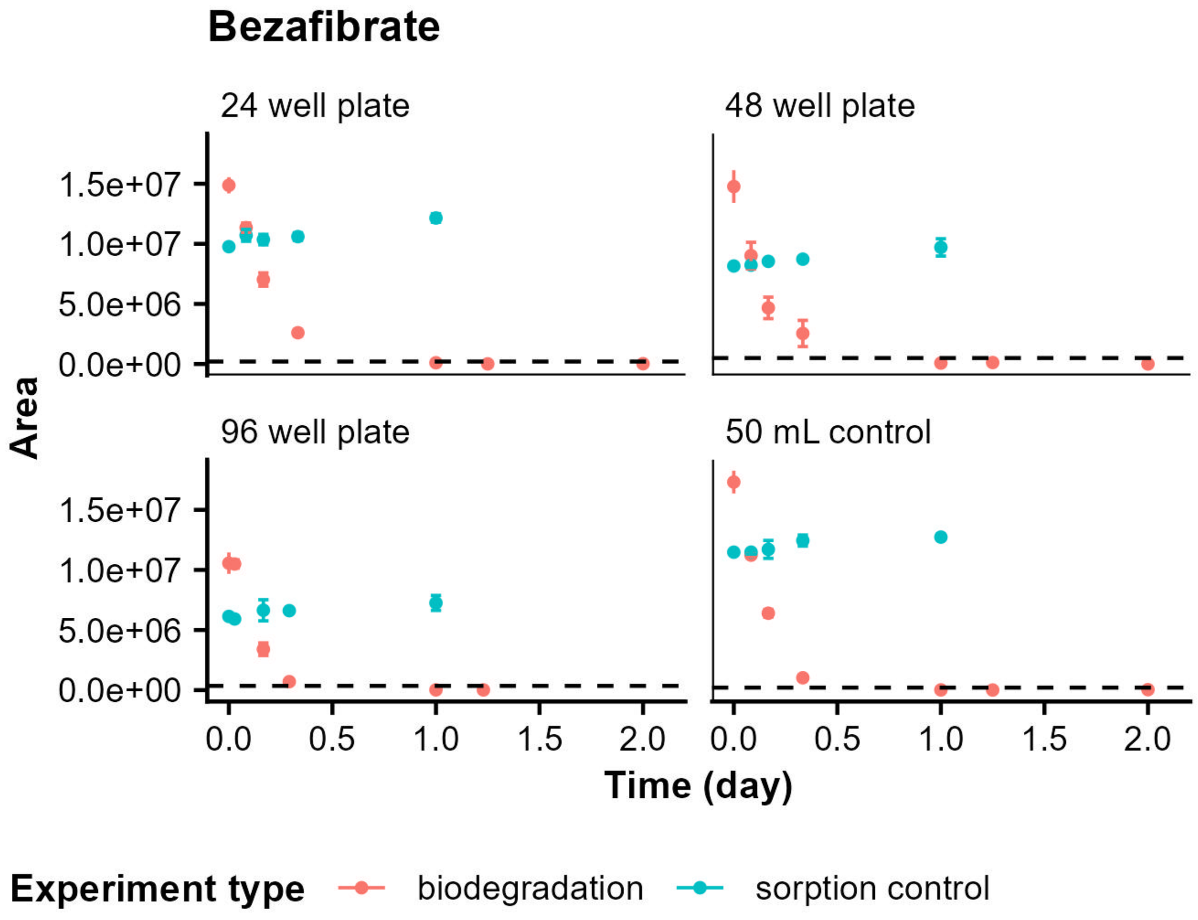
Bezafibrate
24 well plate
48 well plate
96 well plate
50 mL control
1.5e+07
1.0e+07
5.0e+06
0.0e+00
Area
0.0
0.5
1.0
1.5
2.0
Time (day)
Experiment type
biodegradation
sorption control

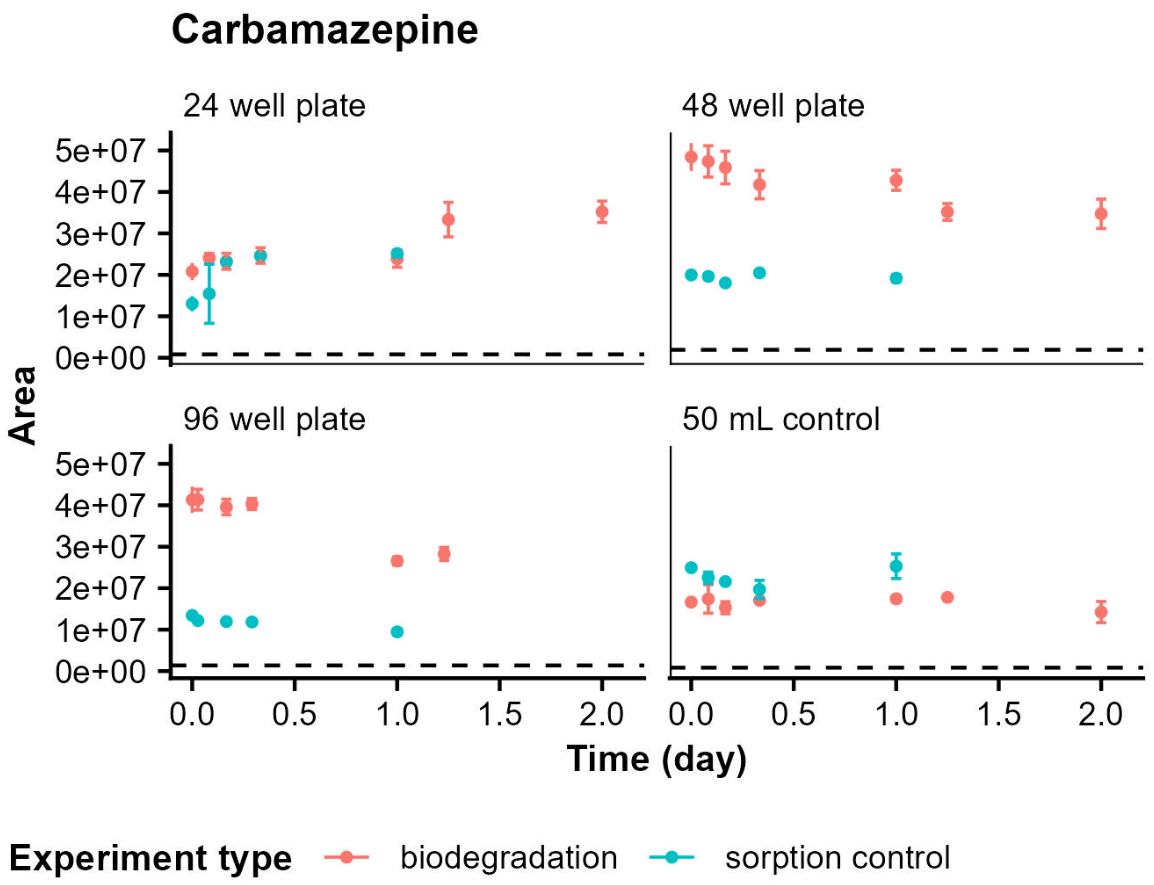
Carbamazepine
24 well plate
48 well plate
96 well plate
50 mL control
Area
5e+07
4e+07
3e+07
2e+07
1e+07
0e+00
0.0
0.5
1.0
1.5
2.0
Time (day)
Experiment type
biodegradation
sorption control

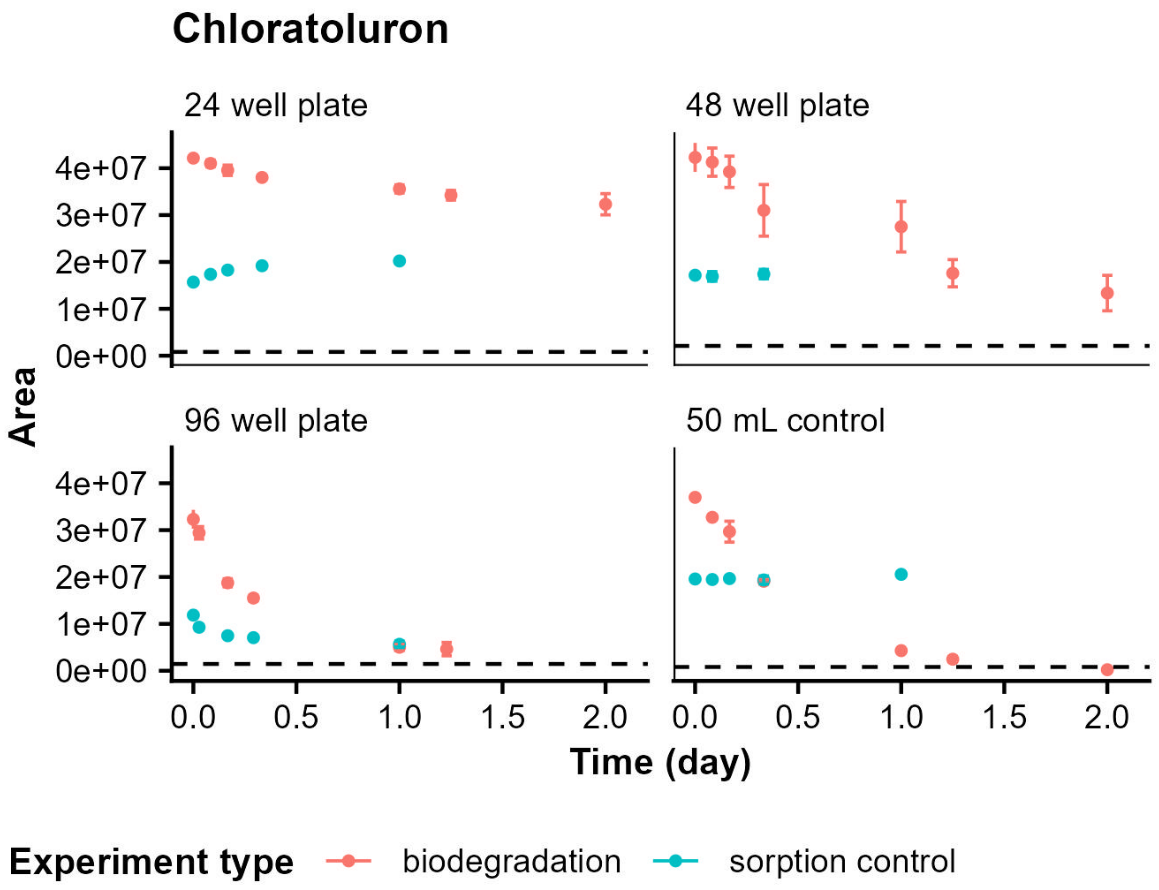
Chloratoluron
24 well plate
48 well plate
96 well plate
50 mL control
Area
4e+07
3e+07
2e+07
1e+07
0e+00
0.0
0.5
1.0
1.5
2.0
Time (day)
Experiment type
biodegradation
sorption control

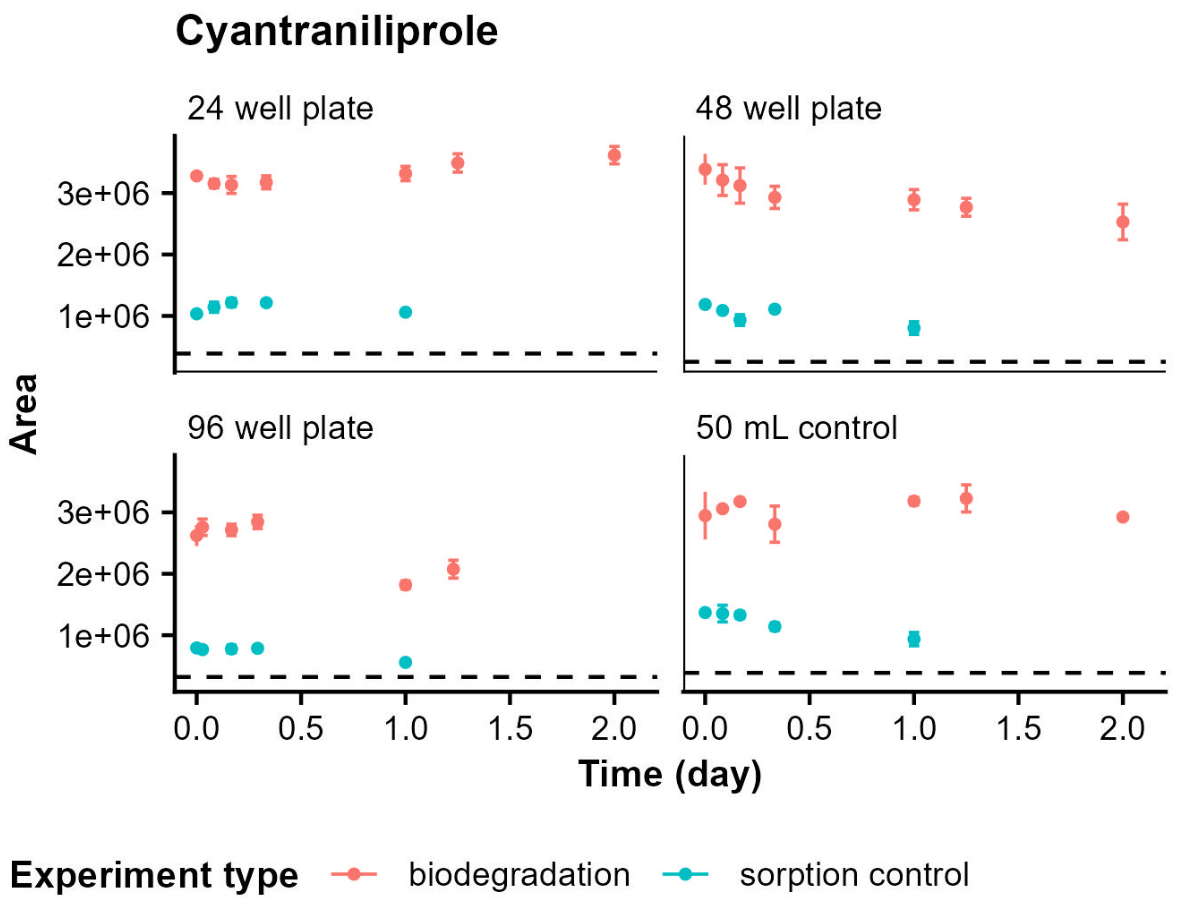

Cyantraniliprole
24 well plate
48 well plate
96 well plate
50 mL control
Area
3e+06
2e+06
1e+06
0.0
0.5
1.0
1.5
2.0
Time (day)
Experiment type
biodegradation
sorption control


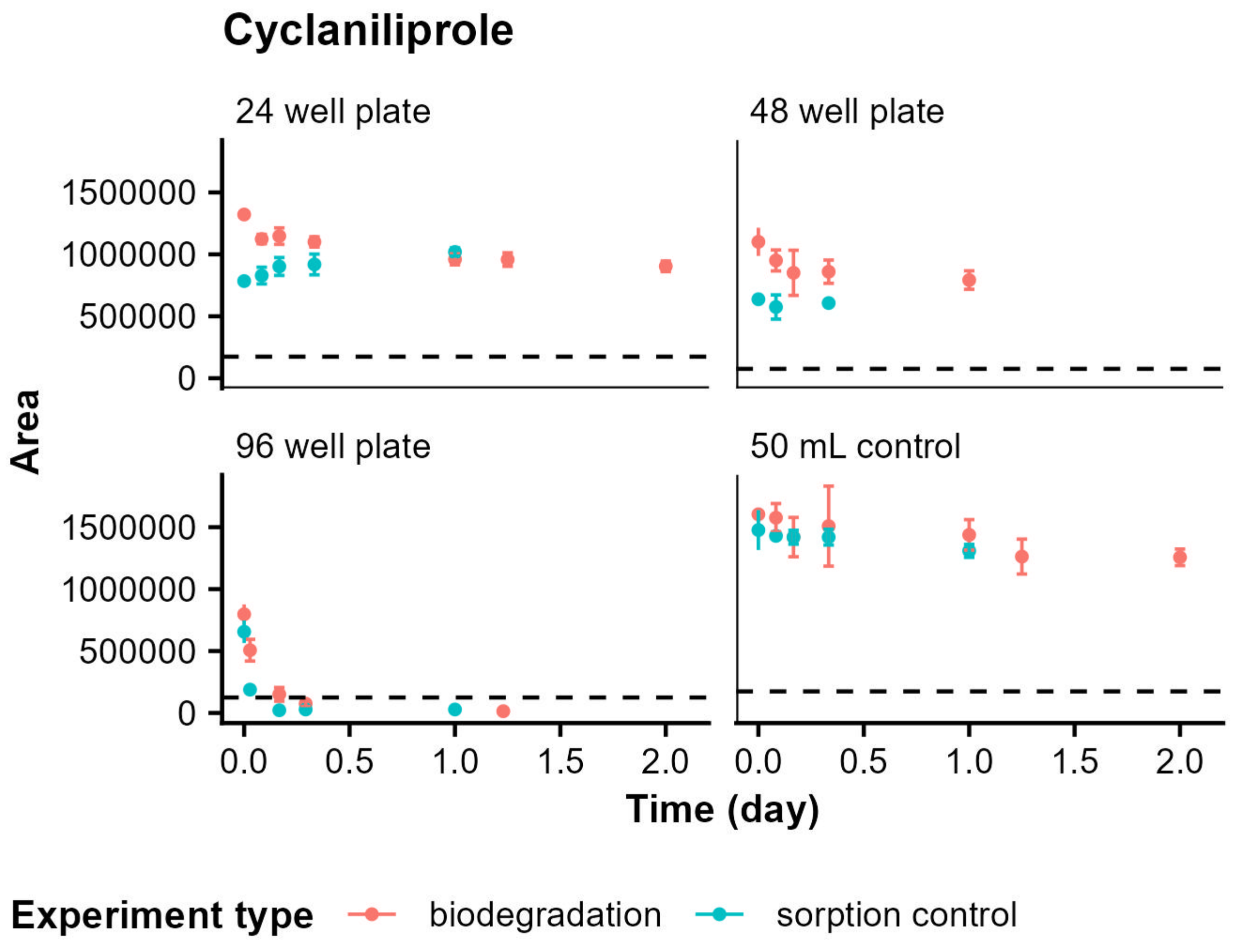

Cyclaniliprole
24 well plate
48 well plate
96 well plate
50 mL control
Area
1500000
1000000
500000
0
0.0
0.5
1.0
1.5
2.0
Time (day)
Experiment type
biodegradation
sorption control

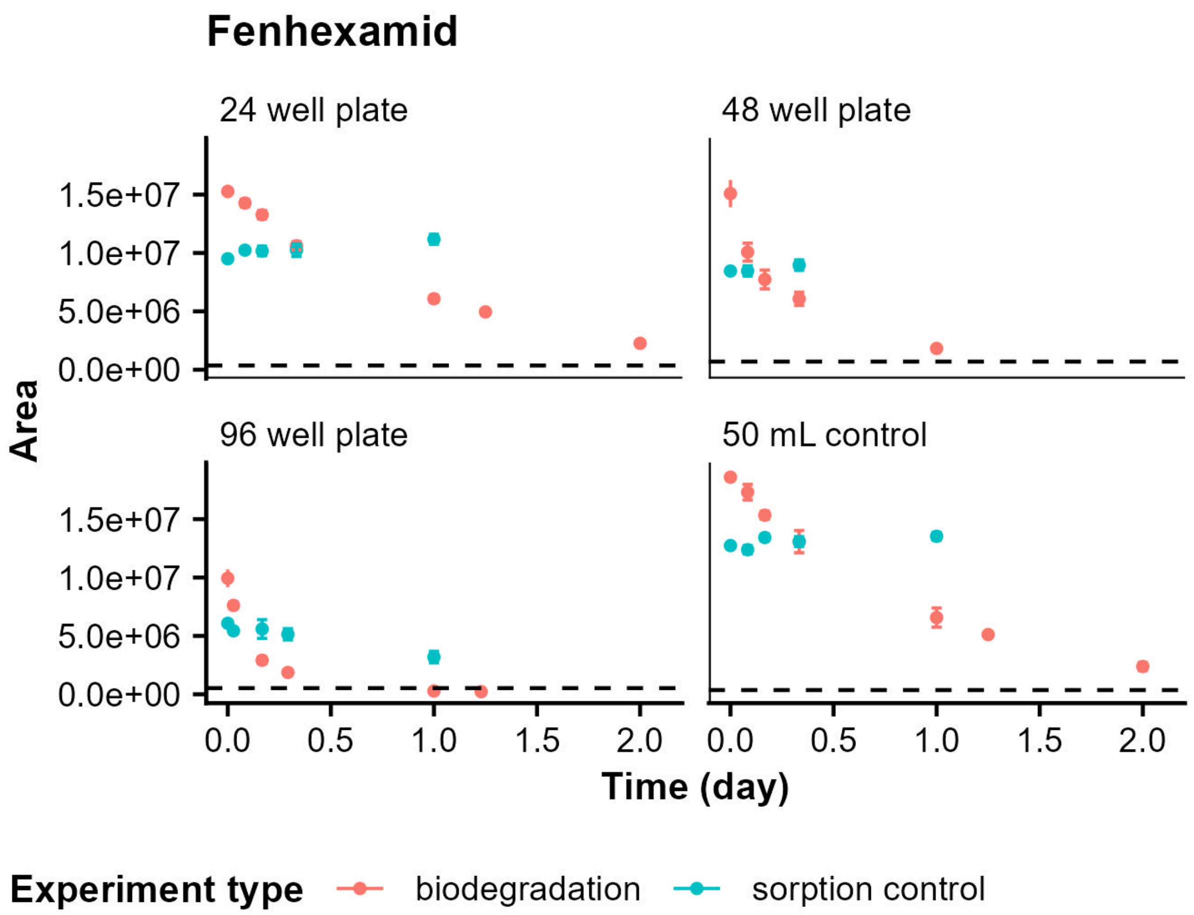
Fenhexamid
24 well plate
48 well plate
96 well plate
50 mL control
1.5e+07
1.0e+07
5.0e+06
0.0e+00
Area
0.0
0.5
1.0
1.5
2.0
Time (day)
Experiment type
biodegradation
sorption control

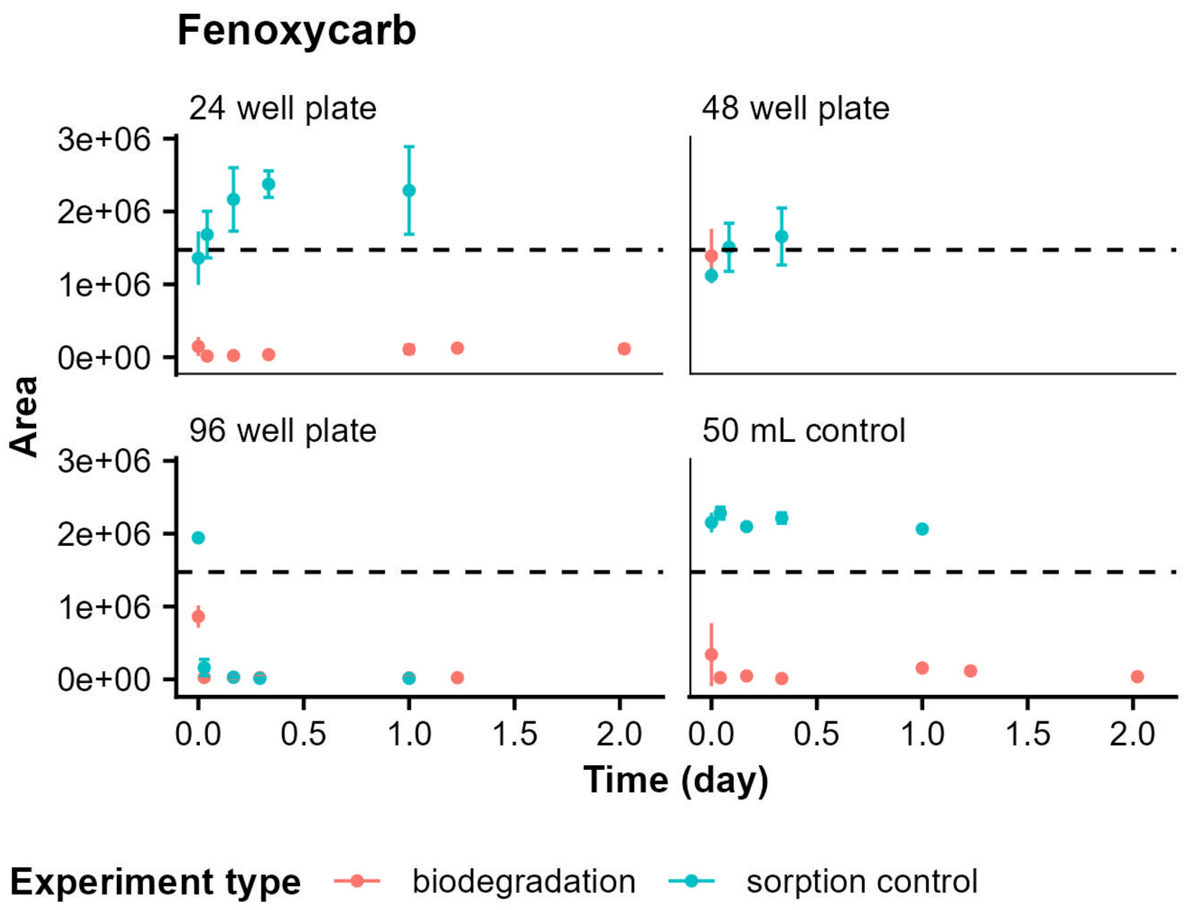
Fenoxycarb
24 well plate
48 well plate
96 well plate
50 mL control
3e+06
2e+06
1e+06
0e+00
Area
0.0
0.5
1.0
1.5
2.0
Time (day)
Experiment type
biodegradation
sorption control

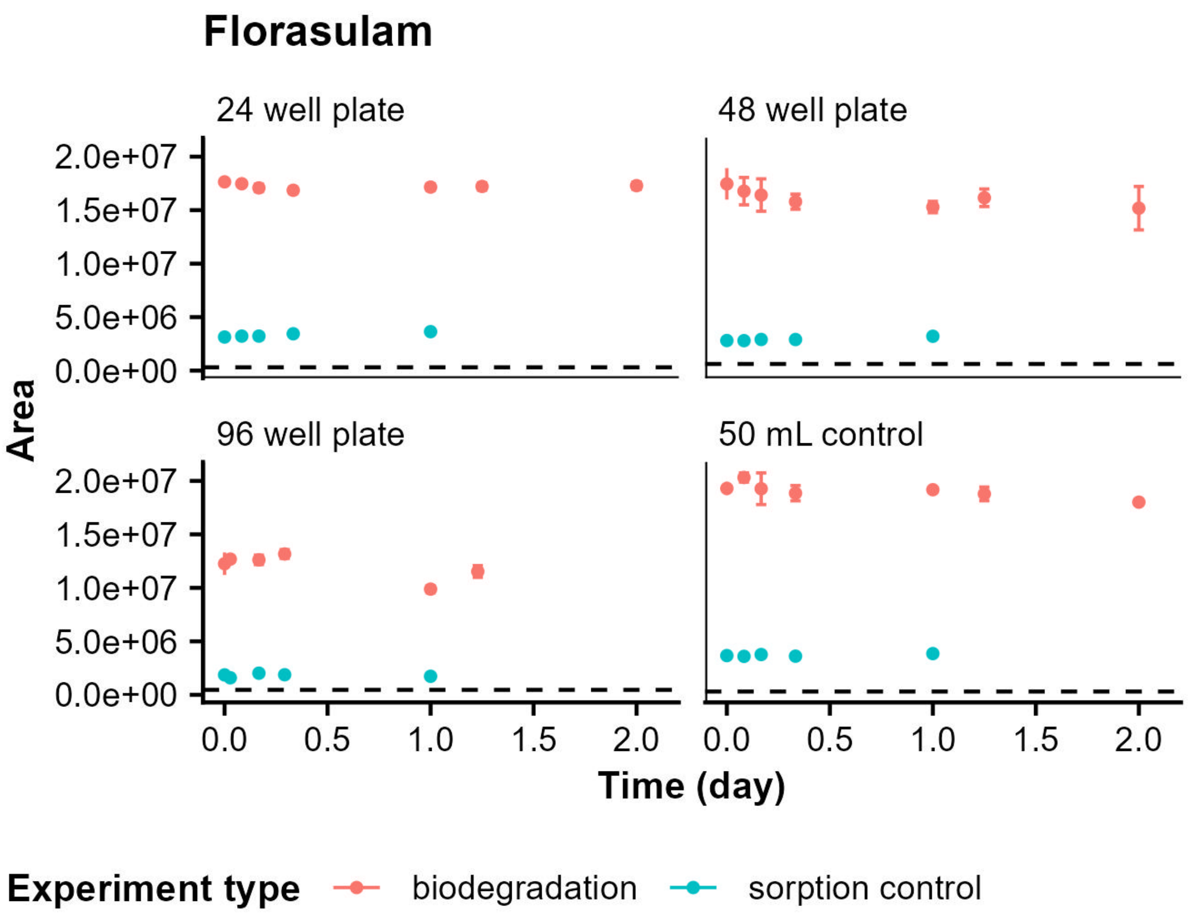
Florasulam
24 well plate
48 well plate
96 well plate
50 mL control
2.0e+07
1.5e+07
1.0e+07
5.0e+06
0.0e+00
Area
0.0
0.5
1.0
1.5
2.0
Time (day)
Experiment type
biodegradation
sorption control

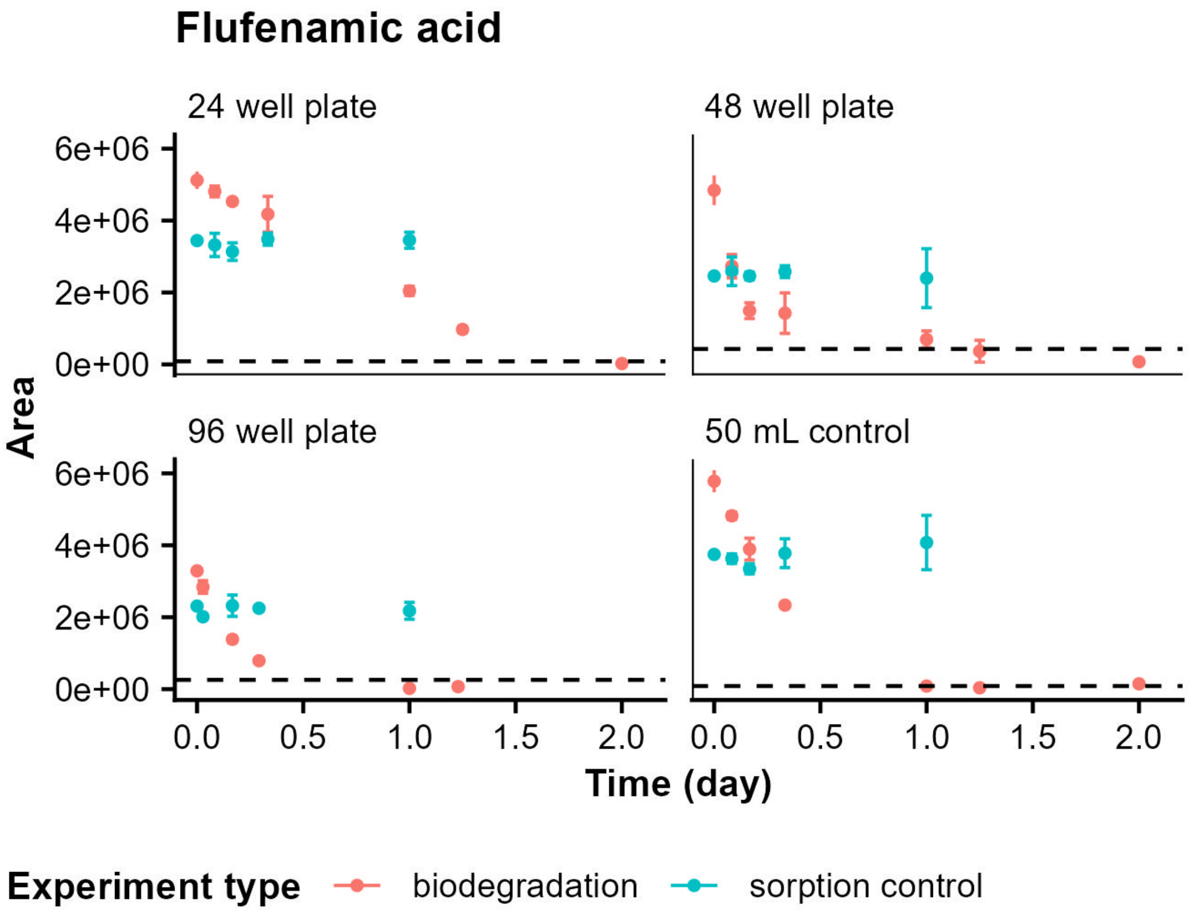
Flufenamic acid
24 well plate
48 well plate
96 well plate
50 mL control
6e+06
4e+06
2e+06
0e+00
Area
0.0
0.5
1.0
1.5
2.0
Time (day)
Experiment type
biodegradation
sorption control

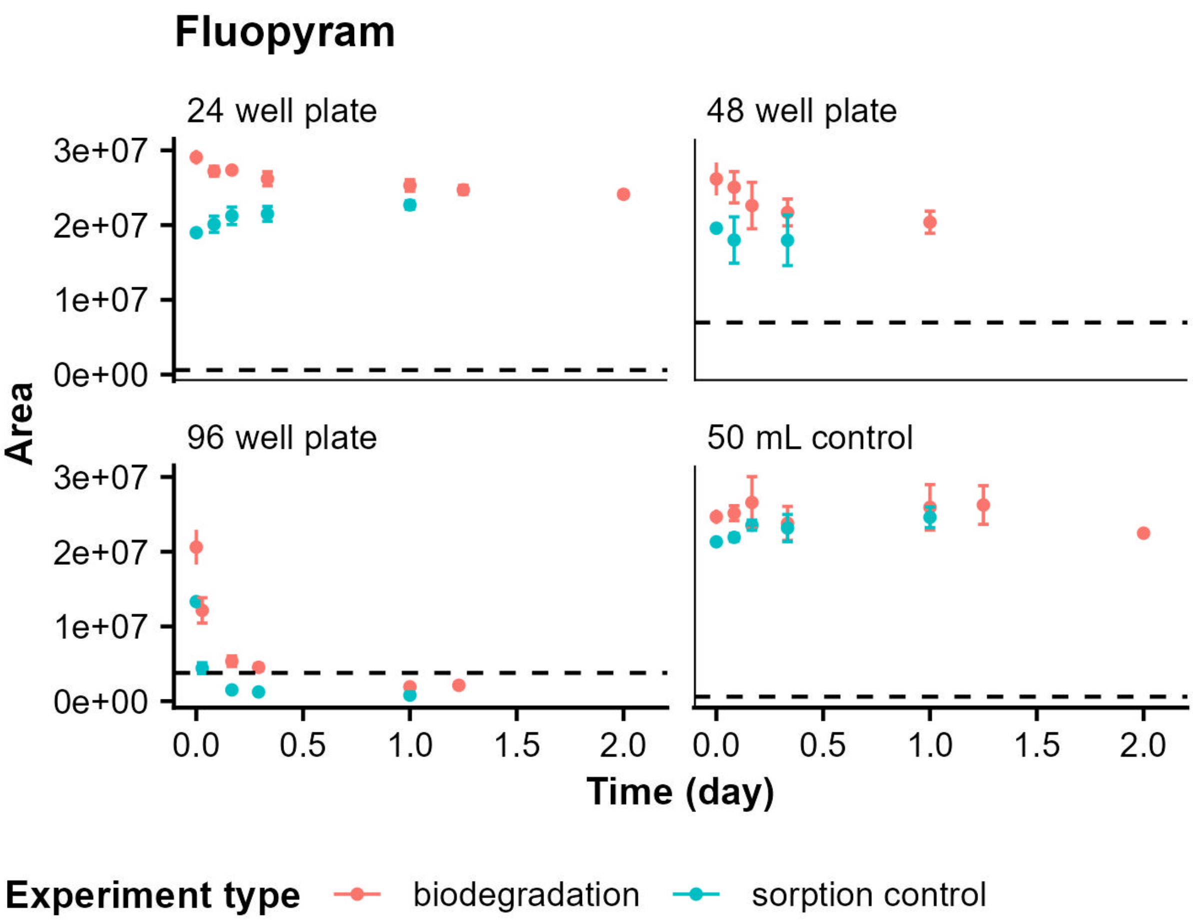
Fluopyram
24 well plate
48 well plate
96 well plate
50 mL control
3e+07
2e+07
1e+07
0e+00
Area
0.0
0.5
1.0
1.5
2.0
Time (day)
Experiment type
biodegradation
sorption control

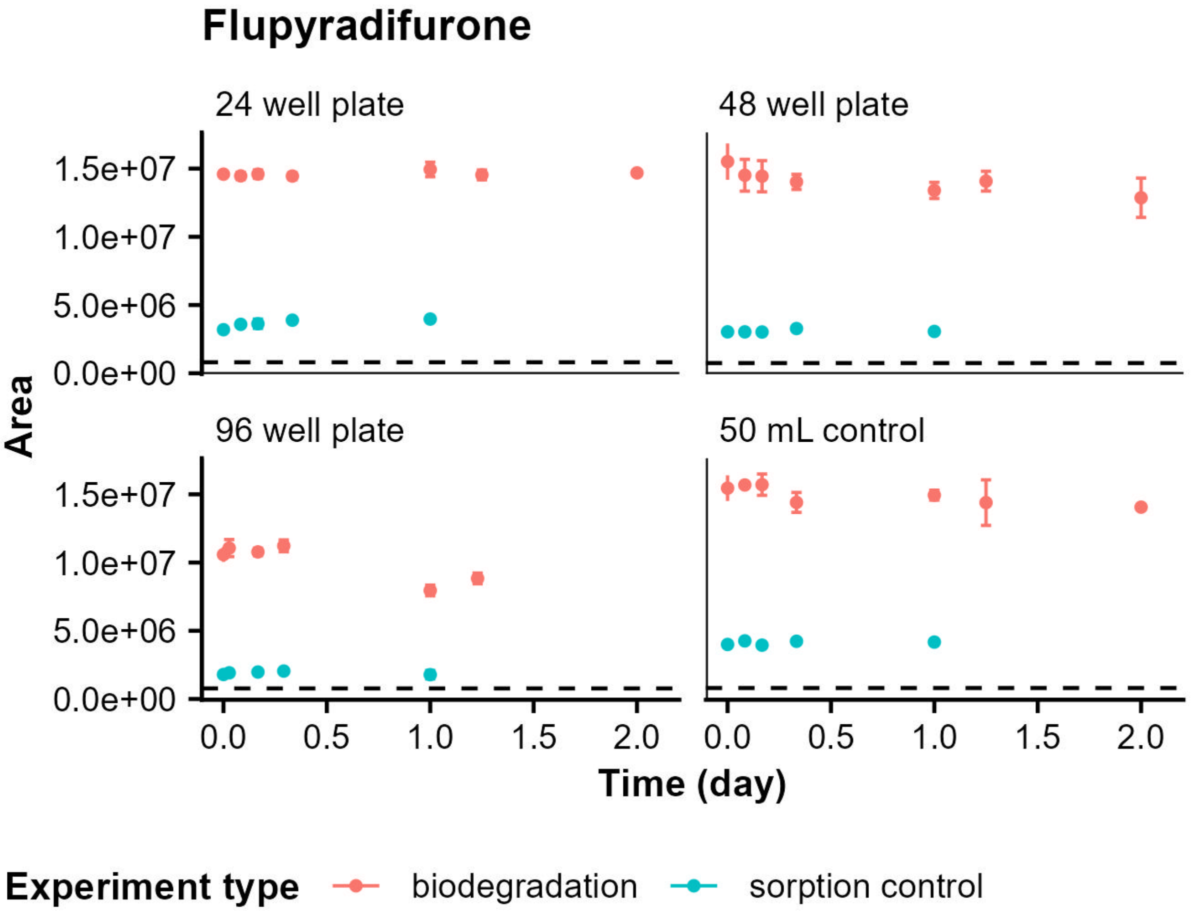
Flupyradifurone
24 well plate
48 well plate
96 well plate
50 mL control
1.5e+07
1.0e+07
5.0e+06
0.0e+00
Area
0.0
0.5
1.0
1.5
2.0
Time (day)
Experiment type
biodegradation
sorption control

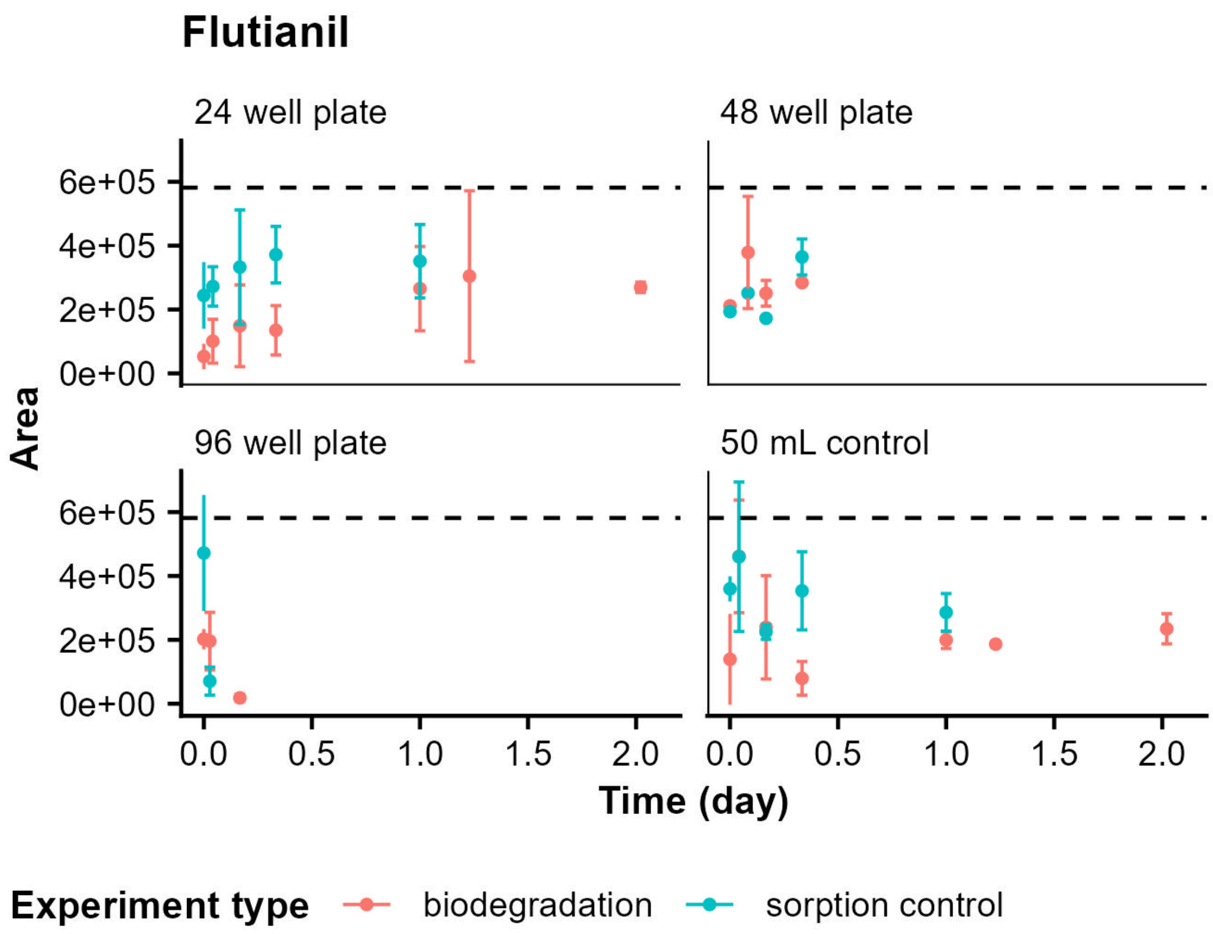
Flutianil
24 well plate
48 well plate
96 well plate
50 mL control
6e+05
4e+05
2e+05
0e+00
Area
0.0
0.5
1.0
1.5
2.0
Time (day)
Experiment type
biodegradation
sorption control

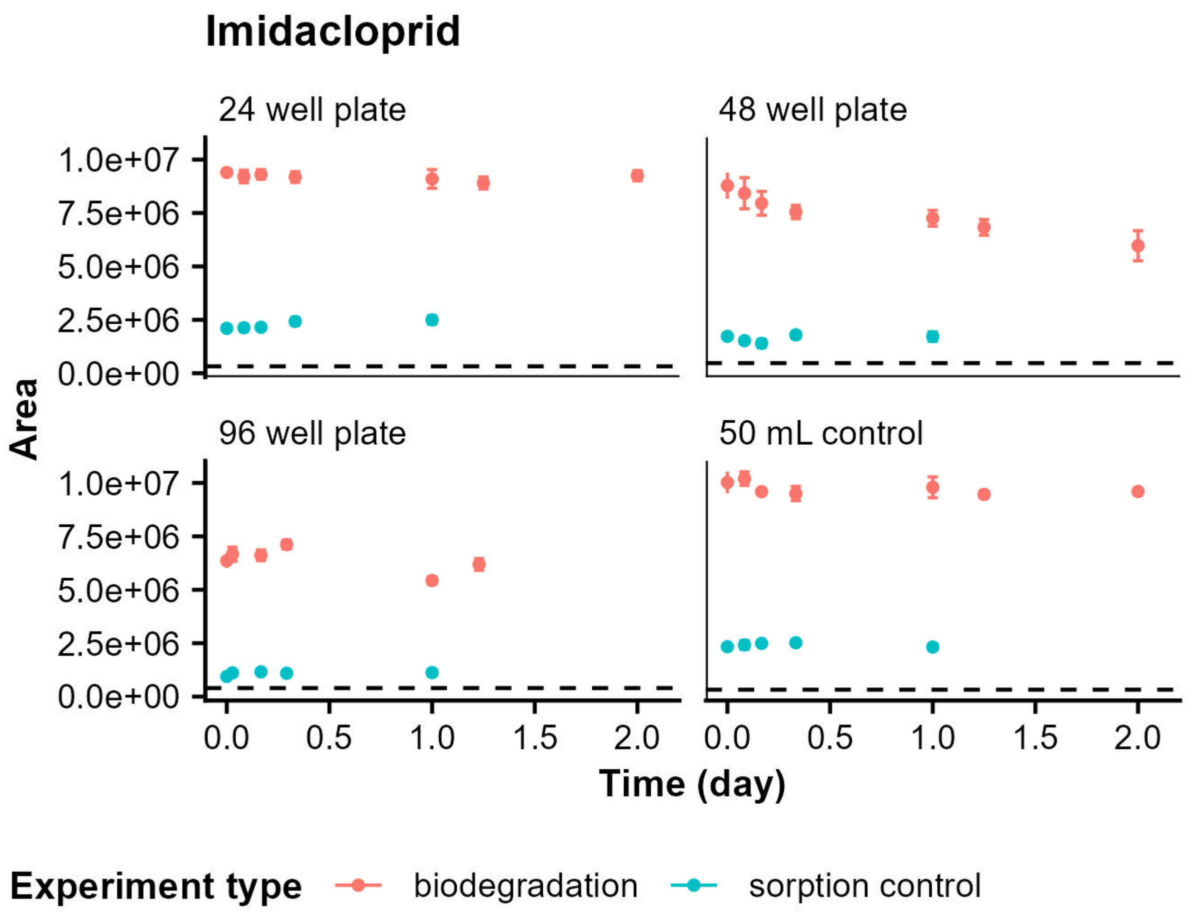
Imidacloprid
24 well plate
48 well plate
96 well plate
50 mL control
1.0e+07
7.5e+06
5.0e+06
2.5e+06
0.0e+00
Area
0.0
0.5
1.0
1.5
2.0
Time (day)
Experiment type
biodegradation
sorption control

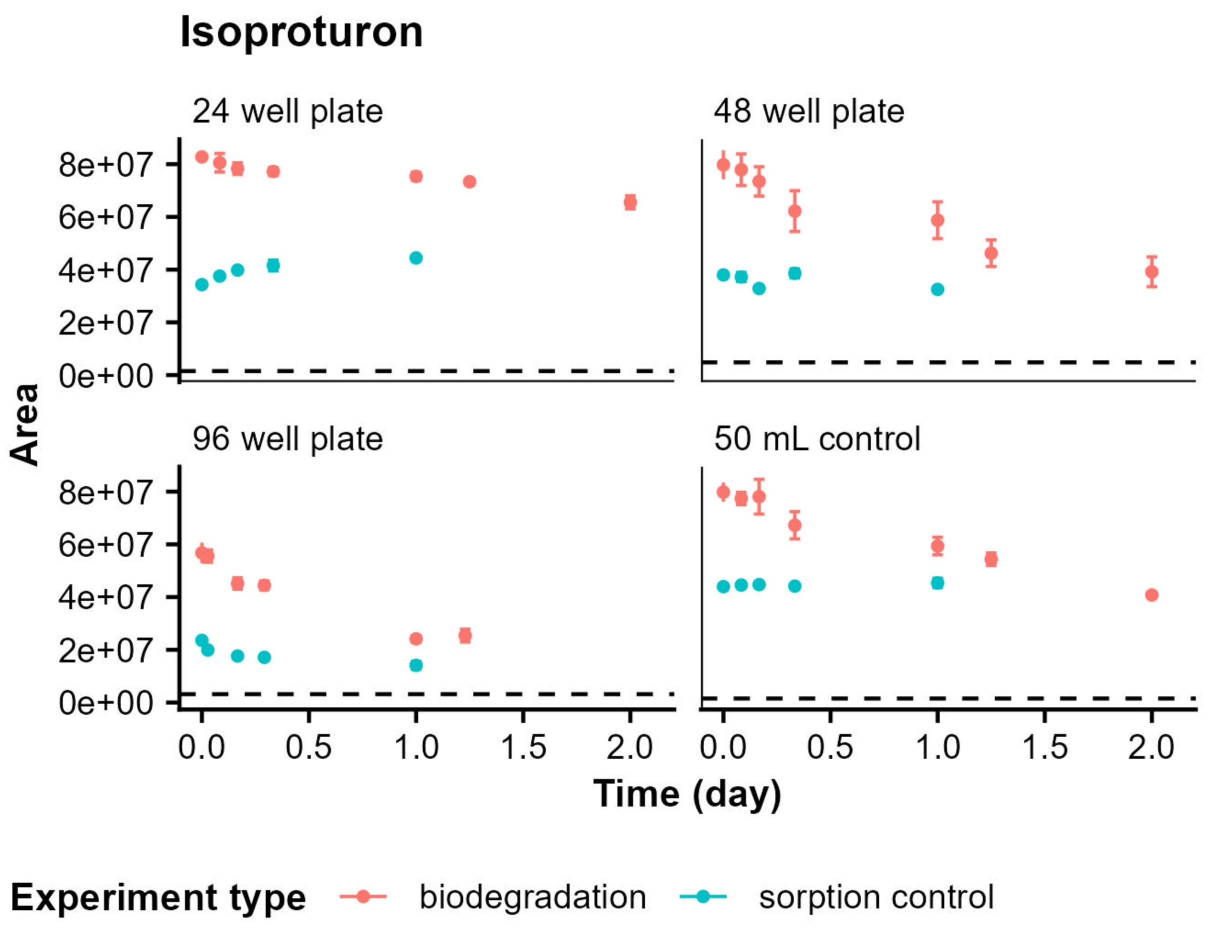
Isoproturon
24 well plate
48 well plate
96 well plate
50 mL control
Area
8e+07
6e+07
4e+07
2e+07
0e+00
0.0
0.5
1.0
1.5
2.0
Time (day)
Experiment type
biodegradation
sorption control

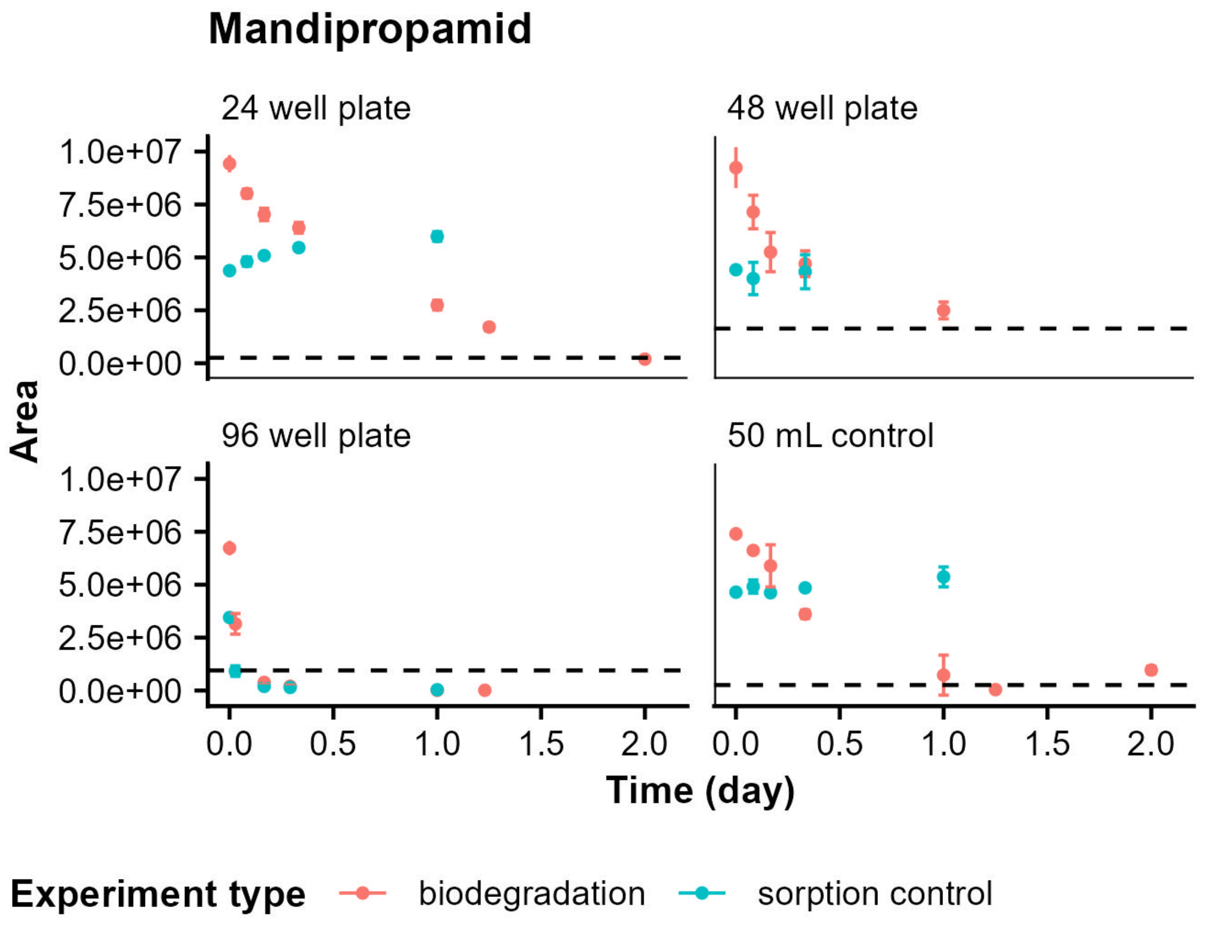
Mandipropamid
24 well plate
48 well plate
96 well plate
50 mL control
Area
1.0e+07
7.5e+06
5.0e+06
2.5e+06
0.0e+00
0.0
0.5
1.0
1.5
2.0
Time (day)
Experiment type
biodegradation
sorption control

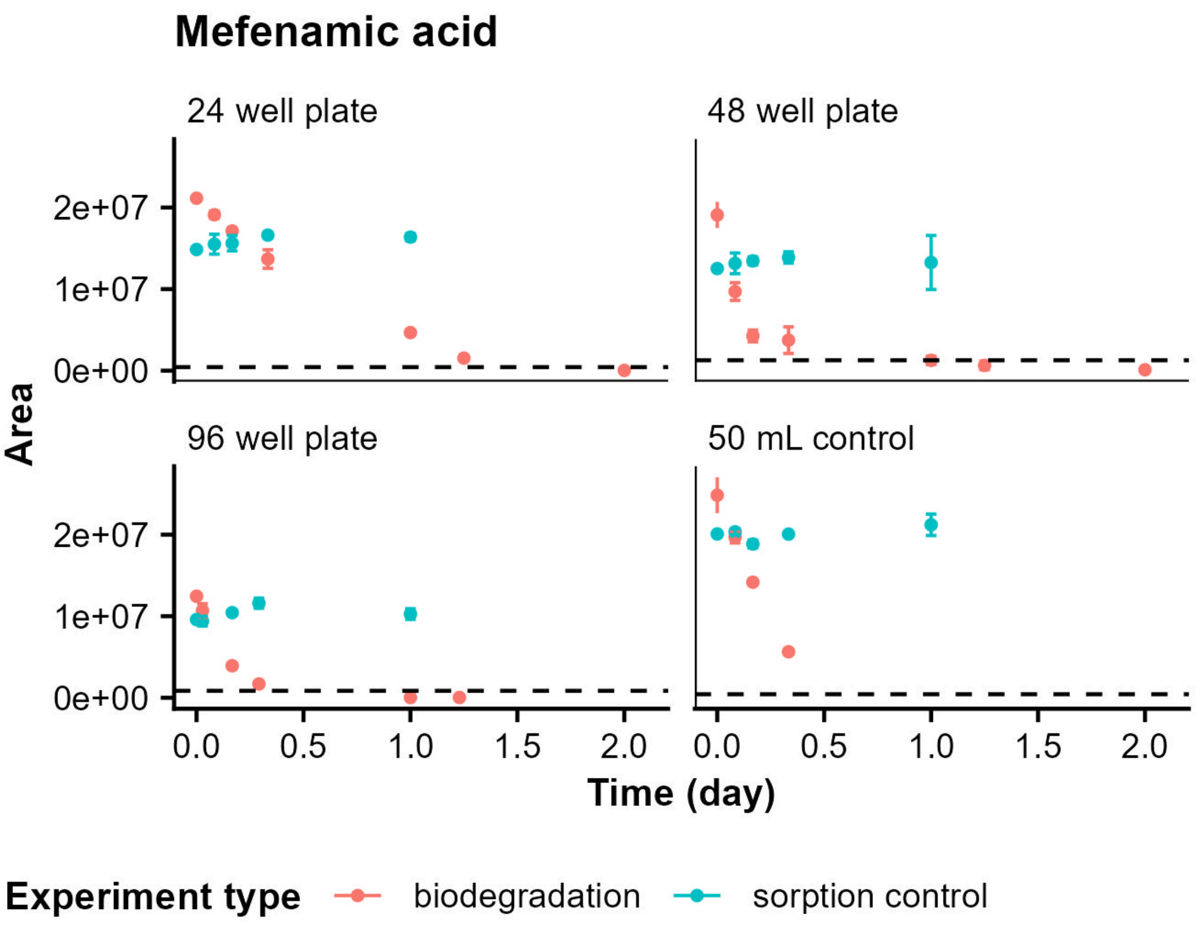
Mefenamic acid
24 well plate
48 well plate
96 well plate
50 mL control
2e+07
1e+07
0e+00
Area
0.0
0.5
1.0
1.5
2.0
Time (day)
Experiment type
biodegradation
sorption control

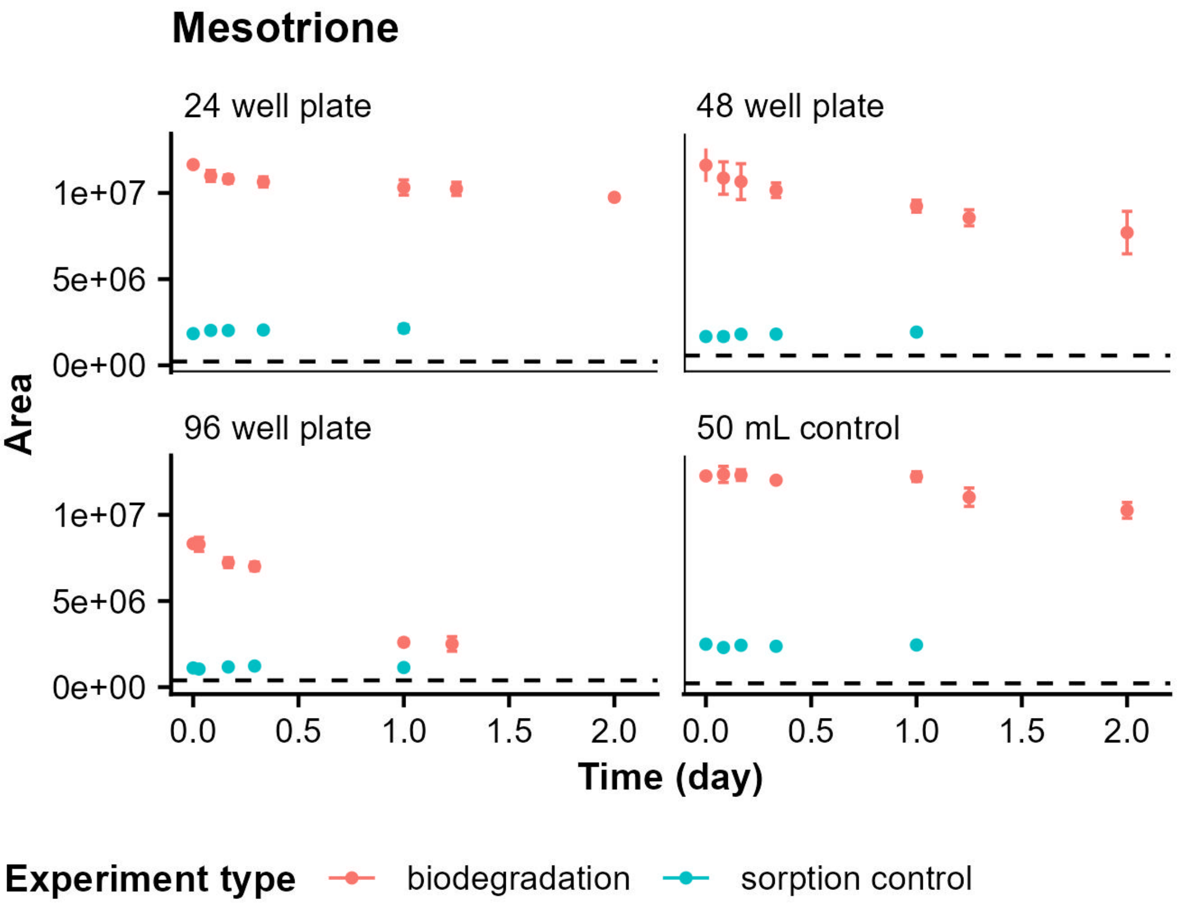
Mesotrione
24 well plate
48 well plate
96 well plate
50 mL control
1e+07
5e+06
0e+00
Area
0.0
0.5
1.0
1.5
2.0
Time (day)
Experiment type
biodegradation
sorption control

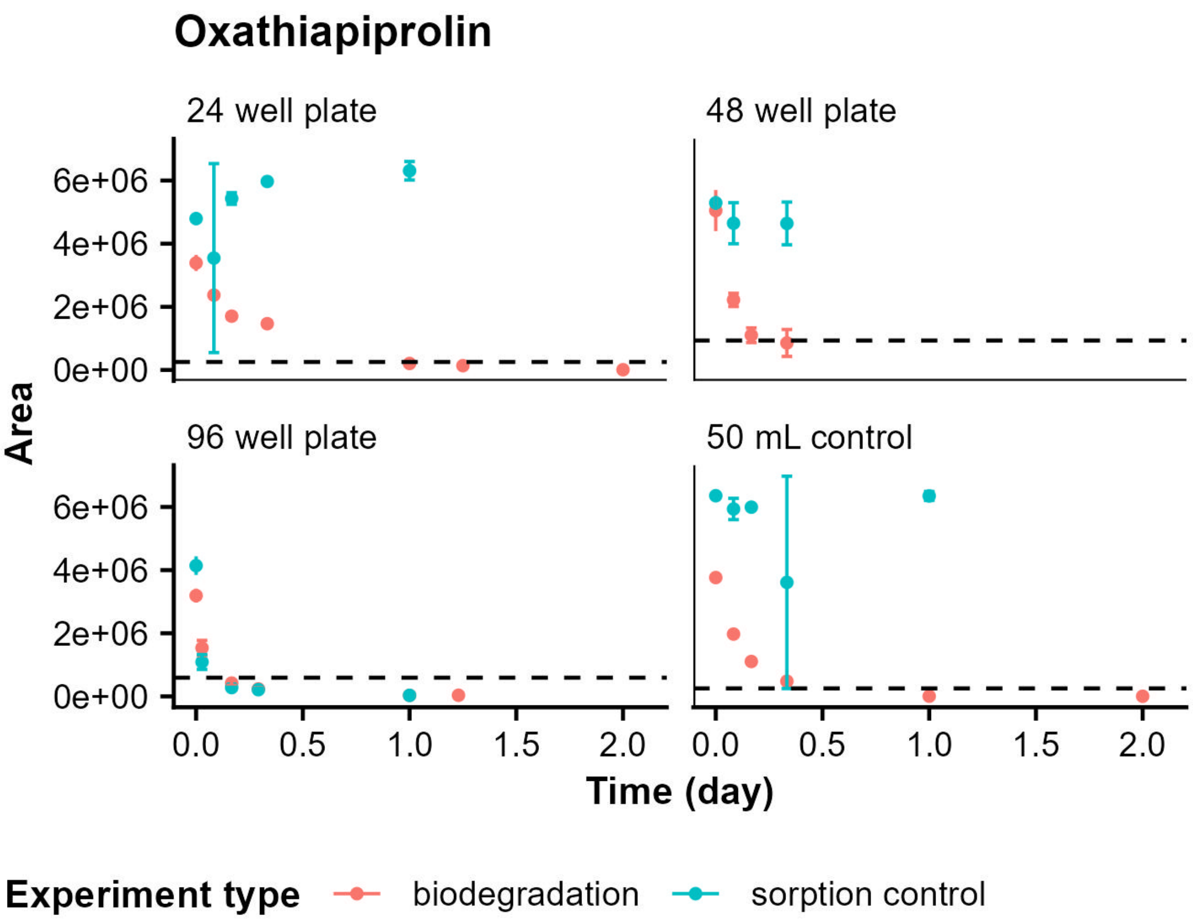
Oxathiapiprolin
24 well plate
48 well plate
96 well plate
50 mL control
6e+06
4e+06
2e+06
0e+00
Area
0.0
0.5
1.0
1.5
2.0
Time (day)
Experiment type
biodegradation
sorption control

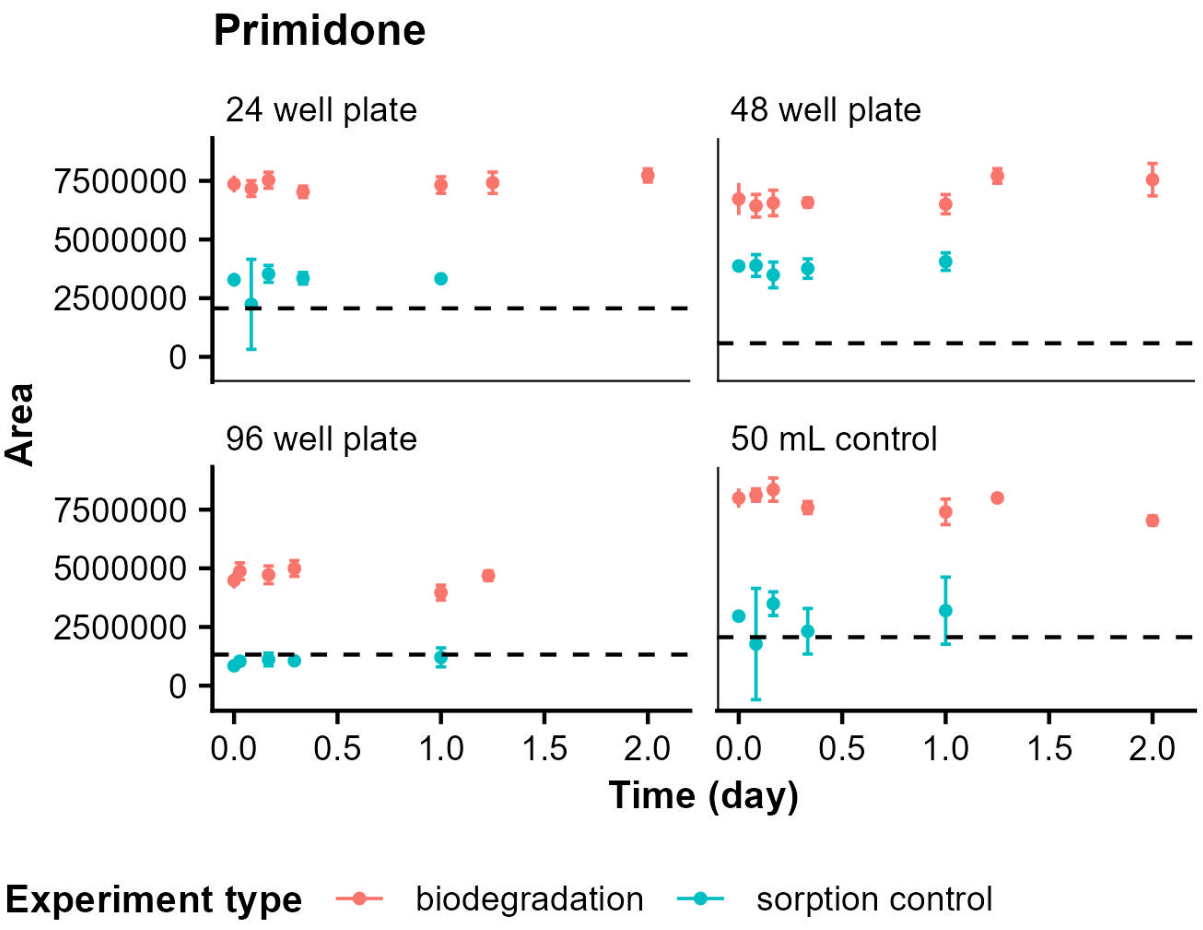
Primidone
24 well plate
48 well plate
96 well plate
50 mL control
7500000
5000000
2500000
0
Area
0.0
0.5
1.0
1.5
2.0
Time (day)
Experiment type
biodegradation
sorption control

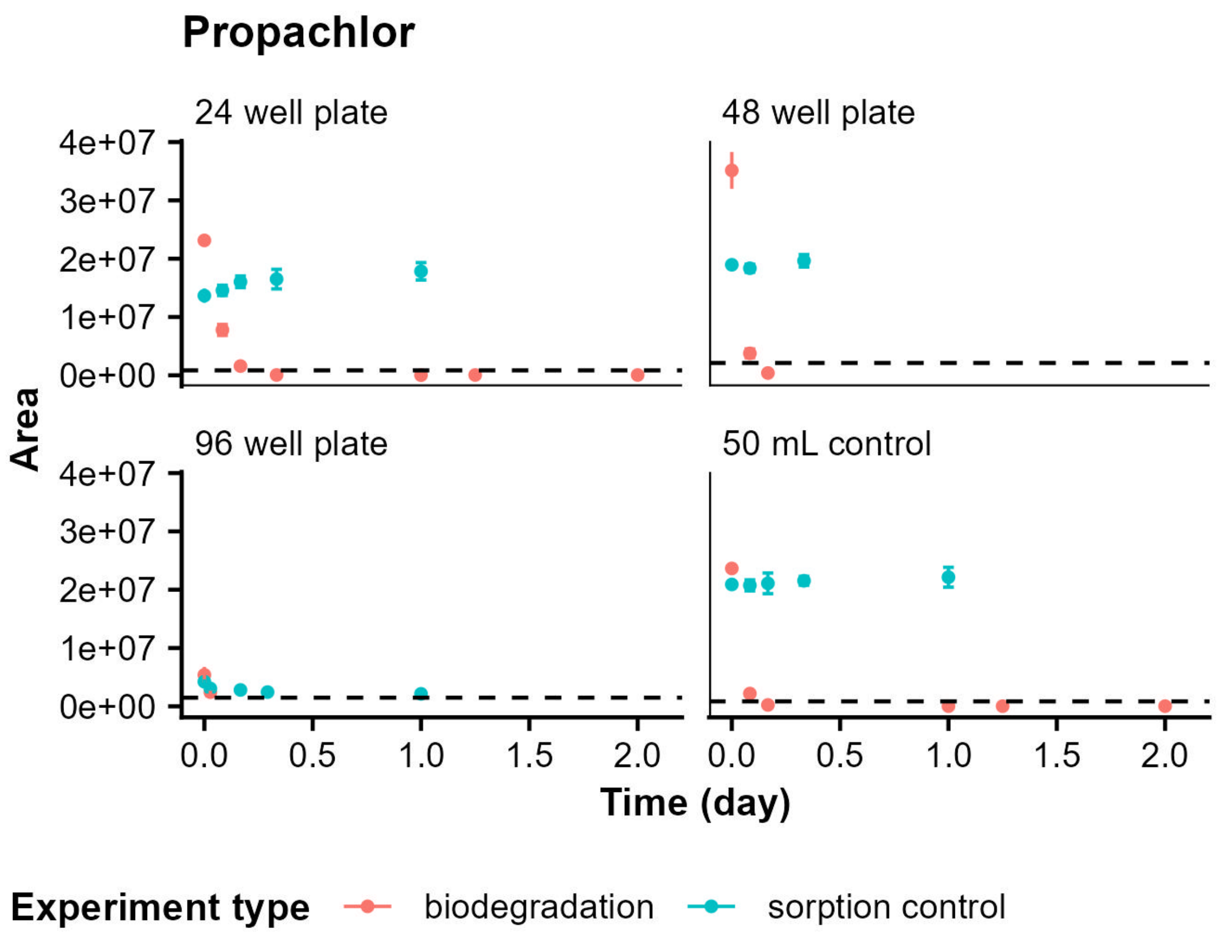
Propachlor
24 well plate
48 well plate
96 well plate
50 mL control
4e+07
3e+07
2e+07
1e+07
0e+00
Area
0.0
0.5
1.0
1.5
2.0
Time (day)
Experiment type
biodegradation
sorption control

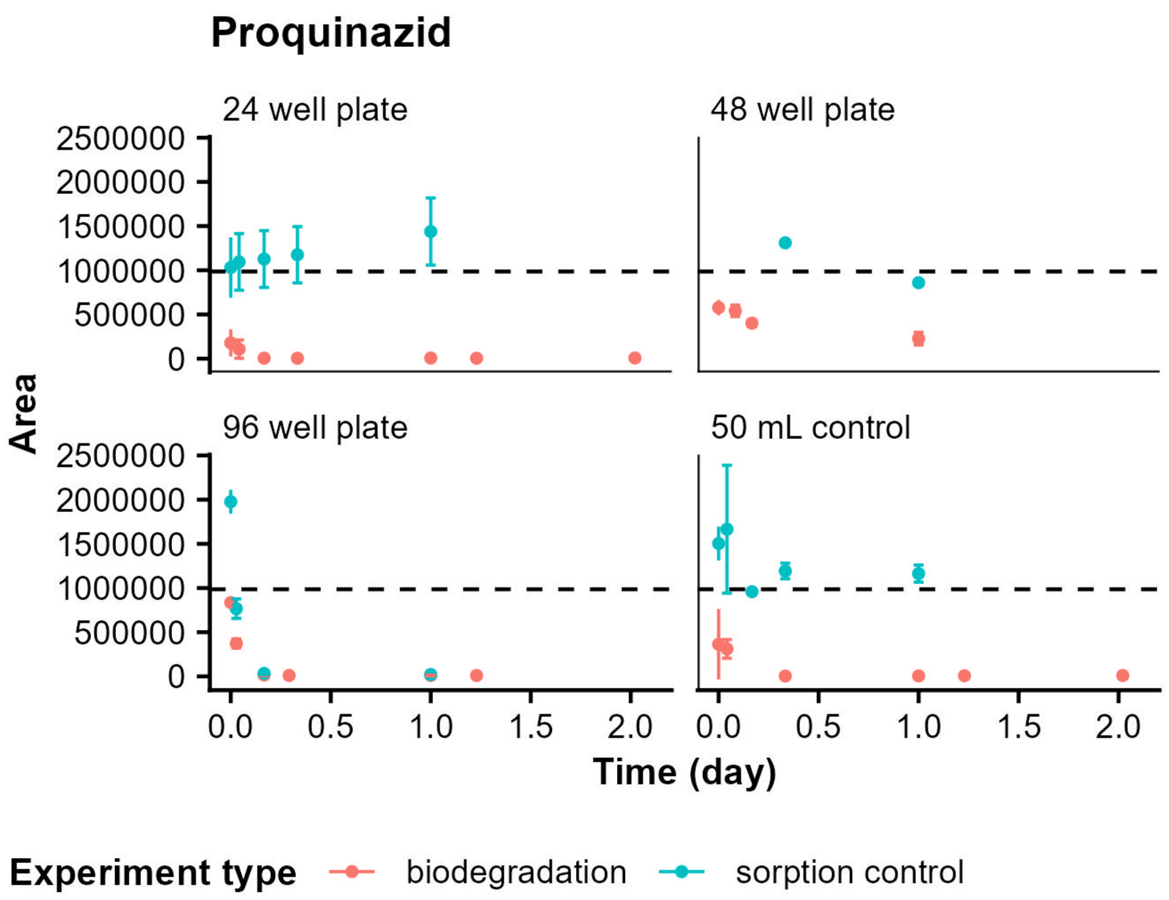
Proquinazid
24 well plate
48 well plate
96 well plate
50 mL control
2500000
2000000
1500000
1000000
500000
0
Area
0.0
0.5
1.0
1.5
2.0
Time (day)
Experiment type
biodegradation
sorption control

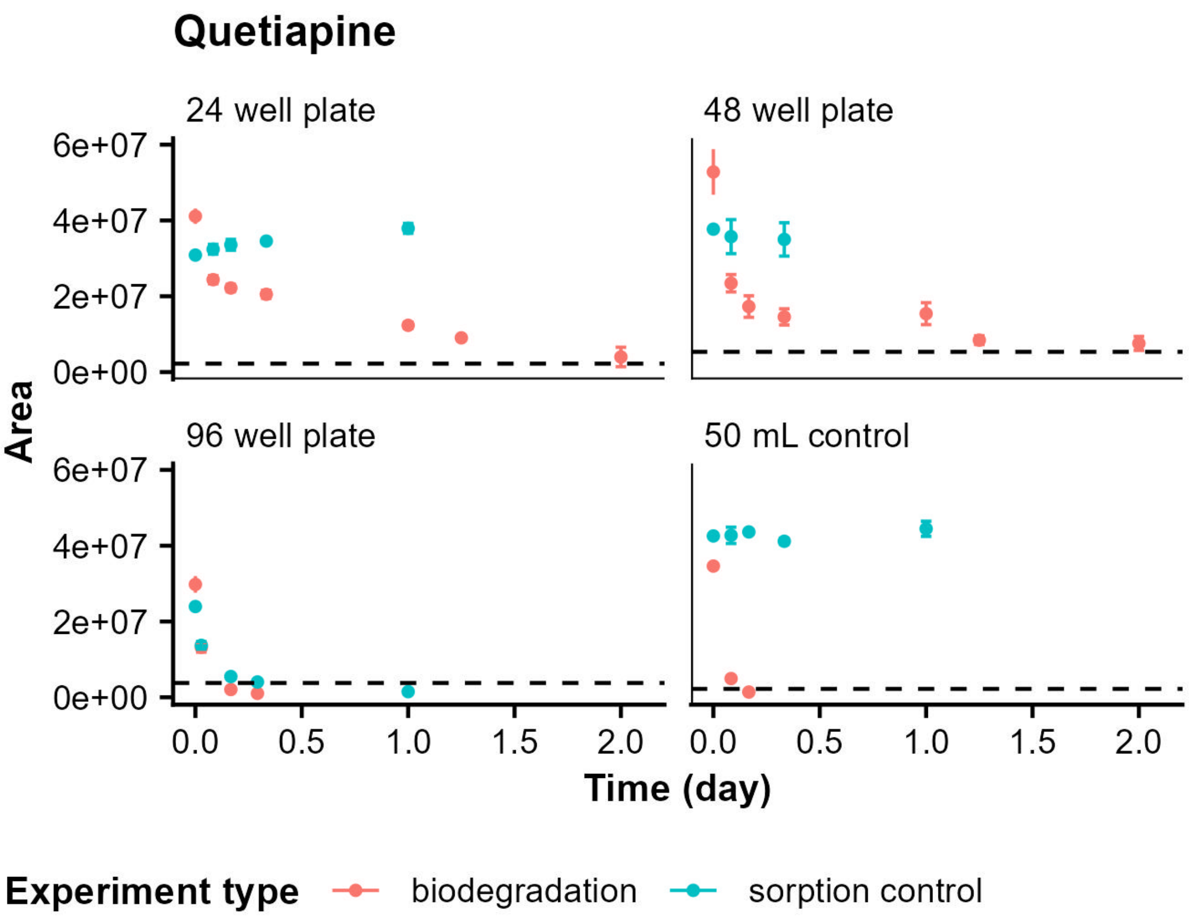
Quetiapine
24 well plate
48 well plate
96 well plate
50 mL control
6e+07
4e+07
2e+07
0e+00
Area
0.0
0.5
1.0
1.5
2.0
Time (day)
Experiment type
biodegradation
sorption control

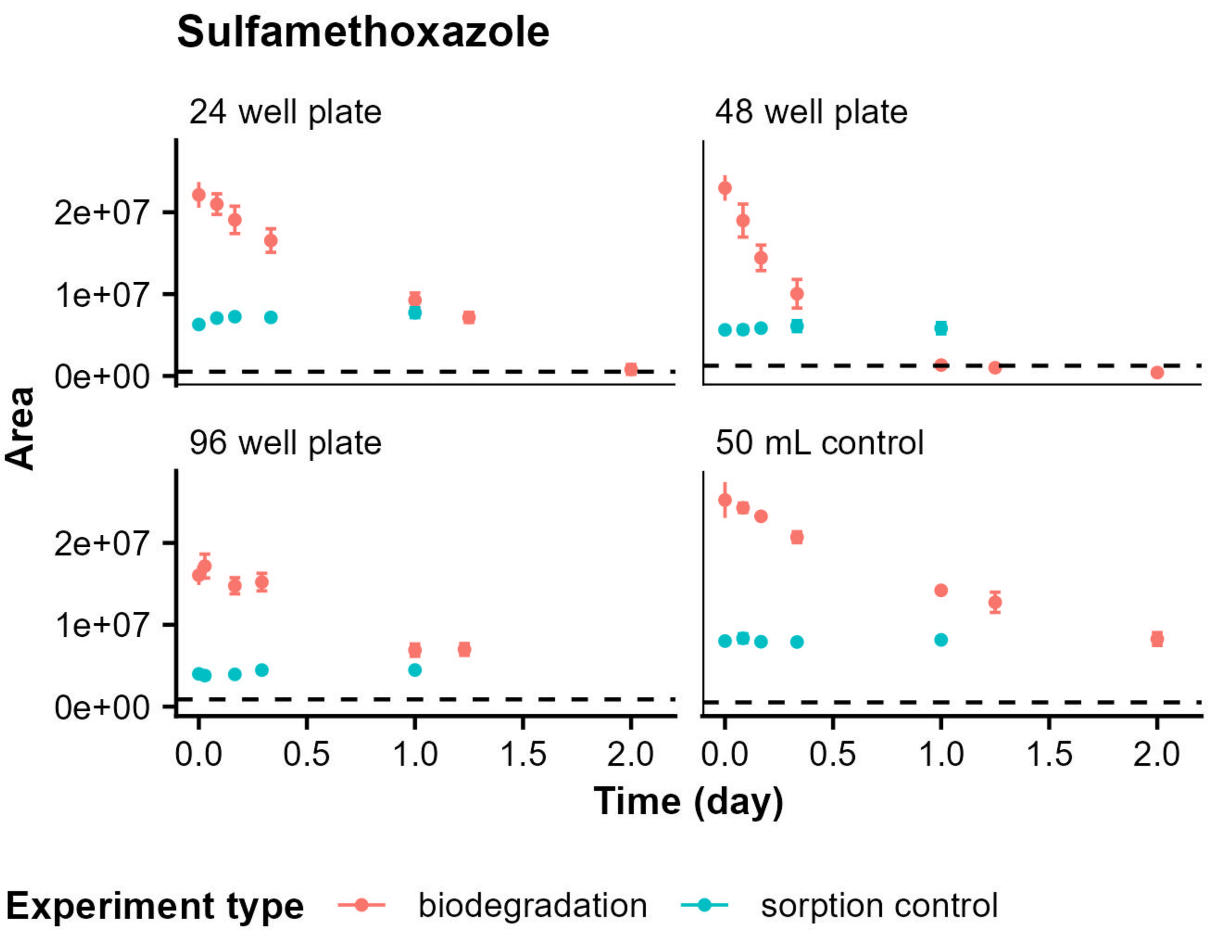
Sulfamethoxazole
24 well plate
48 well plate
96 well plate
50 mL control
2e+07
1e+07
0e+00
Area
0.0
0.5
1.0
1.5
2.0
Time (day)
Experiment type
biodegradation
sorption control

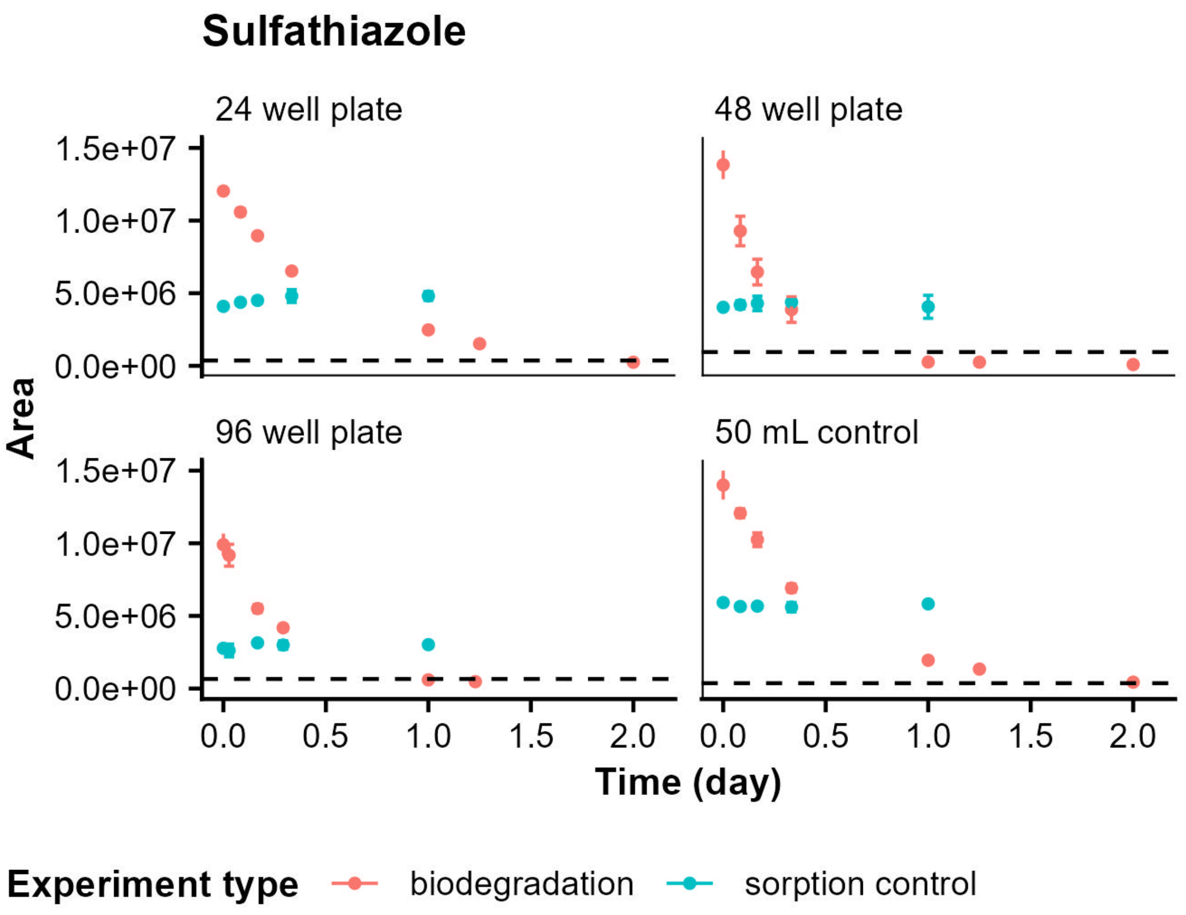
Sulfathiazole
24 well plate
48 well plate
96 well plate
50 mL control
1.5e+07
1.0e+07
5.0e+06
0.0e+00
Area
0.0
0.5
1.0
1.5
2.0
Time (day)
Experiment type
biodegradation
sorption control

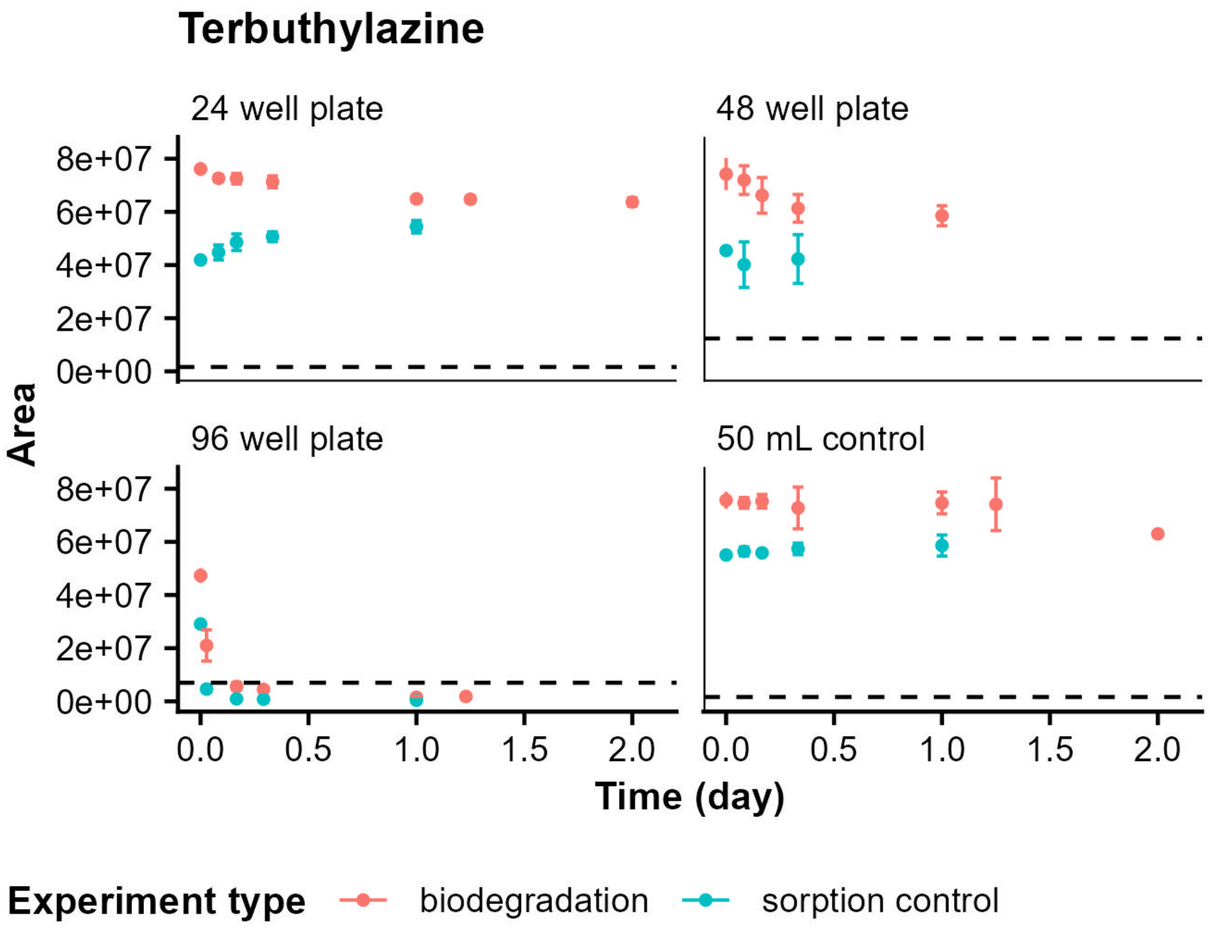
Terbuthylazine
24 well plate
48 well plate
96 well plate
50 mL control
8e+07
6e+07
4e+07
2e+07
0e+00
Area
0.0
0.5
1.0
1.5
2.0
Time (day)
Experiment type
biodegradation
sorption control

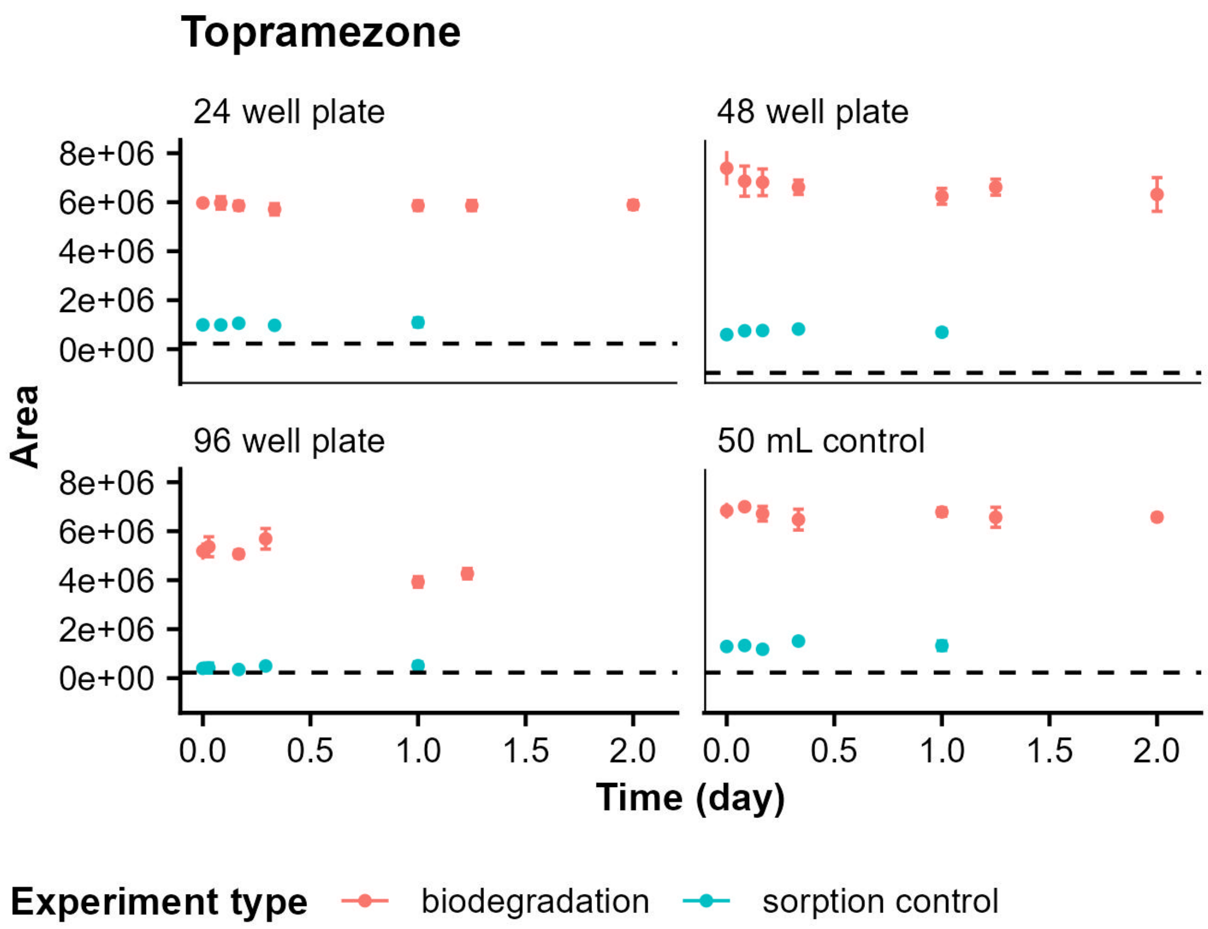
Topramezone
24 well plate
48 well plate
96 well plate
50 mL control
8e+06
6e+06
4e+06
2e+06
0e+00
Area
0.0
0.5
1.0
1.5
2.0
Time (day)
Experiment type
biodegradation
sorption control

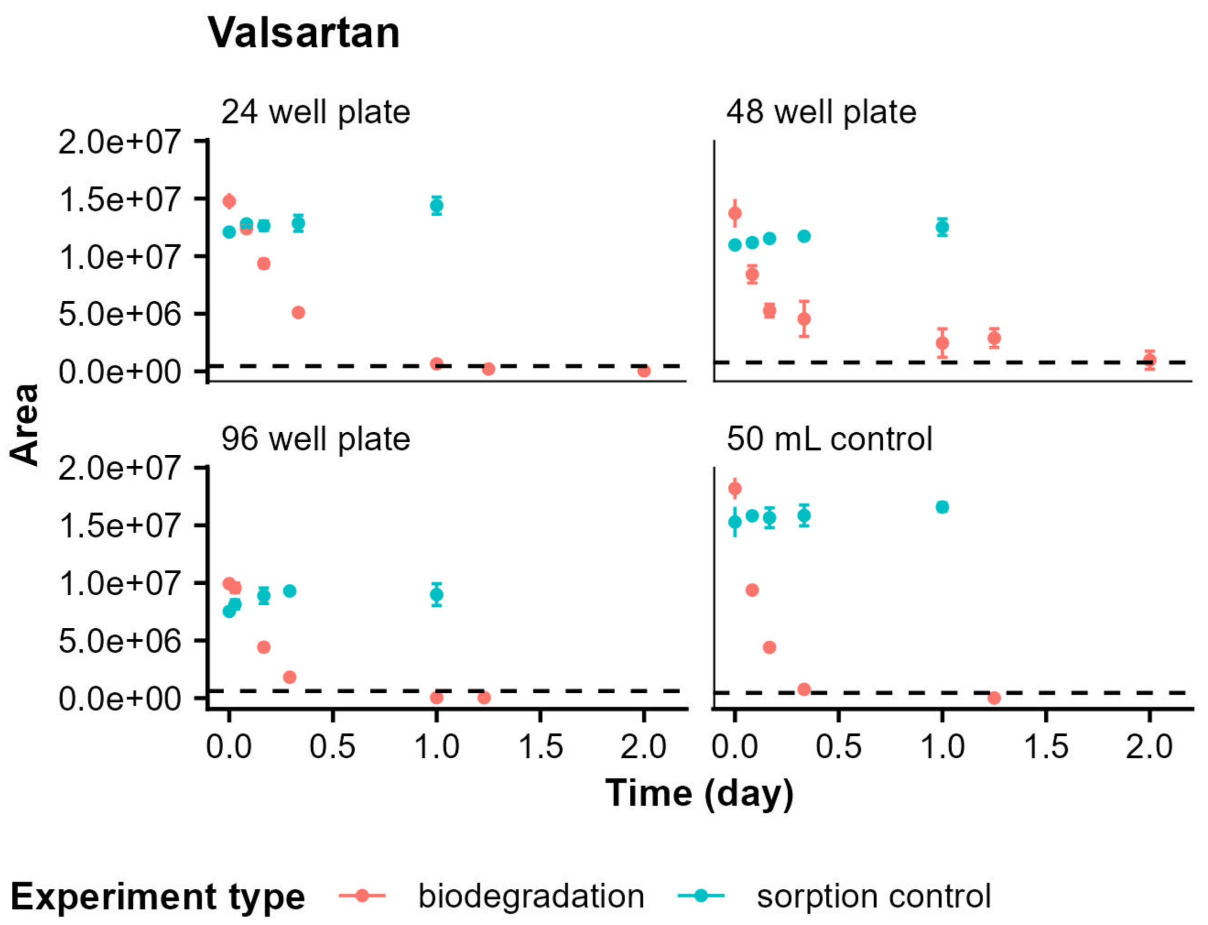
Valsartan
24 well plate
48 well plate
96 well plate
50 mL control
2.0e+07
1.5e+07
1.0e+07
5.0e+06
0.0e+00
Area
0.0
0.5
1.0
1.5
2.0
Time (day)
Experiment type
biodegradation
sorption control

**Figure S2.1.** Kinetic area-time series plots for each test compound at four experimental scales (24 well plate, 48 well plate, 96 well plate, and 50 mL (i.e. large volume) control). Orange points show biotransformation data, blue points show data from autoclaved activated sludge (sorption controls). The dotted line represents the calculated LOQ for each experiment. Representative experiments were chosen for each scale (96 well plate data from experiment 4, 48 well plate data from experiment 5, and 24 well plate and 50 mL control data from experiment 6; See Table S1.3 for experimental details), with the exception of fenoxycarb, flutianil, and proquinazid, where data for 24 well plate and 50 mL control were taken from experiment 4.

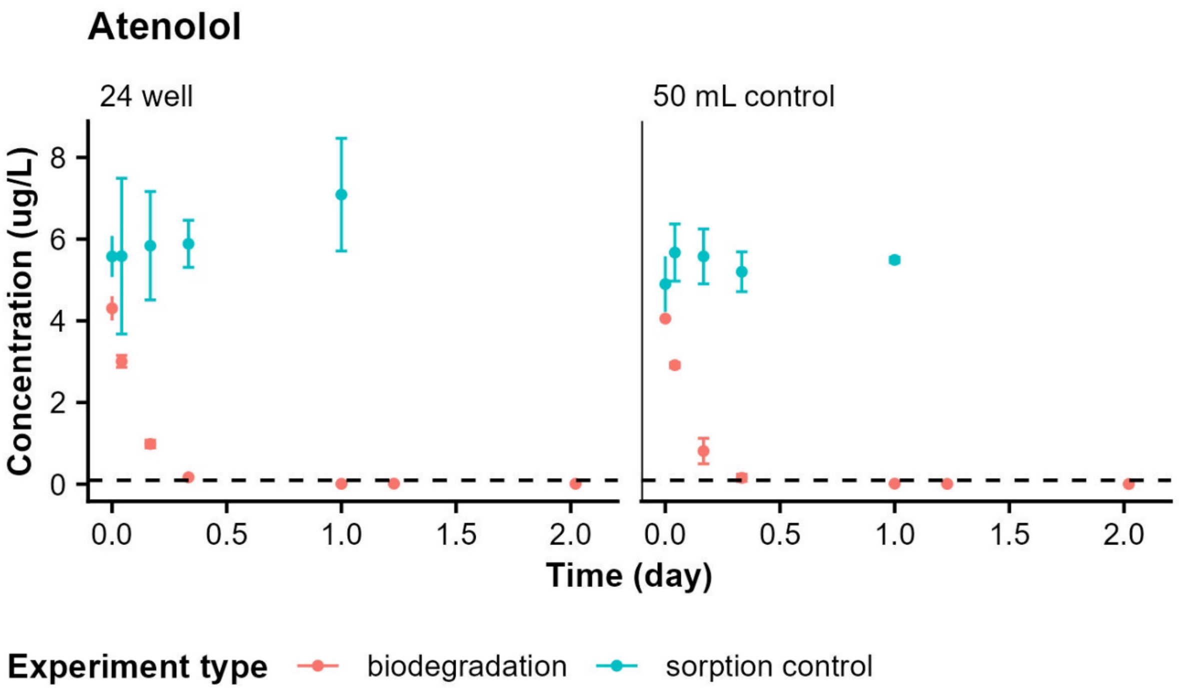
Atenolol
24 well
50 mL control
Concentration (ug/L)
0
2
4
6
8
0.0
0.5
1.0
1.5
2.0
Time (day)
Experiment type
biodegradation
sorption control

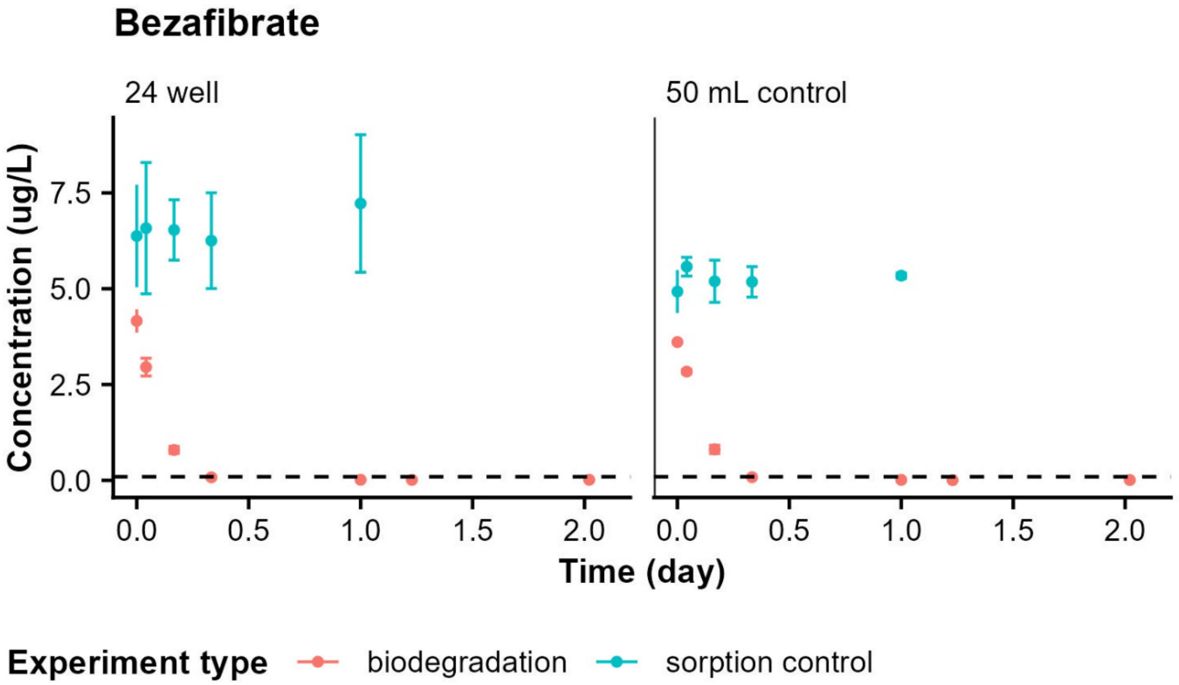
Bezafibrate
24 well
50 mL control
Concentration (ug/L)
0.0
2.5
5.0
7.5
0.0
0.5
1.0
1.5
2.0
Time (day)
Experiment type
biodegradation
sorption control

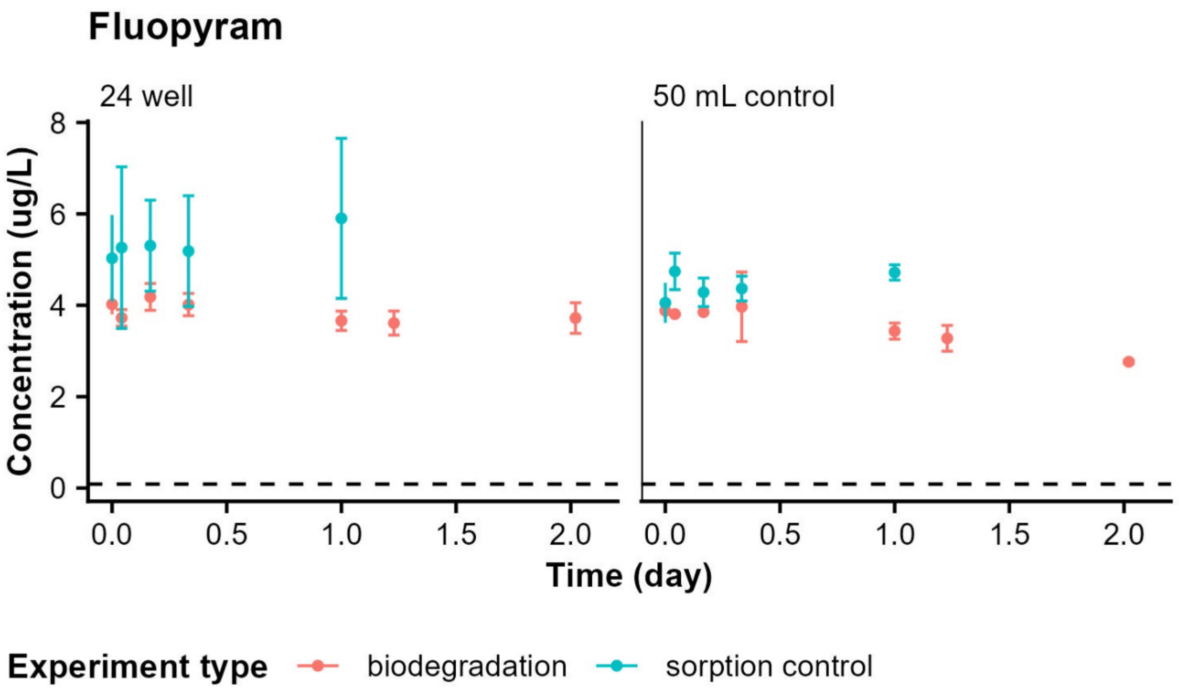
Fluopyram
24 well
50 mL control
Concentration (ug/L)
0
2
4
6
8
0.0
0.5
1.0
1.5
2.0
Time (day)
Experiment type
biodegradation
sorption control

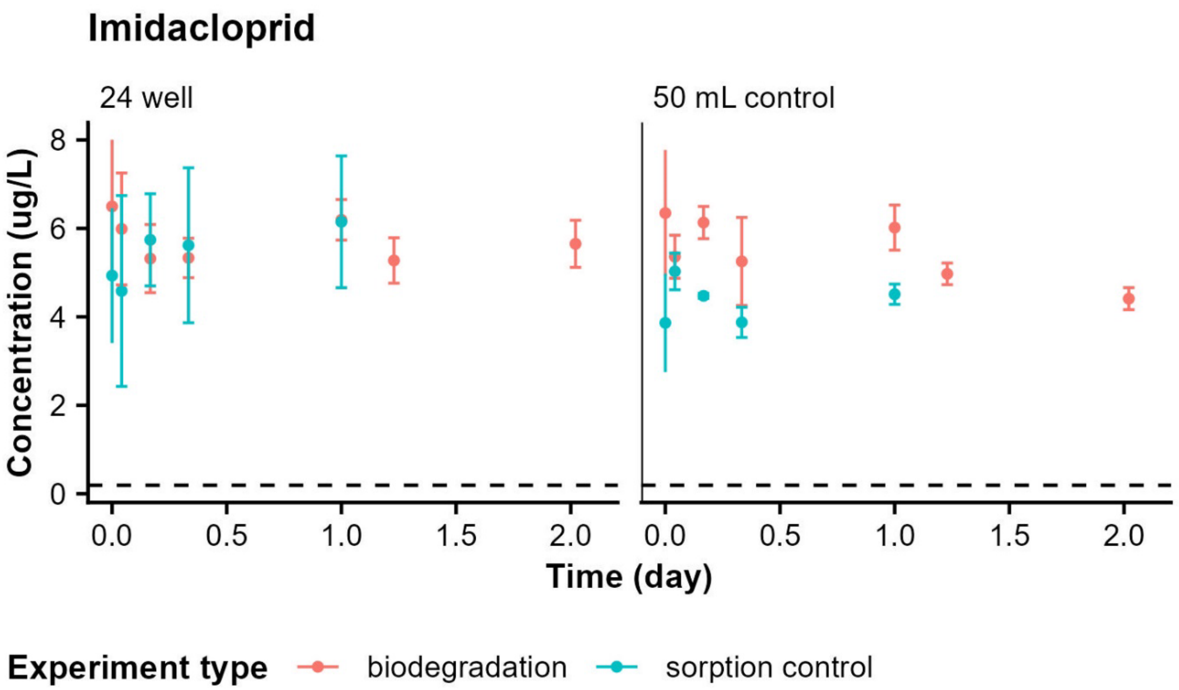
Imidacloprid
24 well
50 mL control
Concentration (ug/L)
0
2
4
6
8
0.0
0.5
1.0
1.5
2.0
Time (day)
Experiment type
biodegradation
sorption control

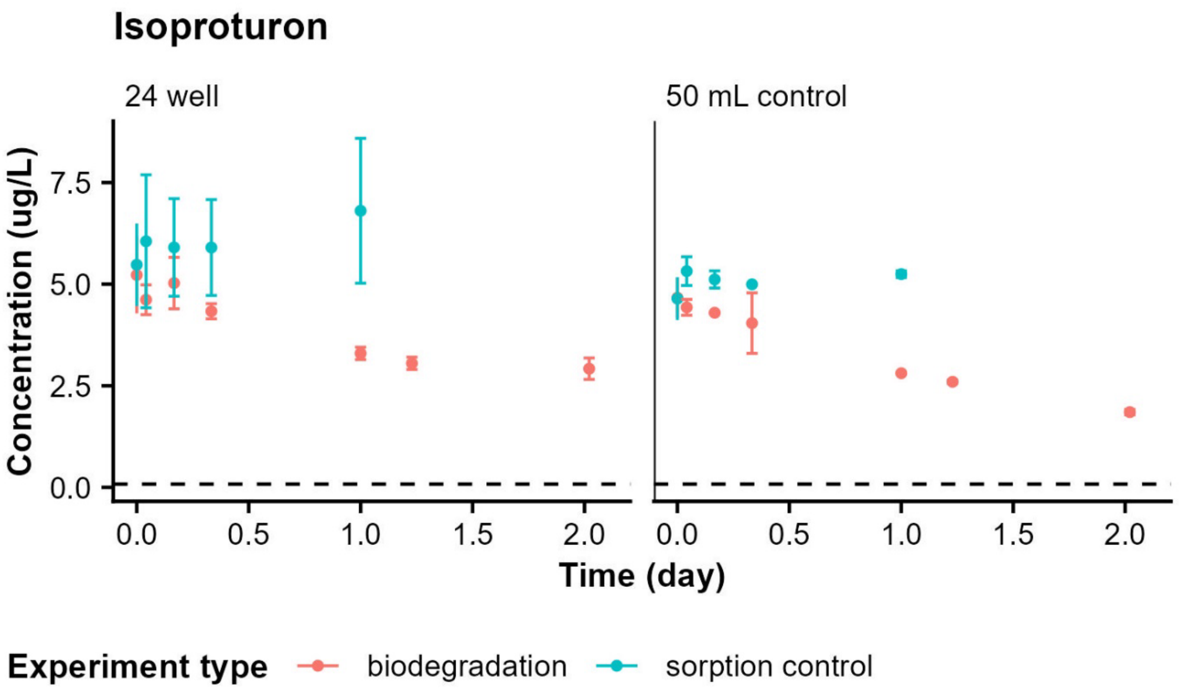
Isoproturon
24 well
50 mL control
Concentration (ug/L)
7.5
5.0
2.5
0.0
0.0
0.5
1.0
1.5
2.0
Time (day)
Experiment type
biodegradation
sorption control

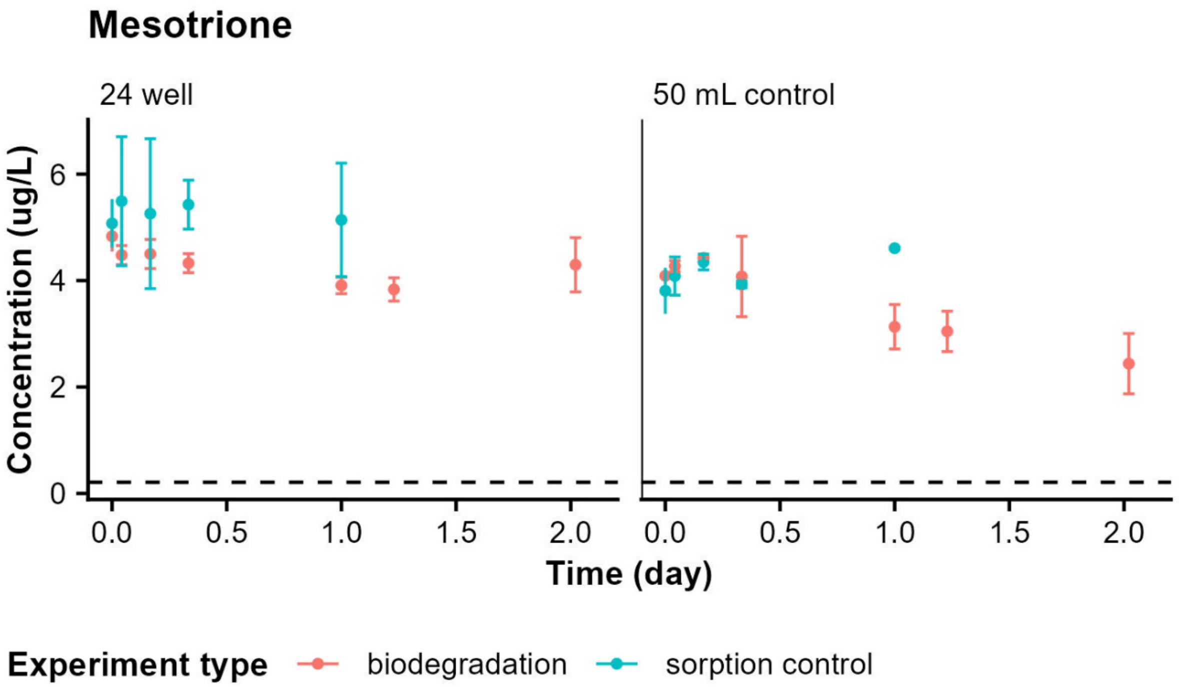
Mesotrione
24 well
50 mL control
Concentration (ug/L)
6
4
2
0
0.0
0.5
1.0
1.5
2.0
Time (day)
Experiment type
biodegradation
sorption control

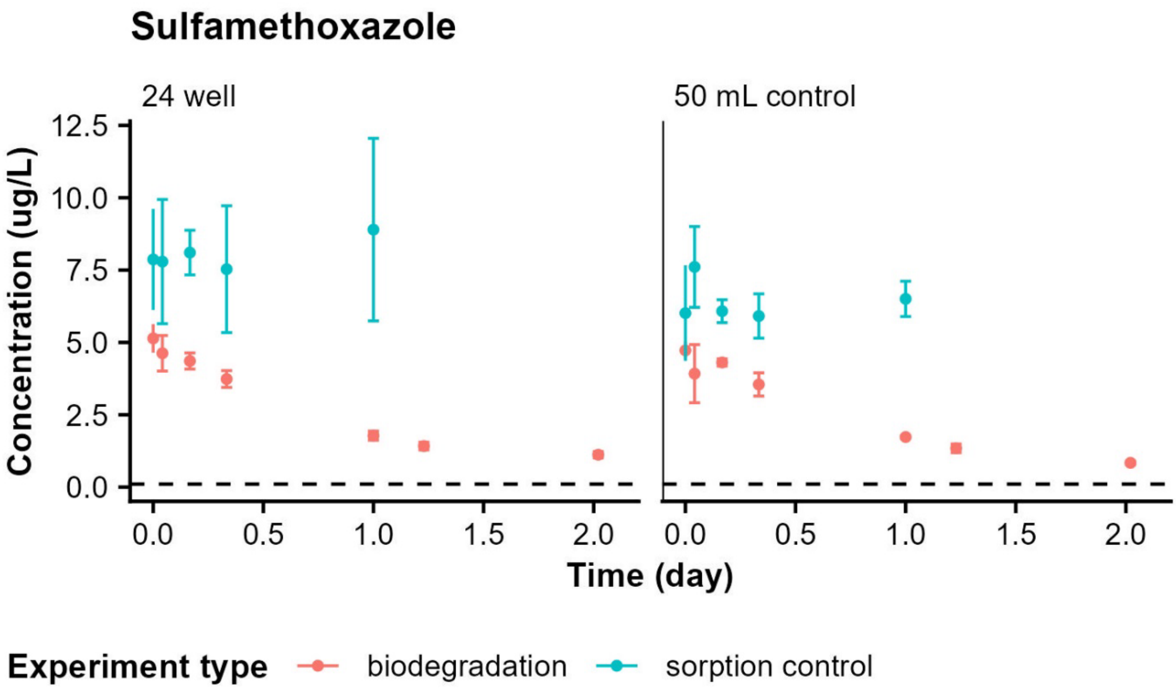

Sulfamethoxazole
24 well
50 mL control
Concentration (ug/L)
12.5
10.0
7.5
5.0
2.5
0.0
0.0
0.5
1.0
1.5
2.0
Time (day)
Experiment type
biodegradation
sorption control


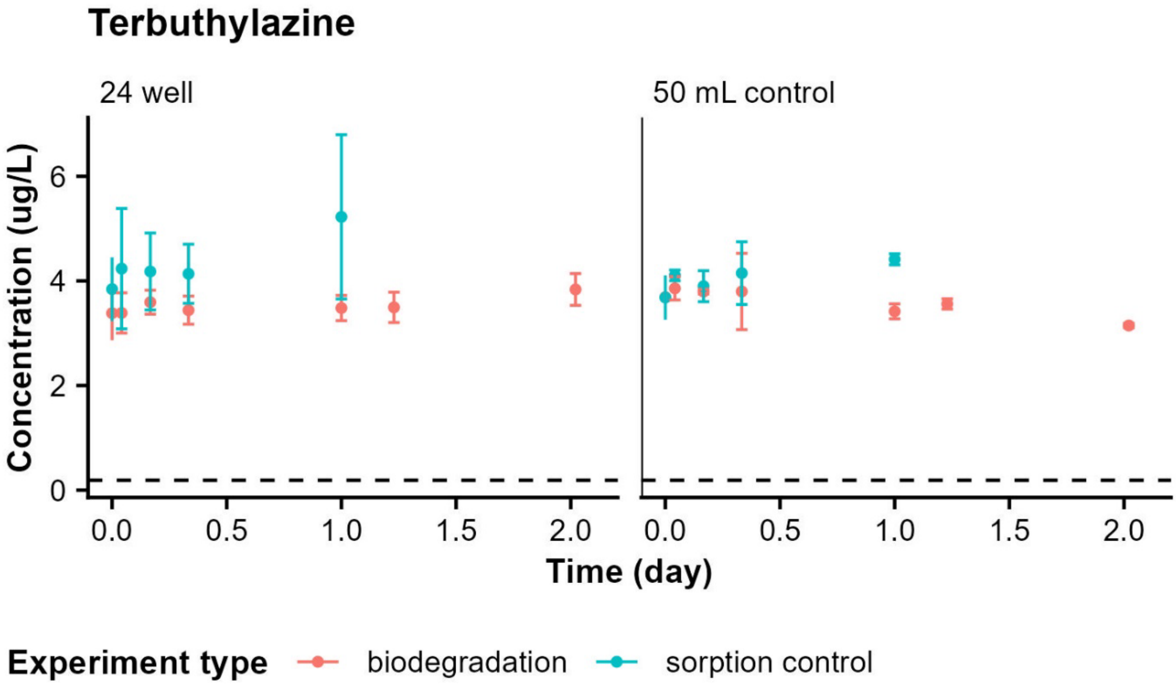

Terbuthylazine
24 well
50 mL control
Concentration (ug/L)
6
4
2
0
0.0
0.5
1.0
1.5
2.0
Time (day)
Experiment type
biodegradation
sorption control

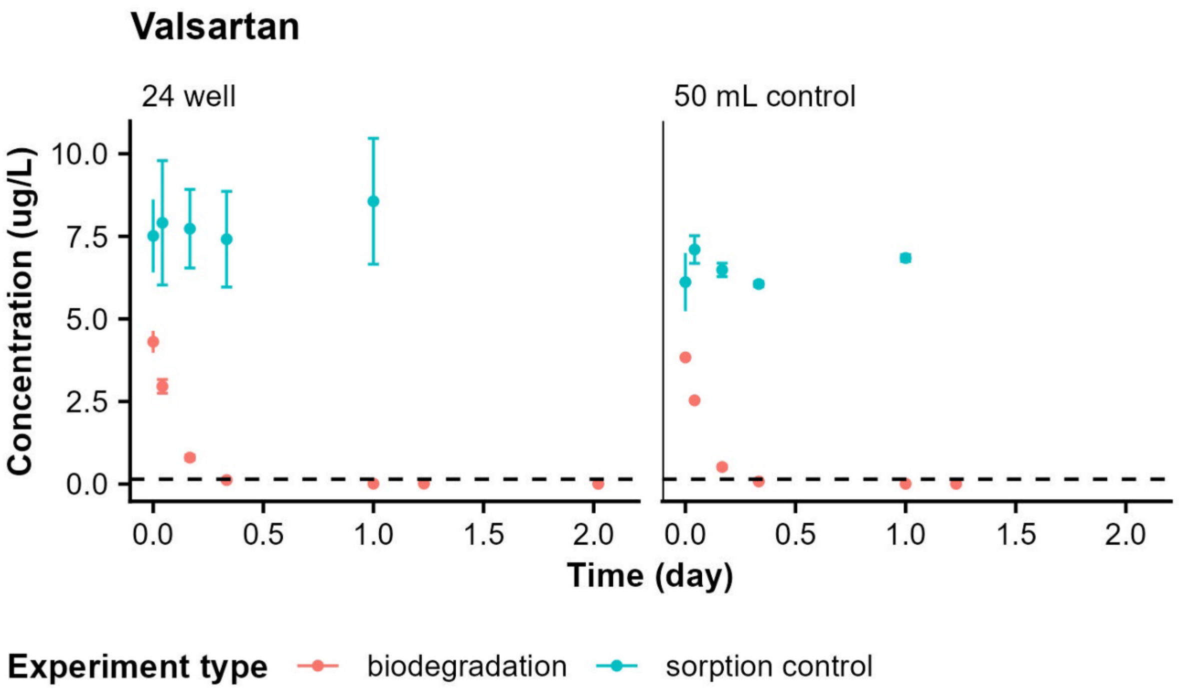


**Figure S2.2.** Kinetic concentration-time series plots for each test compound at two experimental scales (24 well plate and 50 mL (i.e. large volume) control). Orange points show biotransformation data, blue points show data from autoclaved activated sludge (sorption controls). The dotted line represents the calculated LOQ for the experiment. Data from Experiment 4, see Table S1.3 for full experimental details.

## S2.1. Experimental optimization

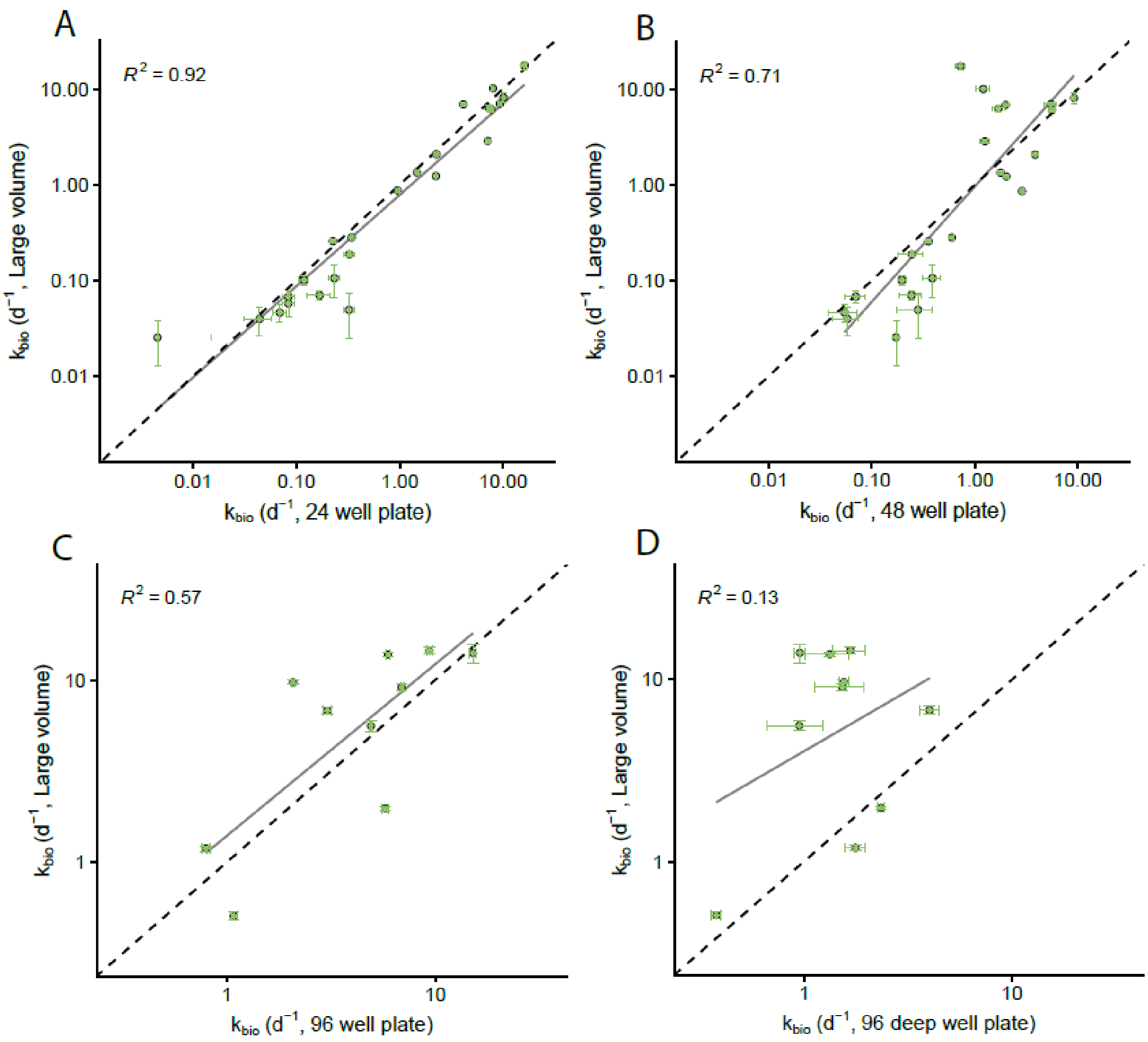


**Figure S2.3.** Correlation between $k_{deg}$ values calculated from large volume controls (y-axis) to $k_{deg}$ values from well plates. A. 24 well plate (data also shown in main text Figure 1A). B. 48 well plate. C. 96 well plate. D. 96 deep well plate. The dotted lines are 1:1 lines, and the grey lines are the lines of best fit. Data from Experiment 5 (24 well and 48 well) and Experiment 4 (96 well and 96 deep well).

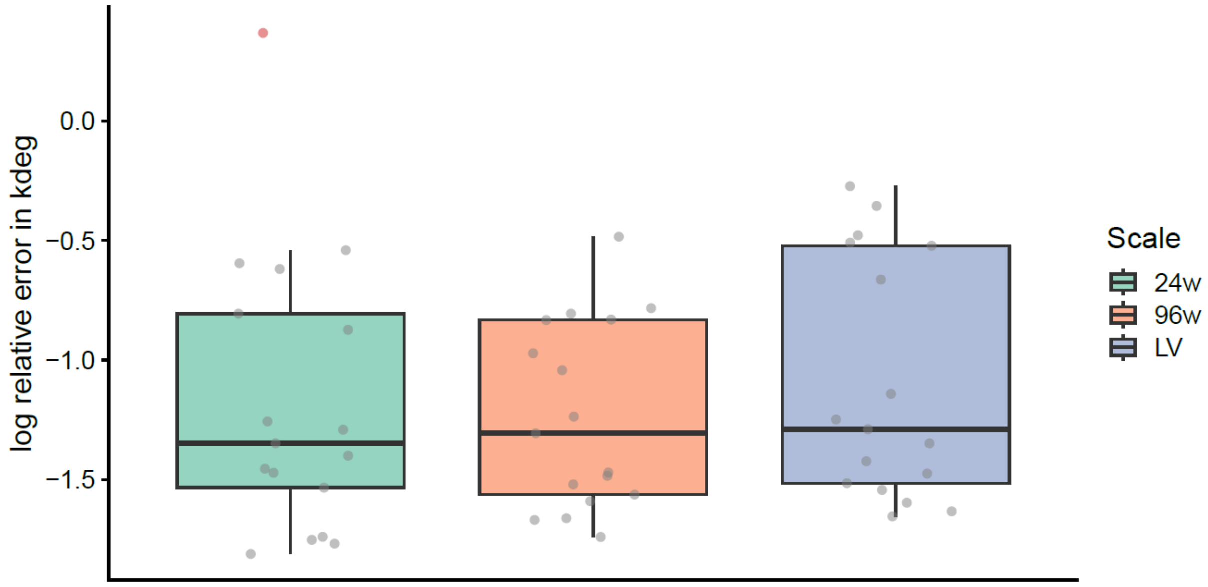


**Figure S2.4.** Comparison in the relative error in $k_{deg}$ values across all measurable compounds ($n_{exp}$ = 3 and $n_{compounds}$ = 17 for 96 well plate, and $n_{exp}$ = 4 and $n_{compounds}$ = 27 for 24 well plate and 50 mL control). 24- and 96-well plate scales consist of data from both the orbital shaker and the plate shaker; 50 mL control data is only from an orbital shaker. Using a mixed effects model, the differences between the three medians are not significant (24 well vs. 96 well, p = 0.826; 24 well vs 50 mL control, p = 0.205; 96 well vs. 50 mL control, p = 0.114). Outliers shown in red.

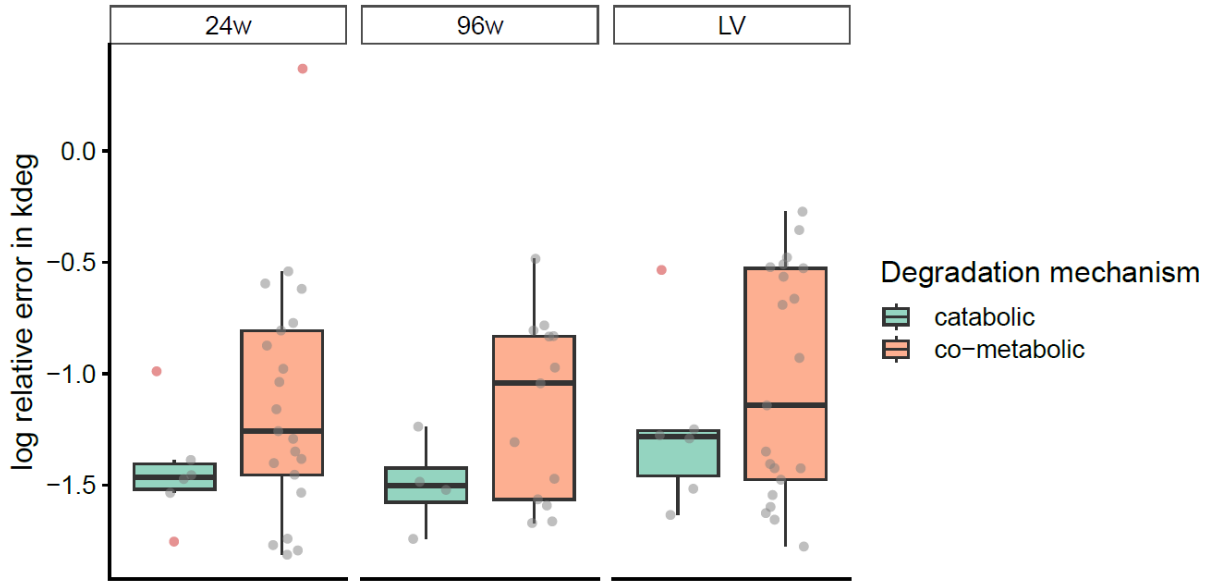


**Figure 2.5.** Comparison of the variability in $k_{deg}$ values between compounds suspected to be degraded catabolically by specific organisms (96 well plate: $n_{compounds}$ = 4; 24 well

plate: $n_{compounds}$ = 6; 50 mL control: $n_{compounds}$ = 6) and those suspected to be degraded co-metabolically (96 well plate: $n_{compounds}$ = 13; 24 well plate: $n_{compounds}$ = 21; 50 mL control: $n_{compounds}$ = 21). The differences between the medians are not significant, evaluated using a wilcox test (96 well, p = 0.202; 24 well, p = 0.14, 50 mL control, p = 0.55). Outliers are shown in red.

The following are compounds included in our spike mixture that are suspected to be at least partially catabolically degraded: bezafibrate, isoproturon, mefenamic acid, sulfamethoxazole, valsartan.[5] Note that the small sample sizes (n = 4 or 5 for suspected catabolically degraded compounds) mean that the statistical power to detect differences is limited. However, this initial analysis suggests no major differences in variability of $k_{deg}$ values calculated from compounds suspected to be degraded by two different mechanisms. This lends support to our conclusion that we are not observing a microbial lottery effect within our test system.

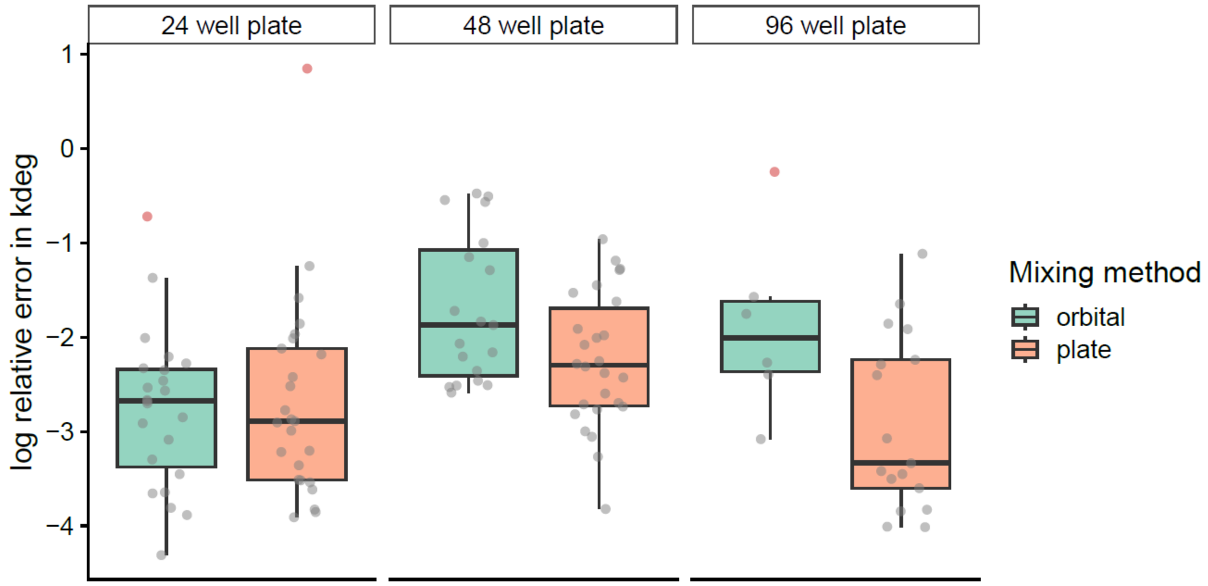


**Figure S2.6.** Relative error in $k_{deg}$ by mixing method and plate scale across all measurable compounds. Using a mixed effects model, for the 24 well plate scale the difference in medians is not significant (p = 0.95). For the 48 and 96 well plate scales the differences in medians are significant (p = 3.0 x $10^{-4}$ and 7.8 x $10^{-4}$, respectively). Outliers are shown in red.

To limit the loss of test compounds to well plate sorption during initial plating and to attempt to reduce inter-replicate variability in initial compound concentrations, we explored different methods of introducing the test compounds to the experimental vessels. While we had previously used plating (i.e., evaporation of ethanol and re-

dissolution) as the preferred spiking method in large volume experiments, our results showed that directly injecting the test compounds dissolved in ethanol is more effective in limiting initial diffusion into the well plate material in small volume vessels (SI Figure S2.6). We also observed a systematic bias in $k_{deg}$ values from the two spiking methods, with $k_{deg}$ values from the direct spike method being generally lower than those from the plating method (SI Figure S2.7), though we did not observe a decrease in variability in the calculated $k_{deg}$ values between the two compound addition methods (SI Figure S2.8). By directly spiking the test compounds, which are dissolved in ethanol, we artificially increase the dissolved organic carbon present in the system and speculated that the depression of $k_{deg}$ values in the direct spike method could either be due to inhibition of microorganisms at high solvent concentrations, or alternatively that the microorganisms might use ethanol as a preferential substrate. Comparing $k_{deg}$ values for different ethanol concentrations in 24 well plates, we saw that ethanol concentrations of up to 0.06% do not significantly impact the calculated $k_{deg}$ values for most of the compounds tested here (SI Figure S2.9). While the addition of small amounts of ethanol seems preferable compared to plating in small volume systems, freshly prepared spike solutions in as aqueous a medium as can be achieved is the best approach for test compound introduction.

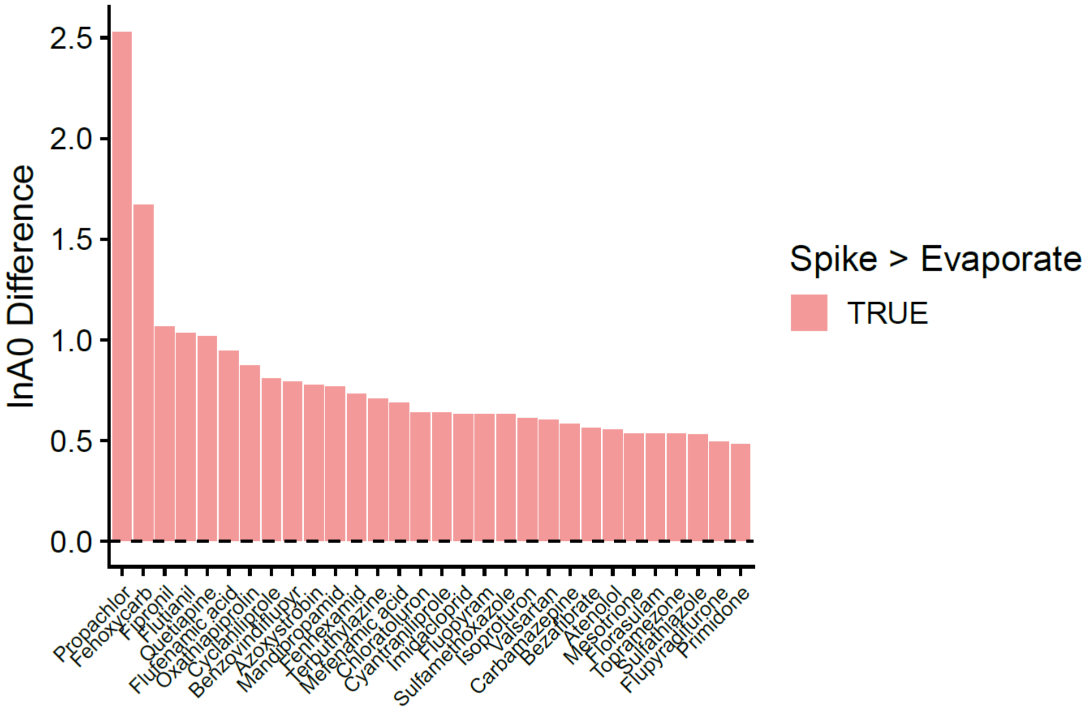


**Figure S2.7.** Difference between the initial ln(peak area) (lnA0) in the case where compounds were spiked directly vs. plated and the ethanol left to evaporate.

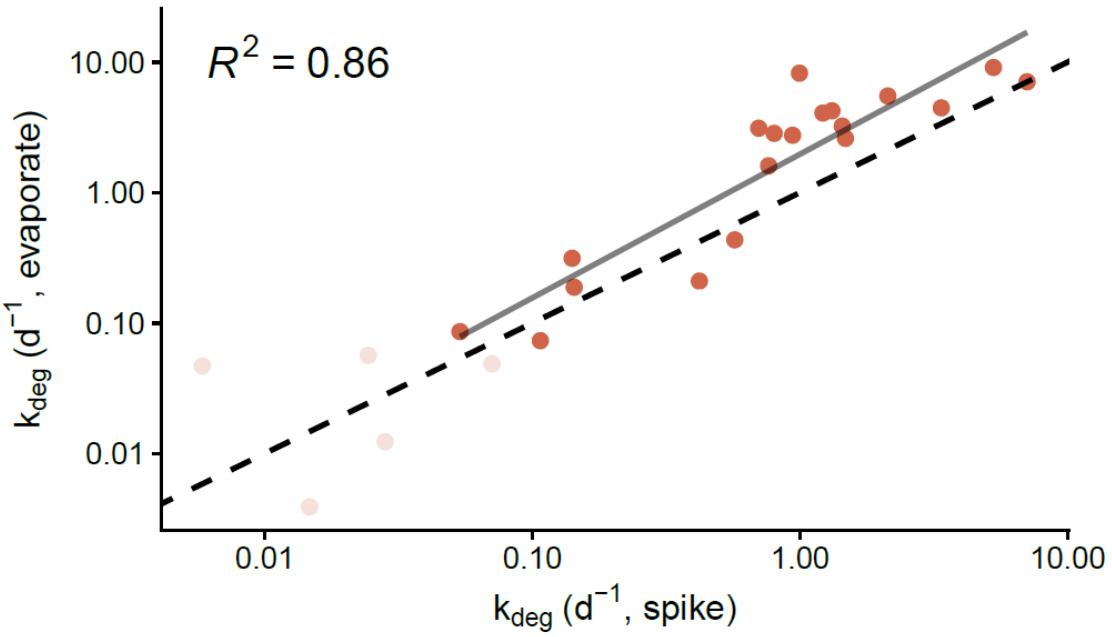


**Figure S2.8.** Correlation between $k_{deg}$ values calculated where compounds were spiked directly vs. where compounds were plated and the ethanol left to evaporate. The grey line is the line of best fit, and the dotted line is the 1:1 line.

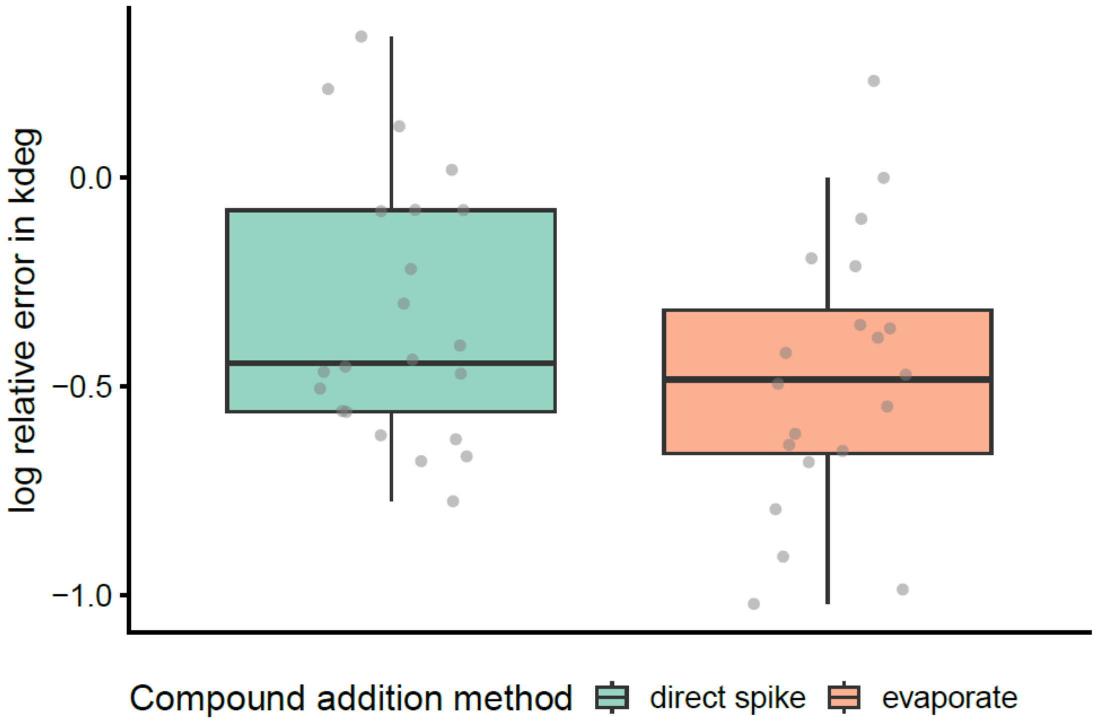


**Figure S2.9.** Comparison of relative error in $k_{deg}$ values between the two compound addition techniques. The difference in medians is not significant, calculated using a wilcox test ($p = 0.215$).

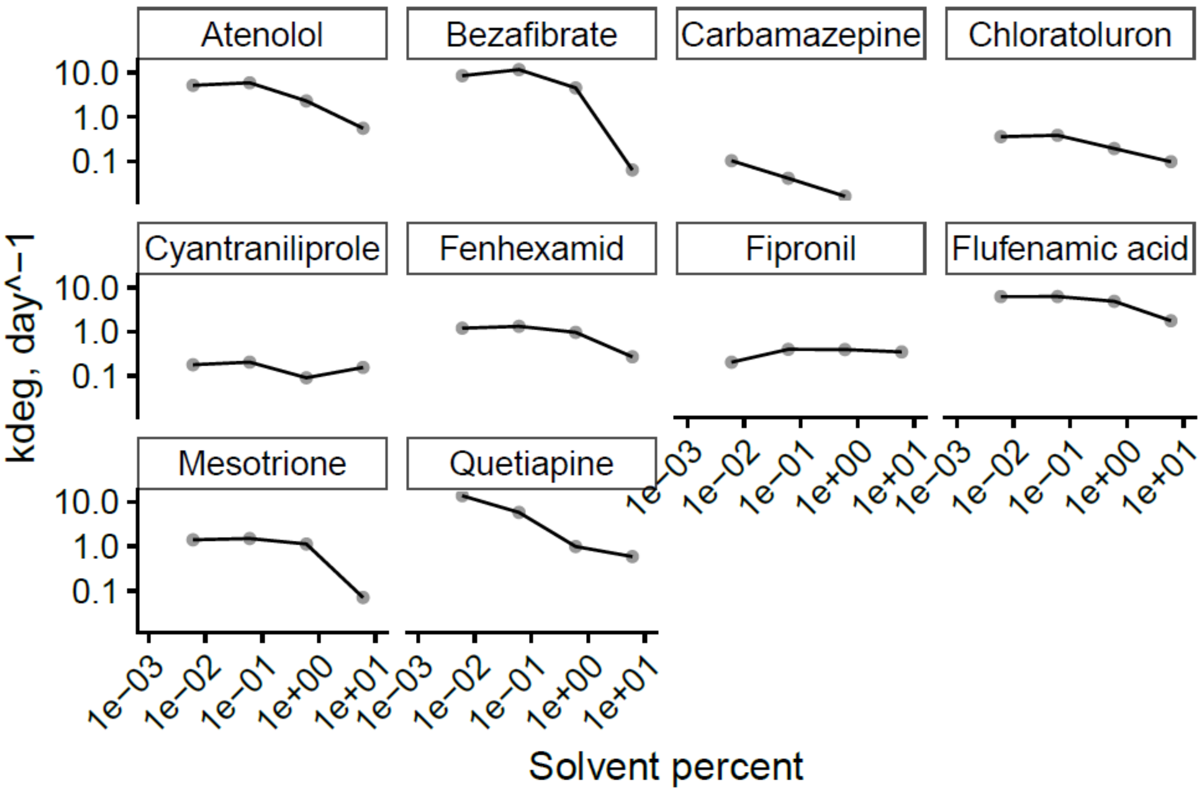


**Figure S2.10.** $k_{deg}$ values determined in experiments where compounds were directly spiked to the plates in mixes containing an increasing ethanol percent. Both x and y axis are log scaled.

## S2.2. Reduction of analytical and experimental effort

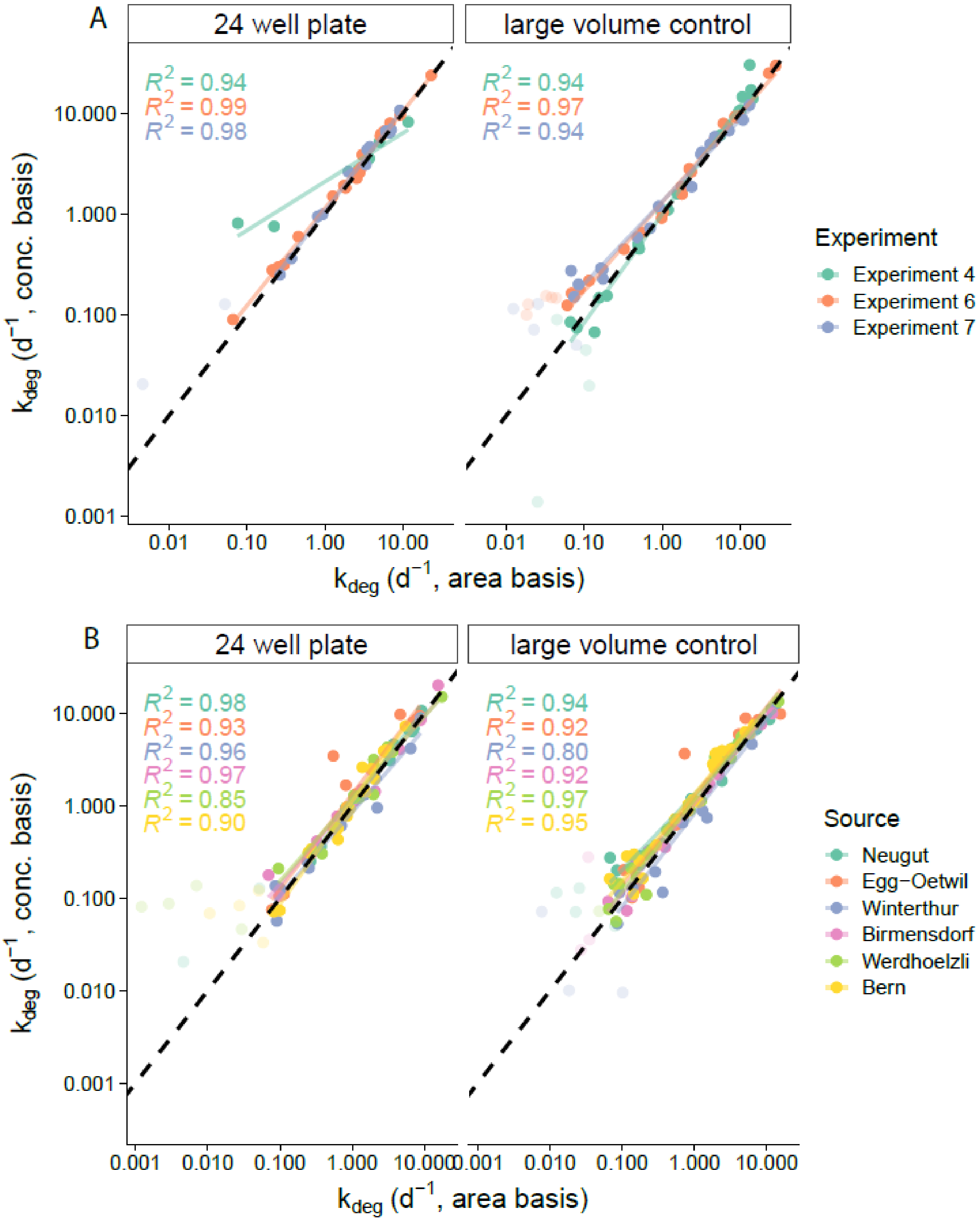


**Figure S2.11.** Correlation of $k_{deg}$ values calculated using compound peak areas and calculated concentrations for **A.** Neugut AS across three different experiments (See Table S1.3) and **B.** Six different WWTPs (See Table S1.2). Pale points represent $k_{deg}$ values smaller than $k_{deg,min}$, as defined in Section S1.3.

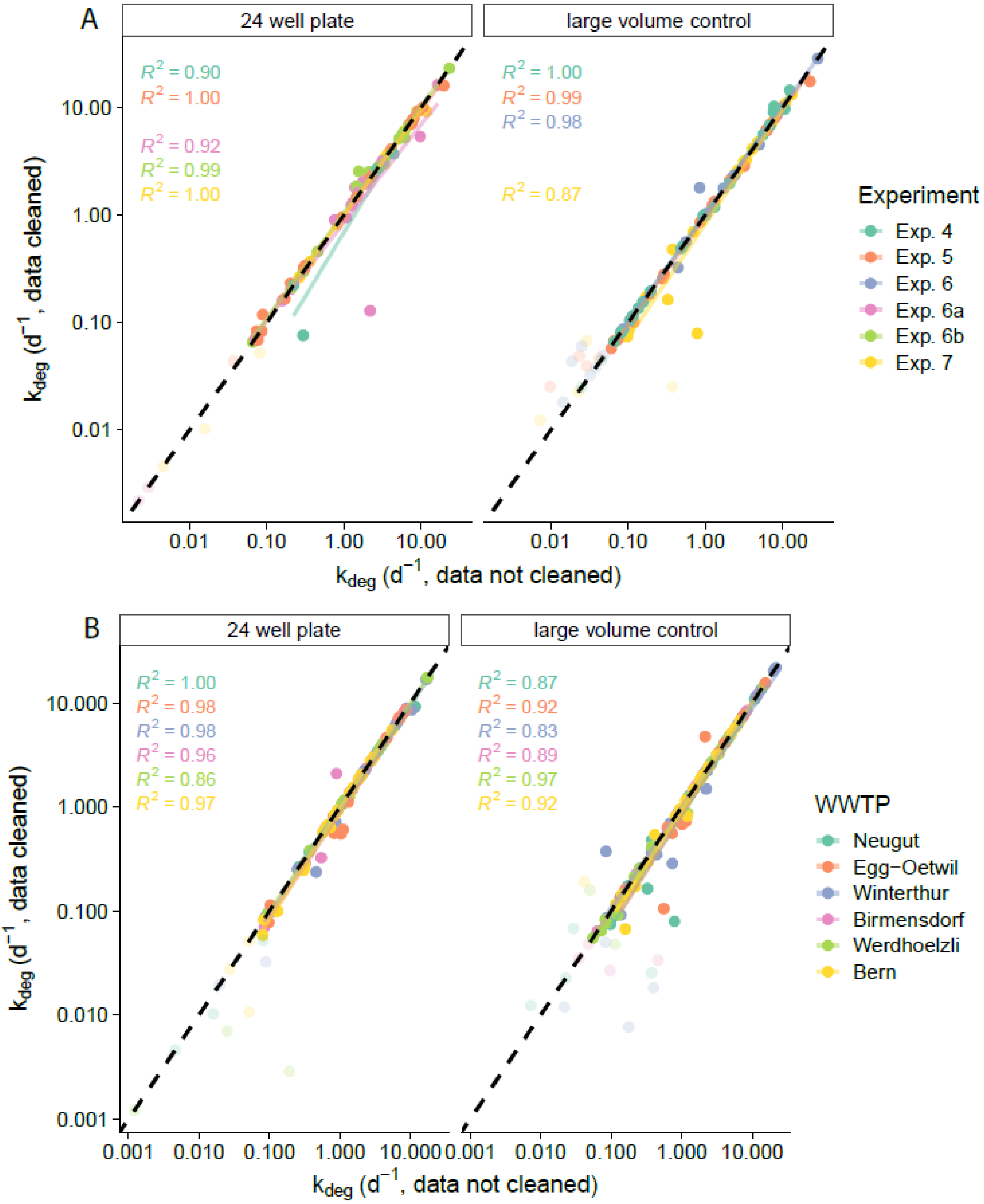


**Figure S2.12.** Correlation of $k_{deg}$ values obtained from peak areas in Skyline that were manually checked (y-axis) to those that were obtained from the default peak area integrations in Skyline (x-axis). Pale points represent $k_{deg}$ values smaller than $k_{deg,min}$, as defined in Section S1.4. **A.** Data for Neugut WWTP over time (See Table S1.3). **B.** Data from experiment 7 (See Table S1.3).

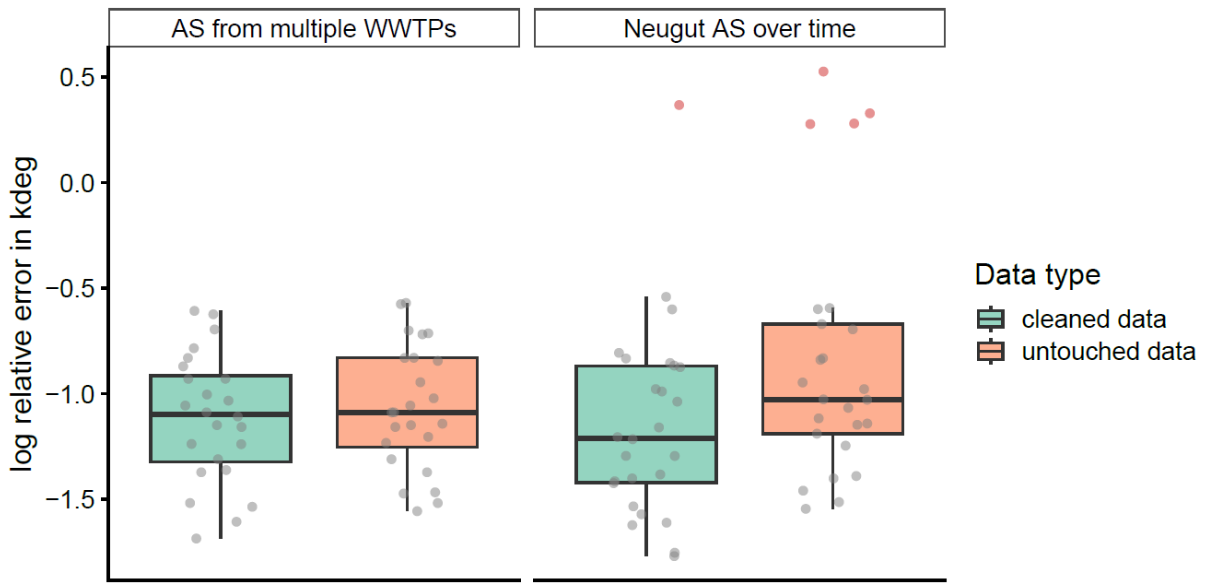


**Figure S2.13.** Comparison of the variability in $k_{deg}$ values for data cleaned in Skyline or taken from Skyline without any manual intervention. Data shown for all measurable compounds ($n_{compounds}$ = 29) in Neugut WWTP across four experiments spanning 1.5 years for 24 well plates, and for all measurable compounds ($n_{compounds}$ = 28) in five WWTPs at a single point in time for 24 well plates. The difference between the medians is not significant in both cases, evaluated using wilcox tests (AS from multiple WWTPS, p = 0.628; Neugut AS over time, p = 0.057). Outliers are shown in red.

For a minority of compounds (three out of 25), Skyline was not able to correctly integrate enough peaks within the time series to calculate a $k_{deg}$ value. $k_{deg}$ values for three additional compounds were incorrectly calculated due to missing peak integrations, creating outliers with high relative error when comparing compounds in Neugut sludge over time. These integration failures occurred even for high-quality peaks, suggesting software limitations rather than analytical detection issues, though the problem did not occur in every experiment. We are developing automated flagging criteria based on replicate peak variability (CV thresholds) and unexpected kinetic

trajectories to identify compounds requiring manual review, minimizing both data loss and incorrect calculations.

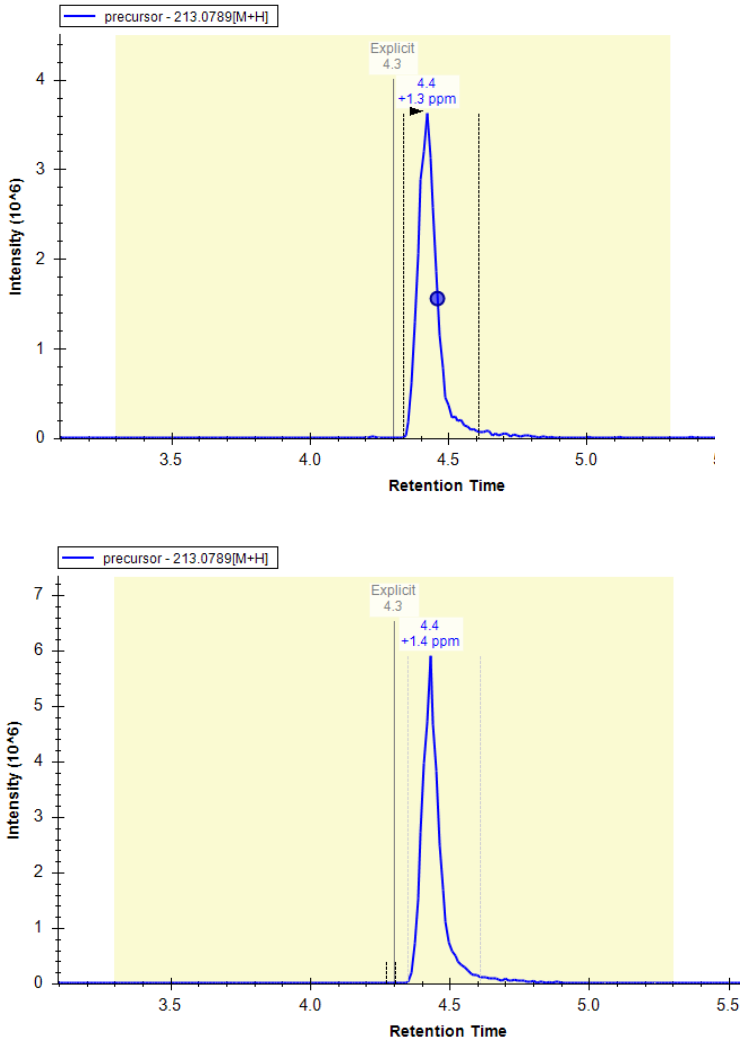


**Figure S2.14.** Comparison of correctly integrated peak (top) to peak missed by Skyline (bottom) for chloratoluron. The samples are replicates and were injected consecutively.

## S3. Implementation and future work

The relationship between physicochemical properties and detectability in our assays is complex, especially affecting strongly sorbing or hydrophobic compounds. Theoretically, compounds with log *K*oc > 4 should have less than 5% remaining in solution at a typical TSS of 2 g/L, but this threshold does not correspond perfectly to detectability. Some compounds with log *K*oc > 4 are detectable despite strong initial sorption (bezafibrate, cyclaniliprole, mandipropamid), while others within a log *K*oc range of 3 – 4 are not detectable (fenoxycarb, fipronil, flutianil).

Log D values often help explain these discrepancies. For instance, the low log D value of bezafibrate (0.73) calls into question its high predicted log *K*oc of 4.14 and helps to explain why we can detect this compound easily. Conversely, flutianil's high log D (4.48) indicates potentially strong hydrophobicity not reflected in its more moderate predicted log *K*oc of 3.28, partially rationalizing its non-detection.

While specialty chemicals are often designed for log *K*oc and log D ranges that fall within our detectable scope, some compounds still fall outside this applicability domain. For these compounds, distinguishing between sorption and rapid biotransformation remains challenging without explicit quantification. We are developing alternative experimental approaches, including full extraction and employing dilute sludge inocula, to address these limitations.

**Table S2.1.** Linking compound physicochemical properties with their measurability in the miniaturized assay. "Detect in bt @ t = 0?" asks whether we can detect the compound in biotransformation experiments in the first measured timepoint. "Detect in sc?" asks whether the compound is measurable (and stable over time) in 24 well plate and large volume sorption controls. "Disappears in 96wp sc over time?" asks whether the compound continues to disappear in the 96 well plate sorption control over the course of 24 hours. Answers in parentheses indicate that the compound was either generally not measurable, but measurable in one experiment (no in parentheses) or generally measurable, but not measurable in one experiment (yes in parentheses).

| Compound name | log *K*oc | log D @ pH 7.4 | Detect in calibration? | Detect in bt @ t = 0? | Detect in sc? | Disappears in 96wp sc over time? |
|---|---|---|---|---|---|---|
| Albuterol | 2.03 | -1.33 | no | | | |
| Atenolol | 2.48 | -1.26 | yes | yes | yes | no |
| Azoxystrobin | 2.77 | 2.50 | yes | yes | yes | yes |
| Benzovindiflupyr | 3.16 | 3.96 | yes | yes | yes | (yes) |
| Bezafibrate | 4.14 | 0.73 | yes | yes | yes | no |
| Bromoxynil | 2.48 | 0.80 | no | | | |
| Carbamazepine | 2.74 | 1.20 | yes | yes | yes | yes |
| Chloratoluron | 2.02 | 2.44 | yes | yes | yes | yes |
| Cyantraniliprole | 2.38 | 2.35 | yes | yes | yes | yes |
| Cyclaniliprole | 4.98 | 4.16 | yes | yes | yes | yes |
| Dicamba | 1.50 | -1.16 | no | | | |
| Diuron | 2.83 | 2.71 | no | | | |
| Fenhexamid | 2.68 | 3.79 | yes | yes | yes | yes |
| Fenoxycarb | 3.00 | 4.09 | yes | no | yes | (yes) |
| Fipronil | 3.72 | 3.69 | yes | (yes) | no | (yes) |
| Florasulam | 1.34 | -0.95 | yes | yes | yes | no |
| Fluconazole | 1.71 | 0.60 | no | | | |
| Flufenamic acid | 3.25 | 1.48 | yes | yes | yes | no |
| Fluopyram | 3.56 | 4.15 | yes | yes | yes | yes |

| | | | | | | |
|---|---|---|---|---|---|---|
| Flupyradifurone | 1.99 | 1.46 | yes | yes | yes | no |
| Flutianil | 3.28 | 4.48 | yes | no | no | yes |
| Imidacloprid | 1.87 | 0.47 | yes | yes | yes | (yes) |
| Isoproturon | 2.00 | 2.57 | yes | yes | yes | yes |
| Ketoprofen | 2.84 | $-3.8 \times 10^{-2}$ | yes | (yes) | yes | no |
| Kresoxim-methyl | 2.97 | 3.43 | yes | (no) | (no) | yes |
| Levetiracetam | 1.48 | -0.78 | yes | (yes) | yes | no |
| Mandipropamid | 4.16 | 3.48 | yes | yes | yes | yes |
| Mecoprop | 1.67 | -0.26 | no | | | |
| Mefenamic acid | 2.85 | 2.54 | yes | yes | yes | no |
| Mesotrione | 2.09 | 0.52 | yes | yes | yes | no |
| Metaflumizone | 4.49 | 5.85 | no | | | |
| Morphine | 5.32 | $-1.8 \times 10^{-2}$ | no | | | |
| Oxathiapiprolin | 3.99 | 4.37 | yes | (yes) | yes | yes |
| Pargyline | 2.10 | 2.39 | yes | no | no | |
| Primidone | 1.90 | 0.73 | yes | yes | (no) | no |
| Propachlor | 1.90 | 2.23 | yes | (yes) | yes | yes |
| Proquinazid | 2.81 | 3.16 | yes | (no) | (no) | yes |
| Pseudoephedrine | 2.11 | -0.72 | no | | | |
| Quetiapine | 3.05 | 2.29 | yes | yes | yes | yes |
| Sulfamethoxazole | 1.96 | -0.49 | yes | yes | yes | no |
| Sulfathiazole | 2.43 | -0.36 | yes | yes | yes | no |
| Terbuthylazine | 2.32 | 3.10 | yes | yes | yes | yes |
| Topramezone | 2.23 | 0.70 | yes | yes | yes | no |
| Valsartan | 2.77 | -2.26 | yes | yes | yes | no |
